# Blockade of CCR5^+^ T Cell Accumulation in the Tumor Microenvironment Optimizes Anti‐TGF‐β/PD‐L1 Bispecific Antibody

**DOI:** 10.1002/advs.202408598

**Published:** 2024-09-20

**Authors:** Ming Yi, Tianye Li, Mengke Niu, Yuze Wu, Bin Zhao, Zhuoyang Shen, Shengtao Hu, Chaomei Zhang, Xiaojun Zhang, Jing Zhang, Yongxiang Yan, Pengfei Zhou, Qian Chu, Zhijun Dai, Kongming Wu

**Affiliations:** ^1^ Department of Breast Surgery The First Affiliated Hospital College of Medicine Zhejiang University Hangzhou 310000 P. R. China; ^2^ Cancer Center Shanxi Bethune Hospital Shanxi Academy of Medical Science Tongji Shanxi Hospital Third Hospital of Shanxi Medical University Taiyuan 030032 P. R. China; ^3^ Department of Gynecology The Second Affiliated Hospital of Zhejiang University School of Medicine Hangzhou 310009 P. R. China; ^4^ Department of Medical Oncology The First Affiliated Hospital College of Medicine Zhejiang University Hangzhou 310000 P. R. China; ^5^ Department of Oncology Tongji Hospital of Tongji Medical College Huazhong University of Science and Technology Wuhan 430030 P. R. China; ^6^ Wuhan YZY Biopharma Co., Ltd Biolake, C2‐1, No.666 Gaoxin Road Wuhan 430075 P. R. China

**Keywords:** bispecific antibody, CCR5, cancer immunotherapy, combination therapy, tumor microenvironment, TGF‐β, PD‐L1

## Abstract

In the previous studies, anti‐TGF‐β/PD‐L1 bispecific antibody YM101 is demonstrated, with superior efficacy to anti‐PD‐L1 monotherapy in multiple tumor models. However, YM101 therapy can not achieve complete regression in most tumor‐bearing mice, suggesting the presence of other immunosuppressive elements in the tumor microenvironment (TME) beyond TGF‐β and PD‐L1. Thoroughly exploring the TME is imperative to pave the way for the successful translation of anti‐TGF‐β/PD‐L1 BsAb into clinical practice. In this work, scRNA‐seq is employed to comprehensively profile the TME changes induced by YM101. The scRNA‐seq analysis reveals an increase in immune cell populations associated with antitumor immunity and enhances cell‐killing pathways. However, the analysis also uncovers the presence of immunosuppressive CCR5^+^ T cells in the TME after YM101 treatment. To overcome this hurdle, YM101 is combined with Maraviroc, a widely used CCR5 antagonist for treating HIV infection, suppressing CCR5^+^ T cell accumulation, and optimizing the immune response. Mechanistically, YM101‐induced neutrophil activation recruits immunosuppressive CCR5^+^ T cells via CCR5 ligand secretion, creating a feedback loop that diminishes the antitumor response. Maraviroc then cleared these infiltrating cells and offset YM101‐mediated immunosuppressive effects, further unleashing the antitumor immunity. These findings suggest selectively targeting CCR5 signaling with Maraviroc represents a promising and strategic approach to enhance YM101 efficacy.

## Graphical abstract

Anti‐TGF‐β/PD‐L1 bispecific antibody YM101 enhances the anti‐tumor immune response but mediates the recruitment of immunosuppressive CCR5^+^ T cells through neutrophils. These cells highly express immunosuppressive molecules and undermine the immunotherapy efficacy. A combined treatment strategy with the CCR5 antagonist Maraviroc is proposed to counteract this negative feedback, thus amplifying YM101's antitumor potential and fostering its clinical application.

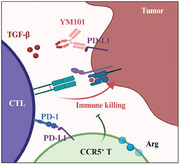

## 1 Background

Immunotherapy has emerged as a revolutionary paradigm in the realm of cancer treatment, offering new hope to patients with previously untreatable malignancies.^[^ ^1^, ^2^ ^]^ One of the most promising avenues within immunotherapy is the inhibition of programmed death protein‐1 (PD‐1) and its ligand PD‐L1, collectively referred to as anti‐PD‐1/PD‐L1 therapy.^[^ ^3^, ^4^, ^5^, ^6^, ^7^, ^8^ ^]^ This approach has not only showcased remarkable clinical success but has also led to enduring responses and extended survival across a spectrum of cancer types.^[^ ^9^ ^]^ Consequently, anti‐PD‐1/PD‐L1 agents have assumed a pivotal role in modern oncology, securing approvals for utilization in an expanding repertoire of malignancies, including non‐small cell lung cancer (NSCLC), breast cancer, and melanoma.^[^ ^10^, ^11^, ^12^, ^13^, ^14^, ^15^, ^16^, ^17^, ^18^ ^]^ Nevertheless, the profound potential of anti‐PD‐1/PD‐L1 therapy remains constrained by intrinsic and acquired resistance, necessitating a profound comprehension of the underlying mechanisms.^[^ ^19^, ^20^, ^21^, ^22^, ^23^, ^24^ ^]^

Transforming growth factor‐beta (TGF‐β) has emerged as a central protagonist in the narrative of resistance to anti‐PD‐1/PD‐L1 therapy.^[^ ^25^, ^26^, ^27^, ^28^ ^]^ In its capacity as a multifaceted cytokine, TGF‐β wields pleiotropic influence over various constituents of the tumor microenvironment (TME), encompassing tumor‐infiltrating lymphocytes (TILs), dendritic cells (DCs), macrophages, neutrophils, and cancer‐associated fibroblasts (CAFs).**^[^** ^29^, ^30^, ^31^ ^]^ Recent research endeavors have illuminated the pivotal role played by TGF‐β in nurturing an immunosuppressive milieu, thereby contributing to the resilience of cancer cells against the effects of anti‐PD‐1/PD‐L1 therapy.**^[^** ^32^ ^]^ Comprehending the intricate interplay between the TGF‐β pathway and the PD‐1/PD‐L1 axis is crucial in the development of strategic interventions to mitigate treatment resistance and augment immunotherapy efficacy.**^[^** ^33^, ^34^ ^]^

Recognizing the substantial impact of TGF‐β on the immune landscape within tumors and its consequent influence on anti‐PD‐1/PD‐L1 therapy, researchers have embarked on the development of dual‐blockade therapies that concurrently target the TGF‐β pathway and the PD‐1/PD‐L1 axis.**^[^** ^35^ ^]^ These approaches hold great promise in dismantling the collagen barrier erected by TGF‐β while synergistically augmenting the antitumor immune response mediated by PD‐1/PD‐L1 blockade.^[^ ^36^ ^]^ Numerous preclinical and clinical studies have been initiated to assess the safety and efficacy of these anti‐PD‐1/PD‐L1 combined with TGF‐β blockade regimens.^[^ ^37^, ^38^, ^39^, ^40^, ^41^ ^]^

Our previous work unveiled the world's first anti‐TGF‐β/PD‐L1 bispecific antibody (BsAb), known as YM101.^[^ ^42^ ^]^ YM101 demonstrated remarkable efficacy against various tumor models, particularly those categorized as immune‐excluded tumors. However, despite its substantial advantages over anti‐PD‐L1 monotherapy, YM101 frequently failed to achieve a complete response (CR) in the majority of cases. It has been well‐established that YM101 plays a pivotal role in enhancing T‐cell infiltration and facilitating the transformation of immune‐excluded tumors into inflamed ones. Nevertheless, the precise alterations in immune cell profiling following YM101 treatment have remained elusive. The ability to identify and manipulate immune cell subsets associated with YM101's efficacy holds significant promise for improving its performance. Therefore, a comprehensive evaluation of the TME is meaningful for the successful translation of anti‐TGF‐β/PD‐L1 BsAb into clinical practice. Such investigations are vital for overcoming the current resistance challenges and improving the landscape of cancer immunotherapy.

## 2 Results

### 2.1 Enhanced Antitumor Activity of anti‐TGF‐β/PD‐L1 BsAb YM101

**Figure 1:**
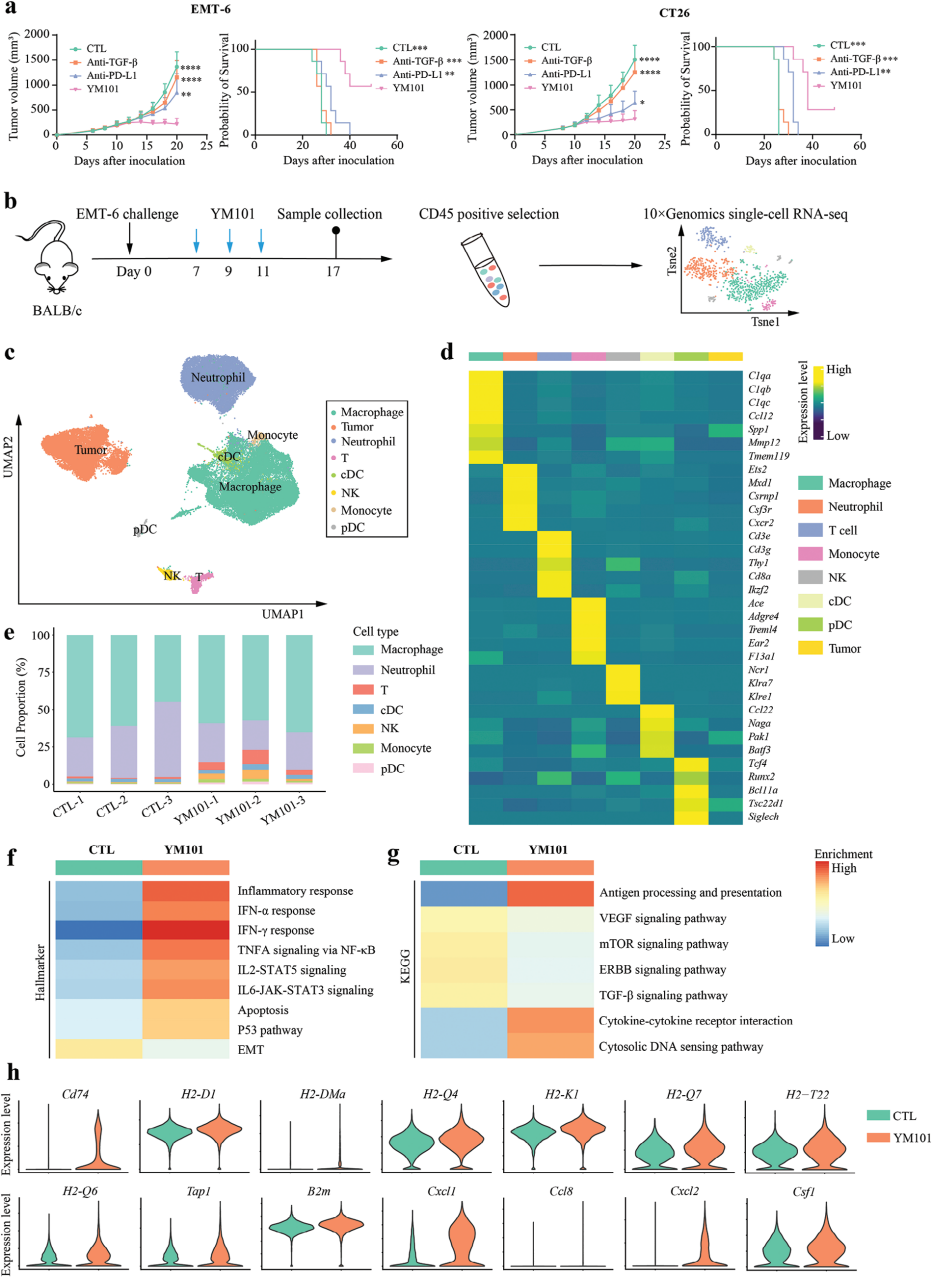
The enhanced antitumor activity of YM101. Mice were randomized into distinct treatment groups when tumor volumes reached 100 mm^3^, with seven mice per group. Treatment commenced at this point and continued for six doses. For antibody treatments, tumor‐bearing mice received equimolar quantities of hIgG (6.6 mg kg^−1^), PD‐L1 blocking antibody (6.6 mg kg^−1^), TGF‐β neutralizing antibody (6.6 mg kg^−1^), or YM101 (9 mg kg^−1^) every 2 days via intraperitoneal injection (n = 7). a) The tumor growth and survival curves of tumor‐bearing mice. b) The schematic diagram of the single‐cell RNA sequencing (scRNA‐seq) project. Following three doses of YM101 administration, fresh EMT‐6 tissues were collected. Tumor tissues underwent treatment with the dissociation buffer at 37 °C for 40 min. Subsequently, nanobeads for CD45 positive selection were utilized to isolate sufficient immune cells for scRNA‐seq. Living cells were then enriched via fluorescent cell sorting. Cells from two mice within the same group were pooled to form one sample, which was then loaded onto a 10× Genomics Chromium Controller. c) Uniform manifold approximation and projection (UMAP) plot for the TME components of EMT‐6 tumors. d) Heatmap showing the expression levels of cluster‐specific genes. e) Histogram representing the proportions of different types of immune cells in each sample. f) Heatmap depicting GSEA results of tumor cells based on Hallmarker sets. g) Heatmap depicting GSEA results of tumor cells based on KEGG sets. h) Violin plots showing genes significantly upregulated in the tumor cells of the YM101 group. Statistical analyses were performed using Student's *t*‐test and Log‐rank test (a). Significance is indicated as: ^**^ *p* < 0.01, ^***^ *p* < 0.001, ^****^ *p* < 0.0001.

YM101 exhibited robust antitumor activity across various murine tumor models, including EMT‐6 and CT26. Remarkably, both tumor models, typically unresponsive to anti‐PD‐1/PD‐L1 therapies partially due to TGF‐β‐mediated immune evasion, displayed significant tumor growth retardation upon YM101 treatment. This effect surpassed the performances of individual anti‐TGF‐β and anti‐PD‐L1 therapies, aligning with our previous findings. Moreover, during the seven‐week observation period, YM101 substantially prolonged the survival of tumor‐bearing mice, notably surpassing outcomes achieved by other treatment groups (**Figure** **1**a). Collectively, the antitumor potency of YM101 exceeded that of its parental antibodies. However, it is worth noting that YM101 therapy did not achieve a complete cure for most tumor‐bearing mice, with some succumbing to the high tumor burden.

### 2.2 Single‐Cell Transcriptome Profiling of the TME after YM101 Treatment

After administering three doses of YM101, we harvested fresh EMT‐6 tumors for single‐cell RNA sequencing (scRNA‐seq) (Figure 1b). Subsequently, through unsupervised clustering analysis, we categorized cells into 31 clusters. Leveraging the *SingleR* and *InferCNV* packages and established markers, we identified eight distinct cellular lineages, including macrophages, neutrophils, monocytes, NK cells, T cells, cDCs, pDCs, and tumor cells (Figure 1c–e). Based on the results of distribution preference analysis, T cells, NK cells, monocytes, pDCs, and cDCs showed a strong distribution preference in YM101‐treated tumors, whereas neutrophils were enriched in isotype antibody‐treated tumors (referred to as CTL) (Figure S1, Supporting Information). Consistent with prior studies highlighting YM101's capacity to augment immune cell infiltration and revive antitumor responses, we examined the state of tumor cells. Gene set enrichment analysis (GSEA) based on hallmark gene sets revealed enriched inflammatory response‐associated pathways (IFN‐α, IFN‐γ, TNF, IL‐2, and IL‐6 signaling) in the YM101‐treated group. Additionally, apoptosis and P53 pathways exhibited higher enrichment scores, potentially linked to enhanced tumor‐killing efficacy mediated by YM101. Conversely, the enrichment score of epithelial‐mesenchymal transition (EMT) was notably lower in the YM101 group, indicative of hampered TGF‐β signaling (Figure 1f). GSEA based on KEGG datasets indicated enrichment in antigen processing and presentation, cytokine‐cytokine receptor interaction, and cytosolic DNA sensing pathways within the YM101 group. Conversely, VEGF, mTOR, ERBB, and TGF‐β signaling pathways displayed significantly reduced enrichment scores (Figure 1g). At the transcriptional level, YM101 markedly upregulated the expression of genes encoding antigen‐presentation machinery elements *(Cd74*, *H2‐D1*, *H2‐DMa*, *H2‐Q4*, *H2‐K1*, *H2‐Q7*, *H2‐T22*, *H2‐Q6*, *Tap1*, and *B2m*) and pro‐inflammatory cytokines (*Cxcl1*, *Ccl8*, *Cxcl2*, and *Csf1*) (Figure 1h). This data collectively suggests that YM101 not only promotes tumor cell apoptosis by restoring antitumor responses but also elevates pro‐inflammatory mediators and enhances tumor cell antigen presentation, thereby fortifying the entire cancer‐immunity cycle.

### 2.3 Secondary Analyses of Multiple Immune Cells

**Figure 2:**
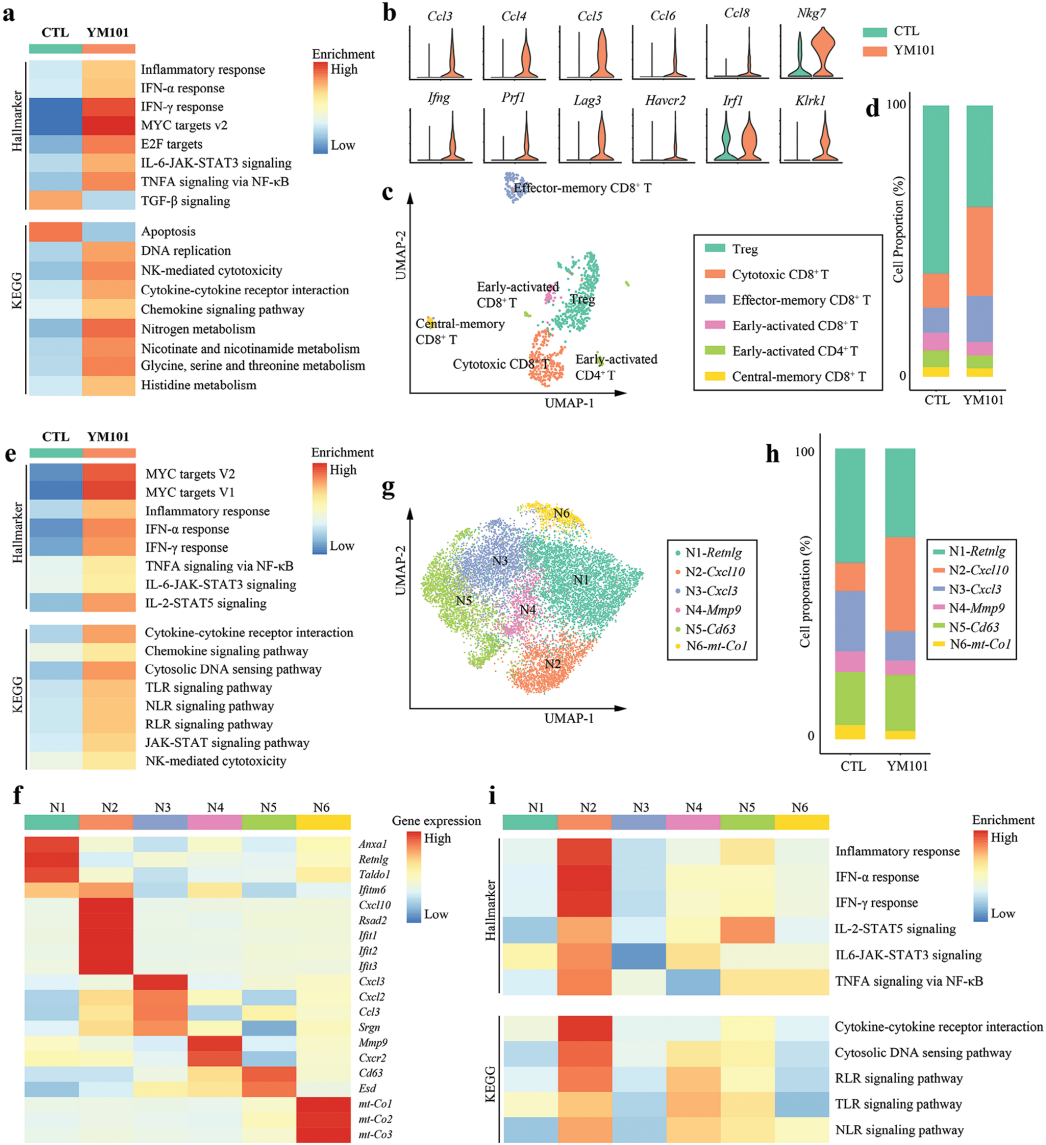
Secondary analysis of tumor‐infiltrating T cells and neutrophils. a) Heatmap depicting GSEA results of total T cells. b) Violin plots showing genes significantly upregulated in the T cells of the YM101 group. c) Uniform manifold approximation and projection (UMAP) plot showing the results of reclustering analysis of T cells. d) Histogram representing the proportions of T cell subsets in the CTL and YM101 group. e) Heatmap showing GSEA results of total neutrophils. f) Heatmap showing the expression levels of common neutrophil markers. g) Uniform manifold approximation and projection (UMAP) plot showing the results of reclustering analysis of neutrophils. h) Histogram representing the proportions of neutrophil subsets in the CTL and YM101 groups. i) Depicting the intrinsic features of neutrophil subsets by GSEA.

To accurately discern the roles of various immune cell subsets in YM101 therapy, we conducted secondary analyses encompassing multiple immune components. Initially, we explored the functional disparities between the YM101 and CTL groups across the entire T‐cell population. Results from GSEA illuminated the increased enrichment scores of pro‐inflammatory signaling, metabolic pathways, and cell proliferation signaling in T cells from the YM101 group. Conversely, YM101 diminished the enrichment scores of TGF‐β and apoptosis signaling within T cells (**Figure** **2**a). Furthermore, YM101 augmented the levels of genes responsible for chemokines, cytokines, and cell‐killing molecules within T cells (Figure 2b). Subsequently, we performed reclustering analyses of T cells, identifying six T‐cell subpopulations, including Treg, cytotoxic CD8^+^ T cells, effector‐memory CD8^+^ T cells, early‐activated CD8^+^ T cells, early‐activated CD4^+^ T cells, and central‐memory CD8^+^ T cells (Figure 2c). Annotations for T‐cell subsets relied on common markers, with *Foxp3* ^+^ designating Treg, *Gzmb* ^+^ *Prf1* ^+^ for cytotoxic CD8^+^ T cells, *Cd44* ^+^ *Sell* ^+^ for central‐memory T cells, *Cd44* ^+^ *Sell* ^−^ for effector‐memory T cells, and *Cd69* ^+^ for early‐activated T cells (Figure S2, Supporting Information). Cytotoxic CD8^+^ T cells and effector‐memory CD8^+^ T cells showed preferential enrichment in YM101‐treated tumors, while Tregs were CTL‐enriched (Figure 2d) (Figure S3, Supporting Information).

NK cells were stratified into immature and mature NK‐cell subsets. Although no significant difference was observed in the ratio between these subsets, YM101 notably expanded the absolute cell count of mature NK cells. Here, *Itgam* ^+^ *Cd27* ^−^ cells were designated as mature NK cells, while others were characterized as immature NK cells (Figure S4a–d, Supporting Information). Generally, NK cells from the YM101 group displayed enriched pro‐inflammatory pathways, encompassing inflammatory response, IFN‐α, IFN‐γ, TNF, IL‐2, IL‐6, JAK‐STAT, Toll‐like receptor (TLR), T‐cell receptor (TCR), B‐cell receptor (BCR), chemokine, and cytokine signaling (Figure S4e, Supporting Information), indicative of enhanced functions and activities.

Tumor‐infiltrating macrophages constitute a highly heterogeneous group within the TME.^[^ ^43^ ^]^ Collectively, macrophages within the YM101 group exhibited enrichment in immune‐supportive pathways, including inflammatory response, various cytokine or chemokine pathways, antigen presentation and processing, TLR, NOD‐like receptor (NLR), RIG‐I‐like receptor (RLR), and cytosolic DNA sensing pathways (Figure S5a, Supporting Information). These macrophages were categorized into ten subpopulations based on specific markers, including macrophage‐*Mki67*, M1‐*Il1b*, M1‐*Rsad2*, M2‐*Sparc*, M2‐*S100a8/9*, M2‐*Mmp9*, M2‐*Mmp12*, M2‐*Malat1*, M2‐*Fn1*, and M2‐*Siglec1*. While M1‐like subsets are associated with immunostimulatory roles, M2‐like subsets are linked to immunoinhibitory functions in classical immunological paradigms. GSEA results indicated that the two M1‐like subsets exhibited robust pro‐inflammatory characteristics (Figure S5b–e, Supporting Information). Notably, all M1‐like macrophage subsets showed a distribution preference in YM101‐treated tumors, but some M2‐like macrophage subsets, such as M2‐*Mmp12* and M2‐*Fn1*, were CTL‐enriched (Figure S6, Supporting Information).

Similarly, cDCs within the YM101 group displayed higher enrichment scores in inflammatory response, antigen presentation, and processing, as well as innate immune sensing pathways (TLR and RLR). Conversely, the enrichment scores for Wnt, mTOR, and vascular endothelial growth factor (VEGF) signaling pathways were markedly lower in the YM101 group (Figure S7a, Supporting Information). Total cDCs were further classified into six subclusters based on common markers:^[^ ^44^ ^]^ cDC1‐*Clec9a*, cDC1‐*Ccl22*, cDC2‐*Itgax*, cDC2‐*Cd209a*, cDC‐*Lyz2*, cDC‐*S100a8/9*. Among them, cDC1‐*Clec9a* and cDC2‐*Cd209a* possessed enhanced immune stimulation and antigen presentation capabilities (Figure S7b–d, Supporting Information). Analysis revealed that these two subsets were preferentially enriched in tumors treated with YM101 (Figure S8, Supporting Information). In sum, YM101 treatment effectively invigorated antigen presentation cells (APCs) within the TME.

Furthermore, a minor population of monocytes was identified in this model. Overall, YM101 endowed monocytes with heightened pro‐inflammatory signaling (e.g., TNF, IFN‐α, IFN‐γ, IL‐2, and IL‐6), antigen presentation and processing capabilities, innate immune sensing pathways (NLR and RLR), and phagocytosis. However, TGF‐β signaling was suppressed in monocytes of the YM101 group (Figure S9a, Supporting Information). Monocytes were categorized into three groups based on specific markers: monocyte‐*Ly6c2*, monocyte‐*Cd74*, and monocyte‐*S100a8/9*. Among the three subpopulations, monocyte‐*Cd74* exhibited the highest expression of MHC‐coding genes, followed by monocyte‐*Ly6c2* and monocyte‐*S100a8/9*. GSEA revealed that the monocyte‐*Cd74* subset possessed the most potent antigen presentation capabilities, while the monocyte‐*S100a8/9* subset exhibited the weakest antigen presentation capacity (Figure S9b–e, Supporting Information). Contrary to monocyte‐*Ly6c2*, the monocyte‐*S100a8/9* subset was enriched in CTL (Figure S10, Supporting Information). These data suggested that YM101 reduced monocytes with poor presentation capabilities, contributing to an overall increase in the immunostimulatory potential of the monocyte population.

Neutrophils also constituted a highly complex and heterogeneous population within the TME. Concerning total neutrophils, YM101 treatment resulted in increased inflammatory response and innate immune sensing pathways (TLR, NLR, RLR, and cytosolic DNA sensing) (Figure 2e). Based on common markers, the neutrophil subset was classified into six subgroups: N1‐*Retnlg*, N2‐*Cxcl10*, N3‐*Cxcl3*, N4‐*Mmp9*, N5‐*Cd63*, and N6‐*mt‐Co1* (Figure 2f–h). GSEA indicated that the N2‐*Cxcl10* subset displayed the most robust inflammatory response and innate immune sensing signaling (Figure 2i). Further analysis showed that, relative to other subtypes of neutrophils, N2‐*Cxcl10* neutrophils showed a strong distribution preference in YM101‐treated tumors (Figure S11, Supporting Information). Collectively, our data suggested that YM101 induced the accumulation of activated neutrophils within the TME.

### 2.4 YM101‐Enhanced T Cell Communication Network

**Figure 3:**
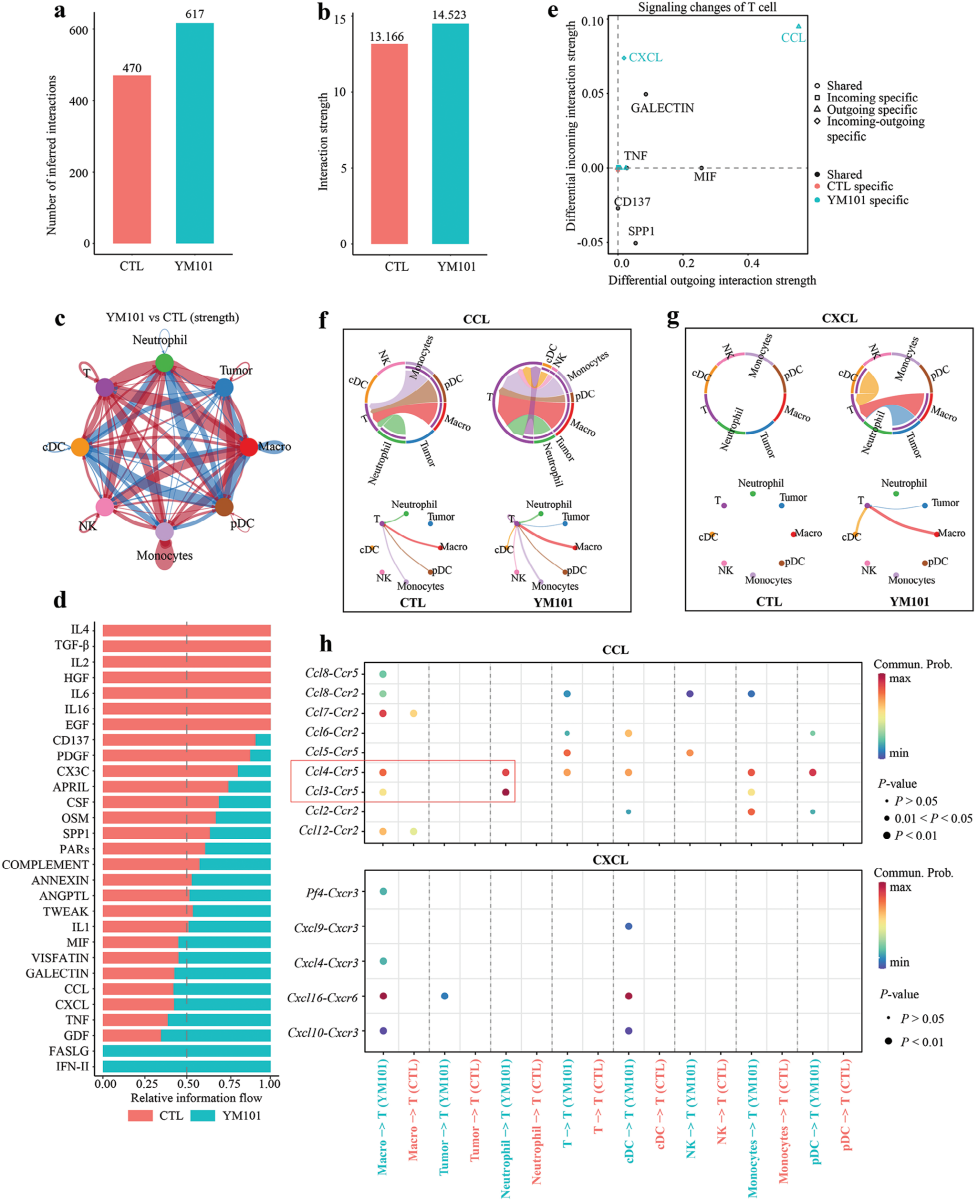
Intercellular ligand‐receptor prediction among tumor‐infiltrating T cells and other cells revealed by *CellChat* package. a,b) Bar plots showing the number and strength of intercellular interactions in the CTL and YM101 groups. c) The size of the circles corresponds to the counts of different cell lineages, while the thickness of the edges signifies the intensity of interaction between these populations. The loops colored red were strengthened in the YM101 group, and these colored blue loops were strengthened in the CTL group. d) Differential incoming or outgoing signaling patterns of T cells in the CTL and YM101 groups. The bars colored red were the relative strength of the CTL group, and the colored blue bars were the relative strength of the YM101 group. e) Scatter plots presenting differential signaling patterns of T cells in CTL and YM101 groups. The signaling pathways colored red were specific in the CTL group, and these colored blue pathways were specific in the YM101 group. f) Chord diagrams and circle plots of the inferred CCL signaling flow targeting T cells. g) Chord diagrams and circle plots of the inferred CXCL signaling flow targeting T cells. h) Bubble plots depict potential ligand‐receptor pairs contributing to the enhanced CCL and CXCL signaling flows in the YM101 group. Dot color signifies communication probabilities, while dot size corresponds to calculated *p*‐values. The absence of dots indicates zero communication probability. The *p*‐values are calculated using a two‐sided permutation test.

To elucidate cell‐to‐cell interactions among various TME components, we employed the *CellChat* package to infer biologically significant cell communication. Notably, we observed extensive cell communication networks, particularly involving macrophages and other cell types (Figure S12, Supporting Information). The YM101 group exhibited an overall growth in cell communications, encompassing both interaction number and strength (**Figure** **3**a–c). Intriguingly, YM101 amplified cell communications within pro‐inflammatory and cell‐killing pathways, which included IFN‐II, Fas ligand (FASLG), growth differentiation factor (GDF), TNF, C‐C motif chemokine ligand (CCL), and C‐X‐C motif chemokine ligand (CXCL). Conversely, YM101 diminished cell‐to‐cell interactions within IL‐4 and TGF‐β signaling pathways (Figure 3d). We then identified cell communications specific to the YM101 and CTL groups across individual cell types (Figure S13, Supporting Information). Within T cells, we detected biologically significant cell communications within CCL and CXCL pathways, exclusive to the YM101 group (Figure 3e). Given the pivotal role of T cells in antitumor responses, we further scrutinized cell‐to‐cell interactions between T cells and other cell types within CCL and CXCL pathways. For CCL signaling, we observed limited interactions between T cells and macrophages/pDC/monocytes in the CTL group, whereas YM101 significantly bolstered communication between T cells and myeloid cells, particularly macrophages, pDCs, and neutrophils (Figure 3f). Similarly, CXCL signaling exhibited scarce biologically significant cell communication in the CTL group. However, following the YM101 treatment, interactions between T cells and myeloid cells were markedly intensified (Figure 3g).

Subsequently, to precisely depict incoming and outgoing signaling patterns for individual cell types, we calculated and visualized the strength of signaling flow based on the *CellChat* package. As described earlier, the strength of incoming CXCL and CCL signaling flow was notably stronger in T cells of the YM101 group (Figure S14, Supporting Information). The observed signaling flow pattern suggests that tumor‐infiltrating T cells may be stimulated through paracrine CXCL and CCL signaling, thereby contributing to the heightened efficacy of YM101. Finally, to identify specific ligand‐receptor pairs contributing to the heightened CXCL and CCL signaling flow, we analyzed the expression levels of all ligand‐receptor pairs associated with CXCL and CCL signaling and calculated the corresponding probability of cell communication. Our results revealed that YM101‐induced enhancement of T‐cell communications largely depended on CCR2, CCR5, and CXCR6 signaling. In particular, the CCL3/4‐CCR5 signaling (from neutrophil to T cell) and the CXCL16‐CXCR6 signaling (from macrophage/cDC to T cell) were significantly enhanced after YM101 treatment (Figure 3h). Our data suggested that YM101 might stimulate various cells within the TME to secrete CCL and CXCL molecules, thus attracting the recruitment and regulating the functions of CCR2^+^, CCR5^+^, and CXCR6^+^ T cells.

### 2.5 The Intrinsic Features of *Ccr2* ^+^, *Ccr5* ^+^, *Cxcr6* ^+^ T Cells in the TME

**Figure 4:**
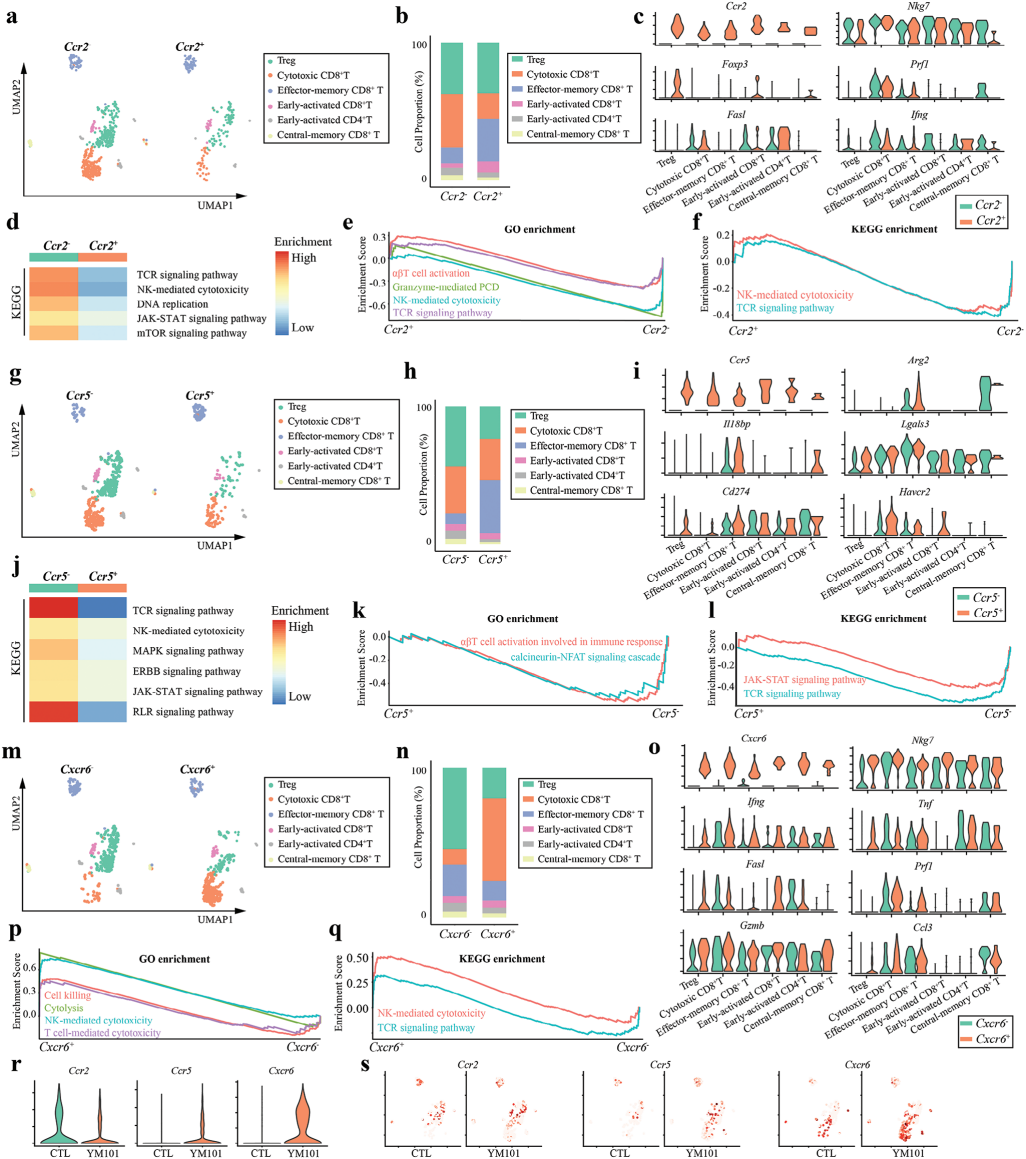
The intrinsic features of *Ccr2* ^+^, *Ccr5* ^+^, and *Cxcr6* ^+^ T cells of the YM101 group. a) Uniform manifold approximation and projection (UMAP) clustering for *Ccr2* ^−^ and *Ccr2* ^+^ T cells after YM101 treatment. In total 692 T cells, 495 were *Ccr2* ^−^ (71.5%), and 197 were *Ccr2* ^+^ (28.5%) T cells. b) Histogram representing the proportions of T‐cell subsets in the *Ccr2^−^* and *Ccr2^+^* T cells of the YM101 group. c) Violin plots showing the expression levels of T cell activation‐associated markers. d) Heatmap showing enrichment analysis results of *Ccr2^−^* and *Ccr2^+^* T cells of the YM101 group. e,f) GSEA enrichment plots showing the differences between *Ccr2^−^* and *Ccr2^+^* T cells of the YM101 group. g) UMAP plot showing the results of reclustering analysis of T cells according to *Ccr5* level. h) Histogram representing the proportions of T‐cell subsets in the *Ccr5^−^* and *Ccr5^+^* T cells of the YM101 group. i) Violin plots showing the expression levels of immune suppression‐associated markers. j) Heatmap showing enrichment analysis results of *Ccr5^−^* and *Ccr5^+^* T cells of the YM101 group. k,l) GSEA enrichment plots showing the differences between *Ccr5^−^* and *Ccr5^+^* T cells of the YM101 group. m) UMAP plot showing the results of reclustering analysis of T cells according to *Cxcr6* level. n) Histogram representing the proportions of T‐cell subsets in the *Cxcr6^−^* and *Cxcr6^+^* T cells of the YM101 group. o) Violin plots showing the expression levels of T cell activation and cytotoxicity‐associated markers. p,q) GSEA enrichment plots showing the differences between *Cxcr6^−^* and *Cxcr6^+^* T cells of the YM101 group. r) Violin plots showing the expression levels of *Ccr2*, *Ccr5*, and *Cxcr6* in T cells of the CTL and YM101 groups. s) UMAP plots showing the expressions of *Ccr2*, *Ccr5*, and *Cxcr6* in T cells of the CTL and YM101 groups.

Given the role of CCL and CXCL pathways in T‐cell chemotaxis and recruitment, we hypothesized that the enhanced incoming CXCL and CCL signaling flows might contribute to the infiltration of CCR2^+^, CCR5^+^, and CXCR6^+^ T cells. Consequently, we investigated the features of *Ccr2* ^+^, *Ccr5* ^+^, and *Cxcr6* ^+^ T cells following YM101 treatment based on the scRNA‐seq data. Based on *Ccr2* mRNA levels, T cells were divided into two groups: *Ccr2^+^* and *Ccr2^−^* (*Ccr2* read count: 0) T cells. In total 692 T cells of the YM101 group, 495 were *Ccr2* ^−^ (71.5%), and 197 were *Ccr2* ^+^ (28.5%) T cells (**Figure** **4**a). Contrary to cytotoxic CD8^+^ T cells, effector‐memory CD8^+^ T cells and early‐activated CD8^+^ T cells were enriched in *Ccr2^+^* subtype (Figure 4b) (Figure S15a, Supporting Information). Within multiple T‐cell subpopulations, particularly cytotoxic CD8^+^ T cells, *Ccr2* expression was associated with decreased levels of genes encoding degranulation (*Nkg7*) and cell‐killing activities (*Prf1*, *Fasl*, and *Ifng*) (Figure 4c). KEGG enrichment results indicated that *Ccr2* expression was linked to lower enrichment scores for TCR, immune cytotoxicity, DNA replication, JAK‐STAT, and mTOR pathways (Figure 4d). Additionally, GSEA data revealed that *Ccr2* expression might inhibit TCR signaling, cell killing, and immune response‐mediated programmed cell death (PCD) (Figure 4e,f).

Similarly, T cells could be classified into *Ccr5^+^* and *Ccr5^−^* (*Ccr5* read count: 0) subsets (Figure 4g). Effector‐memory CD8^+^ T cells were preferentially distributed in *Ccr5^+^* T‐cell subset, while Tregs were enriched in *Ccr5^−^* T‐cell subset (Figure 4h) (Figure S15b, Supporting Information). Further analyses indicated that *Ccr5* expression was correlated with higher expression of genes encoding immunoinhibitory molecules (*Arg2*, *Il18bp*, *Lgals3*, *Cd274*, and *Havcr2*) (Figure 4i). KEGG enrichment analysis suggested that *Ccr5* expression reduced the enrichment scores of TCR, immune cytotoxicity, MAPK, ERBB, JAK‐STAT, and RLR pathways (Figure 4j). GSEA data indicated that *Ccr5* expression could dampen T cell activation and their cytotoxic functions (Figure 4k,l). These results suggested that *Ccr5* ^+^ T cells might act as an immunosuppressive factor in the TME.

Conversely, T cells could be divided into two groups (*Cxcr6* ^+^ *and Cxcr6* ^−^) based on the median *Cxcr6* mRNA level (Figure 4m). Cytotoxic CD8^+^ T cells showed a strong distribution preference in the *Cxcr6* ^+^ T‐cell subset, whereas Tregs and effector‐memory CD8^+^ T cells were enriched in the *Cxcr6* ^−^ T‐cell subset (Figure 4n) (Figure S15c, Supporting Information). Notably, the *Cxcr6* ^+^ T‐cell subset exhibited higher levels of genes encoding degranulation (*Nkg7*), cell‐killing activities (*Ifng, Tnf*, *Fasl*, *Prf1*, and *Gzmb*), and chemokines (*Ccl3*) (Figure 4o). GSEA results indicated that increased *Cxcr6* expression was associated with enhanced immune cytotoxicity, cell killing, and TCR signaling (Figure 4p,q). In summary, *Cxcr6* ^+^ T cells appeared to be immunostimulatory components contributing to the immune renaissance induced by YM101. Importantly, *Ccr5* and *Cxcr6* expression levels were significantly increased following YM101 administration, while *Ccr2* expression was decreased (Figure 4r). Moreover, *Ccr5* ^+^ T cells and *Cxcr6* ^+^ T cells were specifically detected in the YM101 group. Conversely, the specificity of *Ccr2* ^+^ T‐cell distribution in different treatment groups was not observed (Figure 4s). Therefore, our focus in the following study was mainly on *Ccr5* ^+^ T cells and *Cxcr6* ^+^ T cells.

**Figure 5:**
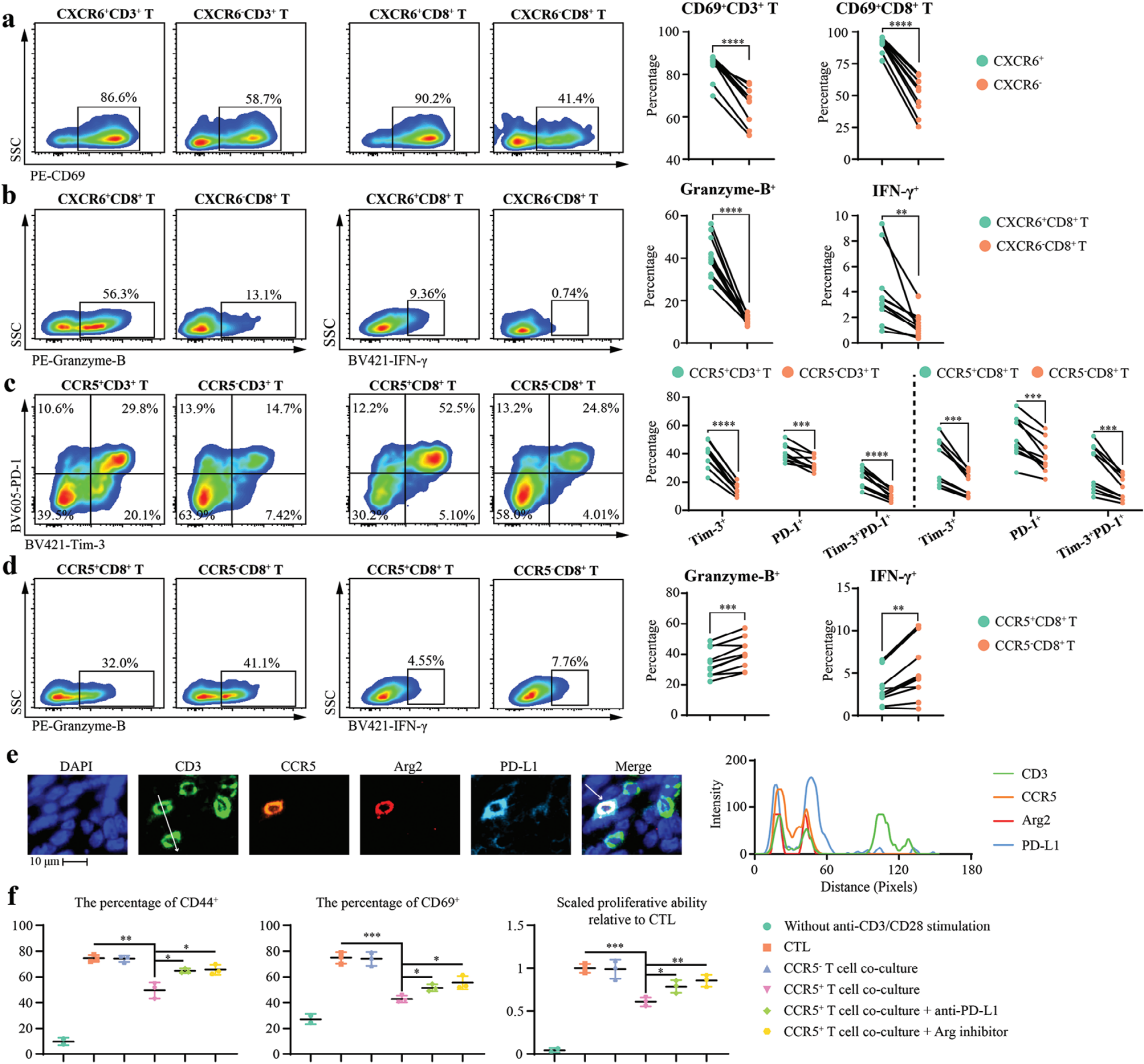
The opposite functions of CXCR6^+^ and CCR5^+^ T cells. a–d) Representative images and quantitative analysis of CD69^+^CXCR6^+^ T cells, Granzyme‐B^+^CXCR6^+^CD8^+^ T cells, IFN‐γ^+^CXCR6^+^CD8^+^ T cells, Tim‐3^+^PD‐1^+^CCR5^+^ T cells, Granzyme‐B^+^CCR5^+^CD8^+^ T cells, and IFN‐γ^+^ CCR5^+^CD8^+^ T cells in flow cytometry assays (n = 10). e) Representative images and co‐localization analysis of immunofluorescent staining showing the expression of Arg2 and PD‐L1 in CCR5^+^ T cells. Immunofluorescent staining was performed on YM101‐treated EMT‐6 tumor samples. The histogram on the right represents the signal intensities along the white line indicated in the CD3 image. f) Flow cytometry assays to measure the influences of tumor‐infiltrating CCR5^+^ T cells on the activation of naïve T cells under the stimulation of anti‐CD3/CD28 (n = 2 for negative control without stimulation, n = 3 for others). Quantitative analyses of CFSE dilution, along with CFSE‐labeled CD69^+^ and CD44^+^ T cells, indicating T cell activation. Statistical analyses were performed using paired Student's *t*‐test (a–d) and Student's *t*‐test (f). Significance is indicated as: ^*^ *p* < 0.05, ^**^ *p* < 0.01, ^***^ *p* < 0.001, ^****^ *p* < 0.0001.

Subsequently, we evaluated the immune states of CXCR6^+^ and CCR5^+^ T cells in YM101‐treated tumor tissues using flow cytometry (Figure S16a,b, Supporting Information). Our flow cytometry data demonstrated increased T‐cell activation marker CD69 expression in CXCR6^+^CD3^+^ and CXCR6^+^CD8^+^ T cells (**Figure** **5**a). Additionally, CXCR6^+^CD8^+^ T cells exhibited elevated levels of cytotoxicity‐associated cytokines, such as Granzyme‐B and IFN‐γ (Figure 5b). In contrast, CCR5^+^ T cells presented a state of immune exhaustion and compromised cytotoxic potential. The proportions of Tim‐3^+^, PD‐1^+^, and Tim‐3^+^PD‐1^+^ cells were heightened in CCR5^+^CD3^+^ and CCR5^+^CD8^+^ T cells (Figure 5c). Furthermore, a significant reduction in Granzyme‐B and IFN‐γ was observed in CCR5^+^CD8^+^ T cells (Figure 5d). Immunofluorescent (IF) staining assays confirmed the co‐expression of immunoinhibitory molecules such as PD‐L1 and Arg2 in CCR5^+^ T cells (Figure 5e). Interestingly, flow cytometry data indicated that the expression of CCR5 and CXCR6 in T cells is largely mutually exclusive, with low CXCR6 expression within CCR5⁺ T cells and vice versa (Figure S16c,d, Supporting Information).

Subsequently, we confirmed that the activation of naïve T cells was inhibited in the presence of CCR5^+^ T cells. Specifically, when naïve T cells were co‐cultured with CCR5^+^ T cells, there was a notable reduction in their proliferation and expression levels of activation markers CD44 and CD69. This impairment in T cell activation was partially reversible upon the introduction of anti‐PD‐L1 antibody and Arginase inhibitor, indicating the potential pathways through which CCR5^+^ T cells exert their suppressive effects (Figure 5f). In summary, our findings substantiate the enhanced functions of CXCR6^+^ T cells, while underscoring CCR5^+^ T cells as an immunosuppressive subset characterized by diminished cytokine production and enhanced immunomodulation capabilities.

### 2.6 Blocking CCR5^+^ T Cell Accumulation Improves YM101 Efficacy

**Figure 6:**
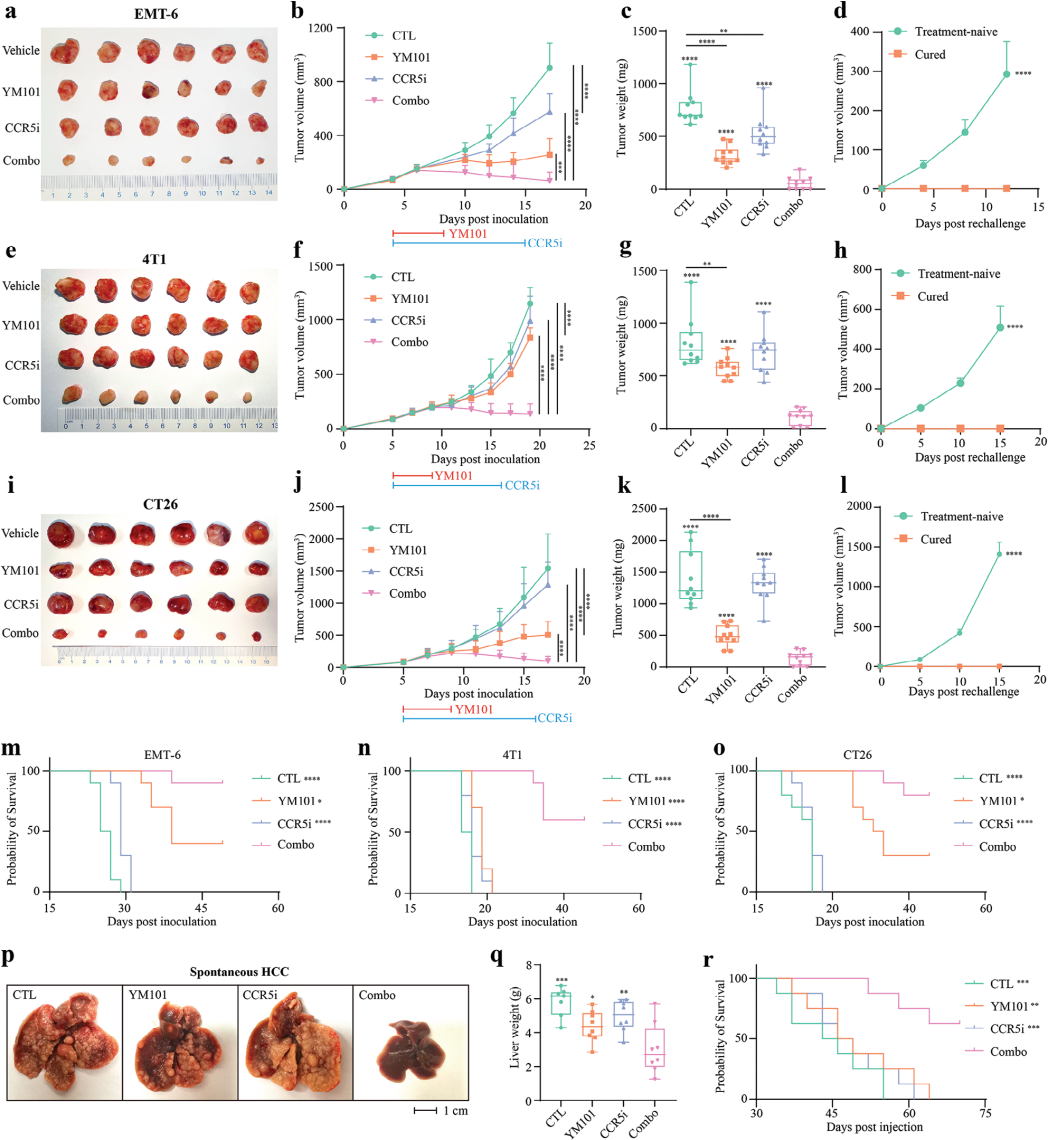
The synergistic antitumor effect between YM101 and CCR5 blockade. All mice were randomly assigned to one of four groups, namely the control, Maraviroc (CCR5i), YM101, and Maraviroc combined with YM101. Treatment was started when tumor volumes reached 50 mm^3^. Maraviroc (50 mg kg^−1^) was administered daily by intraperitoneal injection. For antibody treatments, tumor‐bearing mice received equimolar quantities of hIgG (6.6 mg kg^−1^) or YM101 (9 mg kg^−1^) every two days via intraperitoneal injection. The blue line indicates Maraviroc treatment, while the red line represents antibody treatment. Mice were euthanized when tumor volumes reached 2000 mm^3^ or at the end of experiments. a–c) Efficacy evaluation in the murine breast cancer EMT‐6 model (n = 10). d) Rechallenge assay in the EMT‐6 model (n = 4). e–g) Efficacy evaluation in the murine breast cancer 4T1 model (n = 10). h) Rechallenge assay in the 4T1 model (n = 2). i–k) Efficacy evaluation in the murine colon cancer CT26 model (n = 10). l) Rechallenge assay in the CT26 model (n = 2). m–o) The overall survival curves of EMT‐6, 4T1, and CT26 models (n = 10). p,q) Tumor burden of spontaneous hepatocellular carcinoma (HCC) model. Surviving tumor‐bearing mice were euthanized, and their livers were harvested 35 days after plasmid injection. The representative images of mouse livers were shown (n = 8; note: one mouse in the CTL group died before liver collection at the end of the experiment). r) The overall survival curves of spontaneous HCC model (n = 8). Statistical analyses were conducted using Student's *t*‐test (except for survival analysis) and Log‐rank test (for survival analysis). ^*^ *p* < 0.05, ^**^ *p* < 0.01, ^***^ *p* < 0.001, ^****^ *p* < 0.0001 indicate significant differences compared to the Maraviroc combined with YM101 group.

We hypothesized that YM101 effectively promoted the infiltration and recruitment of various T cells, including CCR5^+^ T cells and CXCR6^+^ T cells. While the latter exhibited immunostimulatory activities contributing to the superior efficacy of YM101, the former acted as an immunoinhibitory factor, undermining antitumor responses. To validate our hypothesis that blocking CCR5^+^ T cell accumulation improves YM101 efficacy, we evaluated the antitumor effects of CCR5 inhibitor (CCR5i) Maraviroc in combination with YM101 in multiple murine tumor models. In EMT‐6, 4T1, and CT26 models, Maraviroc effectively synergized with YM101 to suppress tumor growth and reduce tumor burden (**Figure** **6**a–l). Notably, the 4T1 model was previously regarded as having a poor response to YM101. Encouragingly, we found that Maraviroc effectively improved YM101 performance in the 4T1 model.

Moreover, tumor‐bearing mice that had received the combination therapy and exhibited complete tumor regression were rechallenged with the same tumor cell line. In the combination treatment groups of these tumor models, four out of ten (EMT‐6), two out of ten (4T1), and two out of ten (CT26) mice achieved complete remission. These cured mice were then used for the tumor rechallenge. Remarkably, these mice displayed complete resistance to tumor rechallenge, indicating the induction of durable antitumor immunity by combination therapy (Figure 6d,h,l). These findings suggest that combination therapy not only effectively eliminates established tumors but also induces long‐lasting immune memory, protecting against tumor recurrence. Besides, in EMT‐6, 4T1, and CT26 models, the combination treatment significantly prolonged the survival of tumor‐bearing mice (Figure 6m–o) without significant toxic effects or weight loss (Figure S17a–c, Supporting Information). Apart from cell‐line‐derived tumor models, the combination therapy retarded the tumor development and extended survival in the AKT/Ras‐driven spontaneous hepatocellular carcinoma (HCC) model (Figure 6p–r) (Figure S17d, Supporting Information).

### 2.7 Mechanisms Underlying Combination Therapy Efficacy

**Figure 7:**
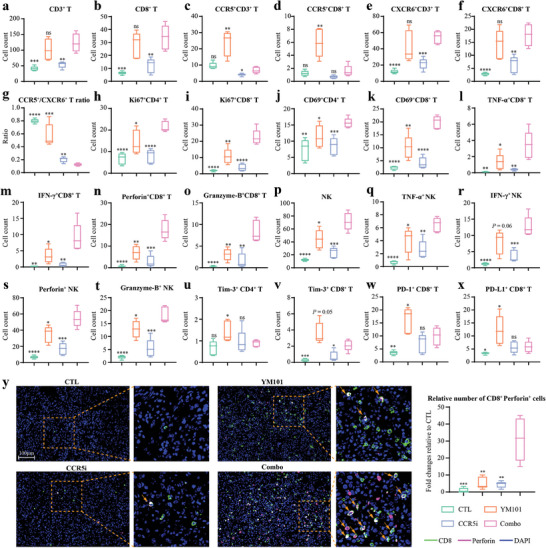
Therapy combining Maraviroc with YM101 enhanced the activities of tumor‐infiltrating lymphocytes in the EMT‐6 model. a–x) Quantitative analysis in the flow cytometry assays included CD3^+^ T cells, CD8^+^ T cells, CCR5^+^ T cells, CXCR6^+^ T cells, CCR5^+^ to CXCR6^+^ T ratio, proliferating T cells (Ki67^+^ T cells), early‐activated T cells (CD69^+^ T cells), cytotoxic CD8^+^ T cells (TNF‐α^+^, IFN‐γ^+^, Perforin^+^, and Granzyme‐B^+^), NK cells, activated NK cells (TNF‐α^+^, IFN‐γ^+^, Perforin^+^, and Granzyme‐B^+^), Tim‐3^+^ PD‐1^+^ T cells, and immunosuppressive PD‐L1^+^ CD8^+^ T cells. The number of immune cells per 100 mg of EMT‐6 tissue was determined and compared (n = 5). y) Representative images of immunofluorescent staining showing Perforin^+^CD8^+^ T‐cell infiltration in tumors (n = 5). Statistical analyses were conducted using Student's *t*‐test. ^*^ *p* < 0.05, ^**^ *p* < 0.01, ^***^ *p* < 0.001, ^****^ *p* < 0.0001 indicate significant differences compared to the Maraviroc combined with YM101 group.

To dissect the mechanisms underlying the enhanced therapeutic effects of combination therapy, we explored the activation state of TILs within the TME. Flow cytometry assays were conducted to investigate the changes in the TME following combination therapy (Figure S18, Supporting Information). EMT‐6 tumor tissues were harvested to quantify the numbers of multiple tumor‐infiltrating immune cells. Although both YM101 monotherapy and the combination of YM101 with Maraviroc substantially expanded TILs, Maraviroc did not result in additional increases in T‐cell infiltration (**Figure** **7**a,b). However, the combination therapy effectively mitigated YM101‐mediated CCR5^+^ T‐cell accumulation, with modest effects on CXCR6^+^ T‐cell infiltration (Figure 7c–f). Consequently, the combination therapy relatively expanded CXCR6^+^ T cells and upregulated the ratio of CXCR6^+^ to CCR5^+^ T cells (Figure 7g). Next, we assessed the activity, proliferation, and cytotoxic functions of TILs among the four groups. Flow cytometry data demonstrated that the combination therapy significantly increased the numbers of proliferating T cells (Ki67^+^ T cells), early‐activated T cells (CD69^+^ T cells), and cytotoxic CD8^+^ T cells (TNF‐α^+^, IFN‐γ^+^, Perforin^+^, and Granzyme‐B^+^ CD8^+^ T cells) (Figure 7h–o). Additionally, the combination treatment elevated the numbers and cytotoxicity activity of NK cells (Figure 7p–t). Moreover, compared to YM101 alone, the combination treatment decreased the numbers of exhausted T cells (Tim‐3^+^ and PD‐1^+^ T cells) and immunosuppressive PD‐L1^+^ T cells (Figure 7u–x). Also, our earlier investigations have substantiated that YM101 can efficiently enhance T‐cell infiltration and overcome resistance to immunotherapy by reducing peritumoral collagen deposition.^[^ ^42^ ^]^ In alignment with these prior observations, we observed a significant suppression of peritumoral collagen production with YM101, both alone and in combination with Maraviroc (Figure S19, Supporting Information). Besides, IF assays indicated that the combination therapy substantially increased Perforin^+^CD8^+^ T cells in the TME. Relative to YM101 monotherapy, additional Maraviroc not only expanded tumor‐infiltrating CD8^+^ T cells but also conferred upon these CD8^+^ T cells the enhanced cytotoxic potential (Figure 7y).

**Figure 8:**
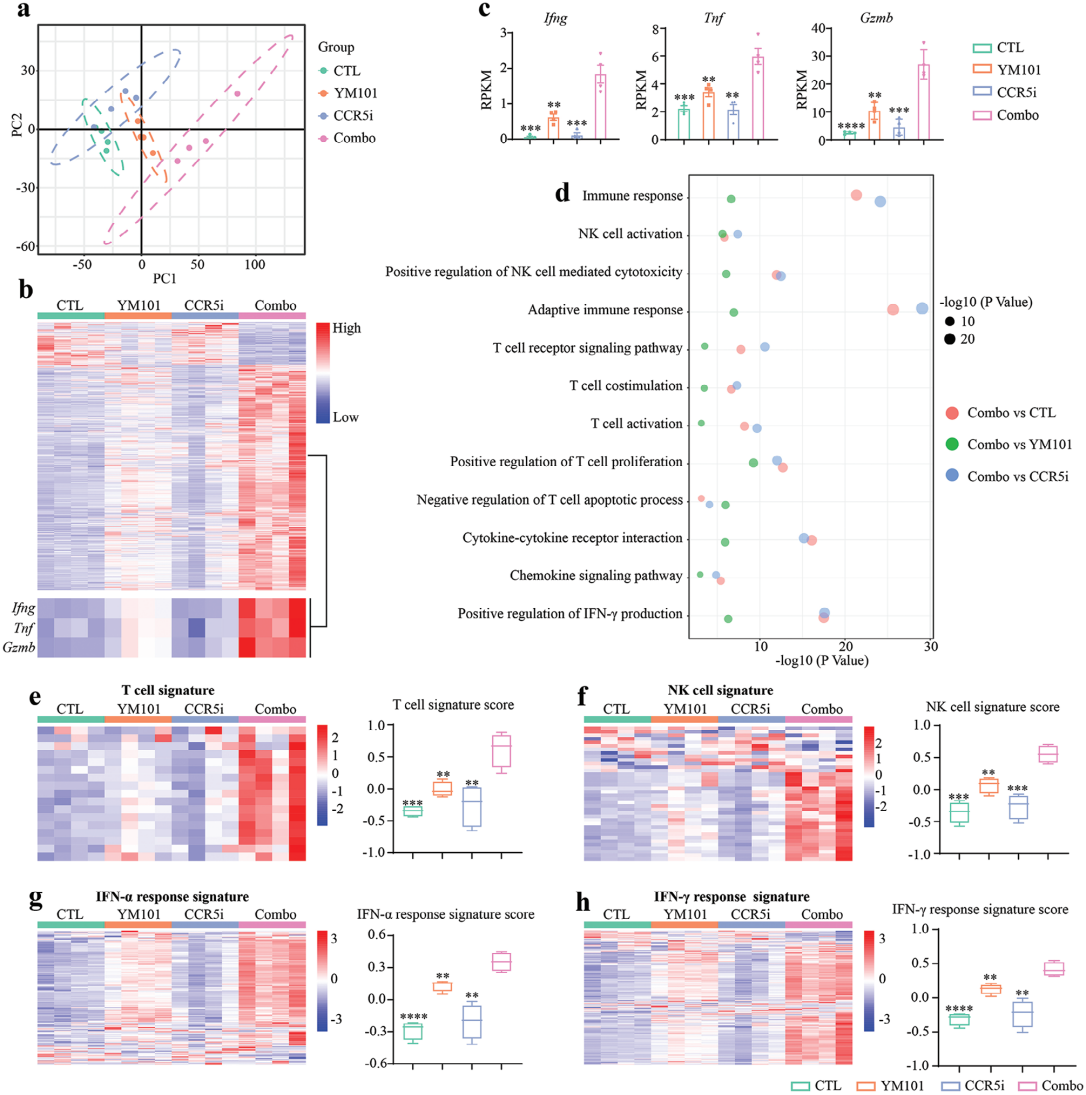
Combination therapy with Maraviroc and YM101 induced alterations in the TME and boosted the expression of antitumor immunity‐related genes in the EMT‐6 model. a) The principal component analysis shows the similarities of different groups. b,c) The heatmap displays differentially expressed genes among the four groups, with a focus on immune‐killing genes such as *Ifng*, *Tnf*, and *Gzmb*, which were quantitatively assessed (n = 4). d) Gene Ontology (GO) and KEGG enrichment analysis highlighting significantly enriched biological processes and signaling in the Maraviroc combined with the YM101 group. e–h) Scores of immune signatures, presented as heatmaps showing the scaled expression levels of genes composing immune signatures. Statistical analyses were performed using Student's *t*‐test (c,e–h). ^*^ *p* < 0.05, ^**^ *p* < 0.01, ^***^ *p* < 0.001, ^****^ *p* < 0.0001 indicate significant differences compared to the Maraviroc combined with YM101 group.

Subsequently, we conducted bulk RNA‐seq assays to comprehensively assess the impact of combination therapy on the TME. Principal component analysis (PCA) results revealed that the combination therapy group exhibited a distinct expression profile, partially resembling YM101 but significantly differing from the other two groups (**Figure** **8**a). Differentially expressed gene (DEG) analysis identified 1766, 176, and 1197 genes with significantly upregulated or downregulated expression in the CTL, YM101, and CCR5i groups, when compared to the combination therapy. Among these DEGs, genes encoding immune cytotoxicity‐associated molecules such as *Prf1* (Perforin), *Ifng* (IFN‐γ), and *Gzmb* (Granzyme‐B) were upregulated in the combination therapy (Figure 8b,c). Subsequently, we performed enrichment analysis to annotate the biological functions of these DEGs. The results demonstrated significant enrichment of pathways related to immune response, NK cell activation, adaptive immune response, T‐cell receptor signaling, T‐cell co‐stimulation, T‐cell activation, negative regulation of T‐cell apoptosis, positive regulation of T‐cell proliferation, cytokine and chemokine signaling, and IFN‐γ production in the combination therapy (Figure 8d). Furthermore, we quantitatively assessed the effects of the combination therapy using four immune signatures, including T cells, NK cells, IFN‐α response, and IFN‐γ response. Our data illustrated that the combination therapy effectively increased the corresponding immune scores, indicating a systematic enhancement in multiple aspects of the cancer‐immunity cycle (Figure 8e–h).

Given the reported capacity of CCR5i to stimulate antitumor immune responses by modulating tumor‐infiltrating myeloid cells,^[^ ^45^ ^]^ we proceeded to evaluate the quantity and activity of myeloid cells using flow cytometry assays. In contrast to the pronounced impact of YM101 on myeloid cells, the influence of Maraviroc was comparatively modest. Within this model, the Maraviroc administration resulted in only a slight reduction in MDSC numbers. Consequently, the addition of Maraviroc to YM101 therapy did not significantly increase DC quantity, reduce MDSC accumulation, or alter macrophage polarization (Figure S20, Supporting Information). To further verify whether the synergistic effect of YM101 and Maraviroc is achieved through the modulation of immunosuppressive cells like MDSCs, we specifically depleted macrophages, MDSCs, and Tregs in the EMT‐6 tumor model using CLD‐Lp, anti‐DR5, and anti‐CD25. The effectiveness of depletion was confirmed through flow cytometric analysis of tumor tissues. Across these EMT‐6 models with the depletion of specific immune cell populations, Maraviroc was found to still enhance the therapeutic effect of YM101, suggesting that the synergy between YM101 and Maraviroc might be realized through bypass mechanisms other than the direct targeting of these immunosuppressive components (Figure S21, Supporting Information). Considering the potent efficacy of YM101 in clearing immunosuppressive MDSCs and macrophages, and the relatively modest regulatory effect of Maraviroc on myeloid cells, it is unlikely that Maraviroc enhances the therapeutic effects of YM101 by targeting these cells. Hence, based on the results of in vivo models and scRNA‐seq, we hypothesize that CCR5^+^ T cells may be the primary targets through which Maraviroc exerts synergistic effects with YM101.

**Figure 9:**
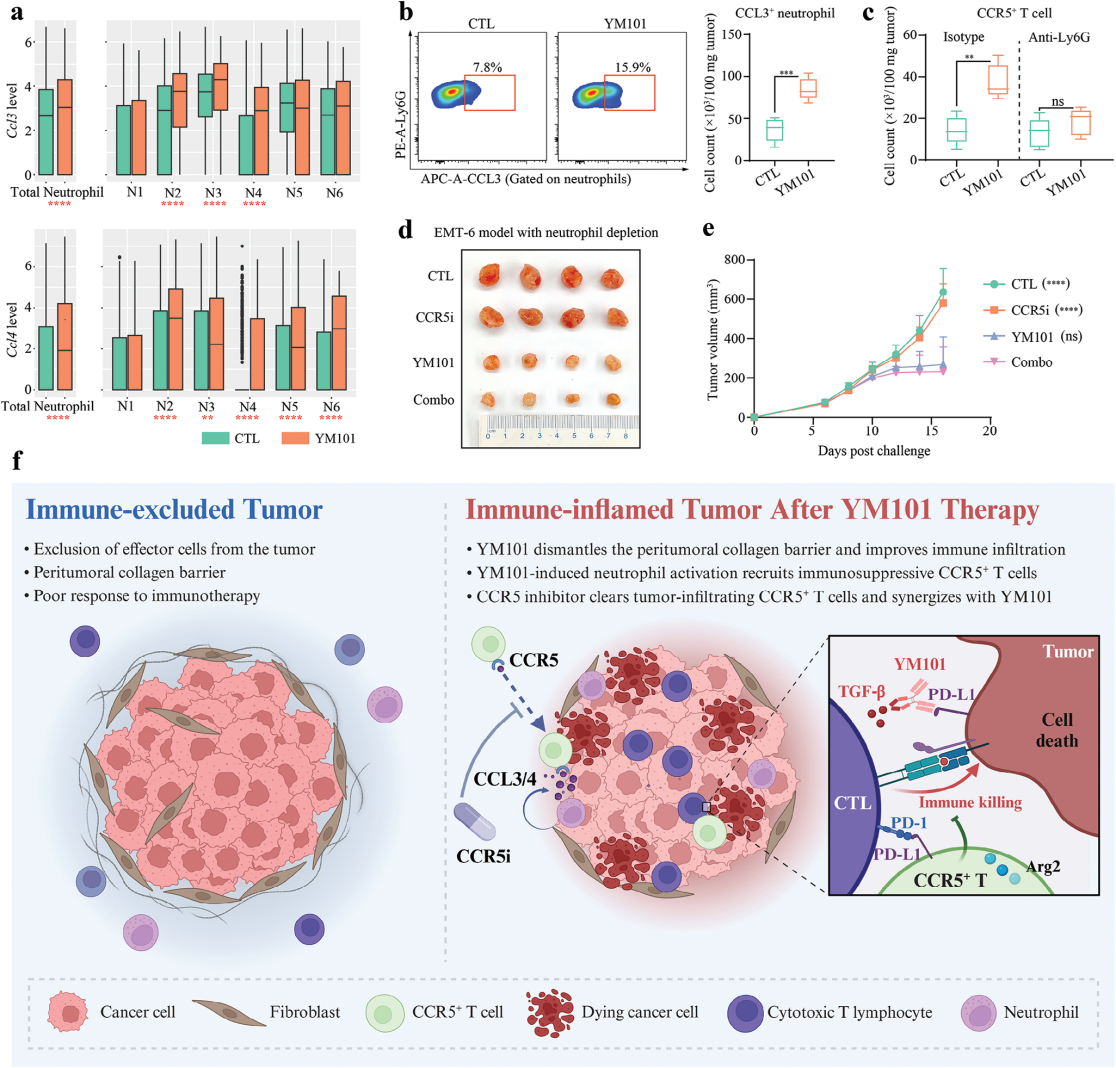
Mechanisms underlying the synergistic effect of CCR5 antagonist and YM101. a) Comparative boxplot analysis depicting the expression levels of *Ccl3* and *Ccl4* in the total neutrophil population and within specific neutrophil subsets in CTL and YM101‐treated groups. b) Flow cytometry results showing the impact of YM101 treatment on CCL3 expression in neutrophils (n = 5). c) Flow cytometry analysis evaluating the influences of neutrophil depletion on the presence of CCR5^+^ T cells in the TME following YM101 administration (n = 5). d,e) Establishing the EMT‐6 tumor model to investigate the influence of neutrophil depletion on the synergistic efficacy of CCR5 antagonist and YM101 (n = 8). Statistical analyses were conducted using Student's *t*‐test. ^**^ *p* < 0.01, ^***^ *p* < 0.001, ^****^ *p* < 0.0001 indicate significant differences. f) Schematic diagram showing the synergistic effect. TGF‐β plays a pivotal role in cancer immune evasion and resistance to immunotherapy by inhibiting the functions of various immune cells and fostering the generation of peritumoral collagen. This physical barrier surrounding the tumor impedes immune cell penetration into the tumor microenvironment (TME), culminating in the formation of immune‐excluded tumors. The anti‐TGF‐β/PD‐L1 bispecific antibody YM101 has demonstrated efficacy in dismantling the peritumoral collagen barrier, facilitating immune infiltration, and partially overcoming immunotherapy resistance. However, YM101 also activates neutrophils within the TME, increasing the expression of CCR5 ligands such as CCL3 and CCL4. This upregulation drives the chemotaxis of immunosuppressive CCR5^+^ T cells into the TME. These cells, highly expressing immunosuppressive markers like PD‐L1, and Arg2, infiltrate the TME following the collagen barrier disruption, eventually undermining the immunotherapy efficacy. To surmount this challenge, we devised a strategy by combining YM101 with Maraviroc, a CCR5 antagonist. This synergistic approach not only curtailed the accumulation of CCR5^+^ T cells with immunosuppressive traits but also fine‐tuned the immune response orchestrated by YM101.

Analysis of the intercellular communication network suggested that the communication between CCR5^+^ T cells and myeloid cells, especially neutrophils, was significantly enhanced following YM101 treatment. We speculate that neutrophils mainly mediate the post‐treatment accumulation of CCR5^+^ T cells. Based on the scRNA‐seq data analysis, YM101 significantly increased the expression levels of *Ccl3* and *Ccl4* in total neutrophils, with notable *Ccl3* upregulation in the N2‐*Cxcl10*, N3‐*Cxcl3*, and N4‐*Mmp9* subsets and *Ccl4* increase in five subsets excluding N1‐*Retnlg* (**Figure** **9**a). Additionally, flow cytometry analysis of EMT‐6 tissues treated with YM101 showed a significant increase in CCL3^+^ neutrophil number (Figure 9b). Furthermore, neutrophils were specifically depleted in mice using an anti‐Ly6G antibody, with the depletion verified by flow cytometry. The results showed that the number of CCR5^+^ T cells in the TME decreased with the specific depletion of neutrophils, and the Maraviroc‐enhanced antitumor activity of YM101 was abolished (Figure 9c–e). Previous studies reported the activation of neutrophils following immunotherapy.^[^ ^46^, ^47^ ^]^ Consistently, YM101 therapy promoted neutrophil activation, which highly expressed chemokines such as CCL3 and CCL4. Therefore, we conclude that YM101‐induced neutrophil activation recruits immunosuppressive CCR5^+^ T cells via CCR5 ligand secretion, creating a feedback loop that diminishes the antitumor response. Using a CCR5 inhibitor like Maraviroc can counteract this feedback, boosting the effect of YM101 (Figure 9f).

## 3 Discussion

In this study, we observed the superior antitumor potential of anti‐TGF‐β/PD‐L1 BsAb YM101 in murine tumor models. Although the efficacy was better than that of anti‐PD‐1, YM101 therapy did not achieve complete tumor regression in all tumor‐bearing mice. As we previously reported, the efficacy of YM101 was limited in multiple tumor models. Besides TGF‐β and PD‐1 signaling pathways, other immunoinhibitory factors in the TME also participate in cancer immune evasion and contribute to immunotherapy resistance.^[^ ^48^, ^49^ ^]^ Therefore, exploring changes in the TME composition is meaningful to maximize YM101 efficacy and reduce the risk of treatment resistance.

Here, our scRNA‐seq data unveiled the intricate landscape of the TME and provided insights into the cellular and molecular changes induced by YM101. The analysis categorized cells into distinct clusters representing various immune cell lineages, including macrophages, neutrophils, monocytes, NK cells, T cells, cDCs, pDCs, and tumor cells. Notably, YM101 treatment significantly altered the composition of the TME by expanding the ratios of immune cell populations associated with antitumor immunity, such as T cells, NK cells, pDCs, cDCs, and M1‐like macrophages, while reducing immunosuppressive M2‐like macrophages. Furthermore, YM101 treatment strengthened pathways related to inflammatory responses, antigen presentation, cell‐killing, and innate immune sensing within the TME. In contrast, the enrichment of pathways associated with immunosuppression such as TGF‐β signaling was notably reduced. Also, the YM101‐treated group exhibited an overall increase in cell communications, including both the number and strength of interactions. These enhanced cell communications were primarily associated with pro‐inflammatory and cell‐killing pathways. Conversely, YM101 reduced intercellular interactions within IL‐4 and TGF‐β signaling pathways. Downstream analyses within T cells showed that increased cell communications within the CCL and CXCL pathways were unique to the YM101 group, particularly with myeloid cells. Generally, these scRNA‐seq findings provided valuable insights into the mechanisms underlying the antitumor effects of YM101, highlighting its ability to reshape the TME and promote a pro‐inflammatory and immunostimulatory microenvironment conducive to antitumor immune responses.

Further investigation revealed that YM101‐induced enhancement of T‐cell communications largely depended on CCR5 and CXCR6 signaling. Importantly, *Ccr5* ^+^ and *Cxcr6* ^+^ T cells were significantly expanded following YM101 treatment, indicating the potential immunomodulatory effects of YM101 on these T cell subsets. *Cxcr6* ^+^ T cells in the YM101 group exhibited enhanced cytotoxicity and immune activation, suggesting their immunostimulatory roles. In contrast, *Ccr5* ^+^ T cells possessed hampered TCR signaling but strengthened immunomodulatory effects. *Arg2*, *Il18bp*, *Lgals3*, and *Cd274* (encoding Arg2, IL‐18BP, galectin‐3, and PD‐L1, respectively) are well‐established immunosuppressive factors,^[^ ^50^, ^51^, ^52^ ^]^ which were significantly increased in *Ccr5* ^+^ T cells. Considering that CCR5 and CXCR6 signaling are responsible primarily for T‐cell chemotaxis and recruitment, it is logical to assume that YM101 promotes the recruitment and infiltration of CCR5^+^ and CXCR6^+^ T cells. However, CCR5^+^ T cells within the TME exhibit immunosuppressive characteristics, potentially acting as the Trojan horse that hinders the efficacy of antitumor immune responses and limits the therapeutic effects of YM101.

Notably, our scRNA‐seq data have uncovered that CCR5^+^ T cells constitute a diverse and multifaceted group, encompassing Tregs, cytotoxic CD8^+^ T cells, and both effector and central memory T cells. This diversity implies that CCR5^+^ T cells can assume varied roles across different pathologies and biological scenarios. For instance, CCR5 signaling has been reported to be involved in T cell chemotaxis, activation, and even apoptosis, highlighting its multifunctional features.^[^ ^53^, ^54^ ^]^ In addition, CCR5^+^ Tregs have been identified to exhibit more pronounced immunosuppressive capabilities than their CCR5^−^ counterparts.^[^ ^55^, ^56^ ^]^ Furthermore, our analysis revealed a notable increase in the proportion of effector memory CD8^+^ T cells among total CCR5^+^ T cells. This finding aligns with recent studies that have identified subsets of memory‐like CD8^+^ T cells demonstrating Treg phenotypes and functions.^[^ ^57^ ^]^ We speculate that this heterogeneous group of CCR5^+^ T cells contributes to immunosuppression within the tumor milieu. Therefore, blocking CCR5^+^ T‐cell accumulation in the TME might be feasible to further improve YM101 performance.

In murine tumor models, Maraviroc combined with YM101 suppressed the accumulation of CCR5^+^ T cells and increased the CXCR6^+^ T cell population. This shift in the balance of T cell subsets contributed to the improved therapeutic outcomes observed with combination therapy. Importantly, the combination therapy induced durable antitumor immunity, as demonstrated by the resistance to tumor rechallenge in mice that had achieved complete tumor regression. These findings underscore the significance of selectively targeting CCR5 to optimize the therapeutic potential of immunotherapies like YM101. By removing the immunosuppressive component, combination therapy not only enhanced immediate treatment outcomes but also established long‐lasting immune memory, protecting against tumor recurrence.

CCR5 is commonly regarded as a core coreceptor for the entry of human immunodeficiency virus (HIV), and CCR5 antagonists such as Maraviroc have been widely used for HIV treatment.^[^ ^58^ ^]^ However, the role of CCR5 signaling in cancer is still controversial. On the on hand, accumulated evidence demonstrated that hyperactive CCR5 signaling could be hijacked to support tumor progression. CCR5 signaling endows tumor cells with enhanced capabilities of invasion, DNA damage repair, stemness, angiopoiesis, metabolic reprogramming, and immune evasion.^[^ ^59^, ^60^, ^61^, ^62^, ^63^, ^64^ ^]^ In previous studies, CCR5 signaling causes cancer immune escape mainly by recruiting immunosuppressive cells such as MDSCs, M2‐like macrophages, CCR5^+^ Tregs, and CAFs.^[^ ^45^, ^65^, ^66^ ^]^ On the other hand, CCR5 has been identified as a T‐cell‐intrinsic marker of immune checkpoint inhibitor sensitivity.^[^ ^67^ ^]^ In this study, we observed a significant increase in CCR5^+^ T cell accumulation within the TME following YM101 treatment, characterized by diminished T cell activity yet heightened immunosuppressive capacity. While this observation might seem to contradict previous findings, it complements them upon closer examination. Our results show that YM101 remodels the immune microenvironment, inhibits tumor growth, and simultaneously promotes CCR5^+^ T cell accumulation. This enhanced presence of CCR5^+^ T cells is associated with partial tumor regression and a more immunosupportive TME post‐immunotherapy, aligning with earlier research and indicating no discrepancy.

However, we interpret the increased infiltration of CCR5^+^ T cells not as a direct contributor to tumor immunity, but rather as a negative feedback mechanism after immune activation. In our study, the accumulation of CCR5^+^ T cells within the tumor setting emerged as a secondary effect, in turn dampening the anticipated boost in tumor immunity following YM101 therapy. Blocking the recruitment of these sub‐optimally activated T cells further enhances the therapeutic effect of YM101. This suggests that the selective modulation of CCR5 signaling may be a valuable strategy in cancer immunotherapy, provided that the complex interplay between immune cell subsets is carefully considered. Collectively, this concept of combination therapy, as demonstrated in our study with YM101 and Maraviroc, could extend to a broader range of immunotherapies and represents a promising avenue for future research in the field of cancer immunotherapy.

## 4 Conclusion 

In this study, we observed the superior antitumor potential of the anti‐TGF‐β/PD‐L1 BsAb YM101 and explored the intricate landscape of the TME after YM101 treatment by scRNA‐seq analysis. The results confirmed the capacity of YM101 to create a pro‐inflammatory and immunostimulatory TME conducive to antitumor immune responses. Moreover, YM101 treatment significantly expanded CCR5^+^ and CXCR6^+^ T cell populations, with distinct functional consequences. CXCR6^+^ T cells exhibited enhanced cytotoxicity and immune activation, whereas CCR5^+^ T cells displayed immunosuppressive characteristics. Blocking the recruitment of CCR5^+^ T cells, which could potentially act as a Trojan horse hindering antitumor immune responses, emerged as a promising strategy to further optimize YM101 therapy.

## 5 Experimental Section

### Cell Line and Agents

Murine tumor cell lines, namely EMT‐6 (breast cancer), 4T1 (breast cancer), and CT26 (colon cancer), were cultured in RPMI‐1640 medium supplemented with 10% fetal bovine serum. Therapeutic agents, including the Isotype antibody (hIgG), PD‐L1 blocking antibody, TGF‐β neutralizing antibody, and anti‐TGF‐β/PD‐L1 BsAb YM101, were sourced from Wuhan YZY Biopharma. The CCR5 antagonist Maraviroc (UK‐427857) was acquired from MCE corporation.

### Murine Tumor Models

For two orthotopic breast cancer models EMT‐6 and 4T1, BALB/c mice received inoculations of 5 × 10^5^ cells in the right mammary fat pad. For the subcutaneous colon cancer model CT26, BALB/c mice were inoculated with 1 × 10^6^ CT26 cells in the right groin. The antitumor efficacy of YM101 was validated in EMT‐6 and CT26 models. Mice were randomized into distinct treatment groups when tumor volumes reached 100 mm^3^, with seven mice per group. Treatment commenced at this point and continued for six doses. For antibody treatments, tumor‐bearing mice received equimolar quantities of hIgG (6.6 mg kg^−1^), PD‐L1 blocking antibody (6.6 mg kg^−1^), TGF‐β neutralizing antibody (6.6 mg kg^−1^), or YM101 (9 mg kg^−1^) every 2 days via intraperitoneal injection. Tumor volume was measured using the formula: 0.5 × length × width^2^. Survival was monitored for 7 weeks post‐inoculation, with mice euthanized when tumor volumes reached 2000 mm^3^ or at the end of in vivo experiments.

Moreover, the efficacy of YM101 combined with Maraviroc was evaluated in EMT‐6, 4T1, and CT26 models (ten mice per group). Treatment (including CTL, YM101, Maraviroc, and the combination) was started when tumor volumes reached 50 mm^3^. Mice received antibody administration three times at the doses mentioned above. Maraviroc (50 mg kg^−1^) was administered daily by intraperitoneal injection.^[^ ^68^ ^]^ Besides, to obtain sufficient tumor tissues for subsequent RNA‐seq and flow cytometry assays, the mouse experiments were repeated and delayed the treatment when tumor volumes reached 300 mm^3^. Furthermore, to mimic the real process of tumor initiation and development in vivo, we established an AKT/Ras‐driven spontaneous murine HCC model through hydrodynamic injection, delivering 5 µg of myr‐AKT1 and 25 µg of NRasV12 plasmids, plus 2 µg of sleeping beauty transposase in saline, into the tail vein of C57BL/6 mice.^[^ ^69^ ^]^ Livers were harvested to evaluate the impact of the treatment on tumor burden. Then, we repeated this spontaneous HCC model for survival analysis, with a total follow‐up period of over 2 months.

The animal operations in this study were evaluated and approved by the Institutional Animal Care and Use Committee of The First Affiliated Hospital, College of Medicine, Zhejiang University (No. 2023–378).

### scRNA‐Seq

The scRNA‐seq project was based on the PRJNA837188 data with updated analysis pipeline.^[^ ^70^ ^]^ In brief, following three doses of YM101 administration, fresh EMT‐6 tissues were collected. Tumor tissues underwent treatment with the dissociation buffer (RPMI‐1640 medium containing 0.5 mg mL^−1^ DNase I, 0.5 mg mL^−1^ Hyaluronidase, and 1 mg mL^−1^ Collagenase B) at 37 °C for 40 min. Subsequently, nanobeads for CD45 positive selection (480028, BioLegend) were utilized to isolate sufficient immune cells (≈80%) for scRNA‐seq. Living cells were then enriched via cell sorting. Cells from two mice within the same group were pooled to form one sample, which was then loaded onto a 10× Genomics Chromium Controller. ScRNA‐seq libraries were generated in accordance with the 10× Genomics Chromium single‐cell 5’ reagent kits v2 protocol. For this study, CTL‐ and YM101‐treated samples (six tumors per group, pooled as three samples per group) were used for scRNA‐seq analysis.

### Raw Data Processing and Analysis for scRNA‐Seq

Using the GRCm39 reference genome, the standard Cell‐ranger analysis pipeline (version 6.1.2) was followed. R software (version 4.3.1) with the *Seurat* package (version 4.3.0.1) was employed for subsequent analyses. Cells with poor quality, defined by more than 10% mitochondrial gene expression, fewer than 200 detected genes, or more than 20 000 detected transcripts, were filtered out, resulting in 45 012 cells for PCA. The top 50 dimensions were utilized for t‐distributed stochastic neighbor embedding (t‐SNE) and uniform manifold approximation and projection (UMAP) reduction. Employing a resolution of 1.5, cells were clustered via the *FindClusters* function of the *Seurat* package. *SingleR* package (version 1.8.0) and common markers were used to annotate a total of 31 clusters. Tumor cell clusters were annotated using the *InferCNV* package (version 1.16.0). Odds ratios (ORs) were used to gauge the preference of specific immune cluster distributions in CTL or YM101‐treated tumor tissues.^[^ ^71^ ^]^ Adopting the thresholds established by Zhang's team, we consider an OR greater than 1.5 as indicative of preferential distribution in a specific tissue type, whereas an OR below 0.5 signifies non‐preferential distribution, commonly equivalent to adjusted *p*‐values far less than 0.0001.

GSEA was used to depict the features of specific immune cell subsets, employing the *singleseqgset* package (version 1.2.9) (Tables S1–S13, Supporting Information). DEGs were identified through the *FindMarkers* function (Log‐fold change more than 0.25, adjusted *p* below 0.05, minimum detection rate: 0.1) within the *Seurat* package. Functional enrichment analysis was conducted using the *ClusterProfiler* package (version 4.8.2), with the significance threshold set at an adjusted *p*‐value below 0.05. Reclustering analyses of immune cells were performed via the *Seurat* package, following the above‐mentioned pipeline. After quality control, normalization, scaling, PCA, *FindClusters* function (50 dimensions), and *FindMarkers* function (resolution: 0.5), common markers were employed to annotate immune subclusters. Specifically, signature genes for myeloid cell reclustering were adapted from a classic scRNA‐seq analysis pipeline contributed by Zhang et al.^[^ ^44^ ^]^

### Cellular Interaction Analysis

Analysis of cell‐to‐cell communication numbers and intensities was carried out using the *CellChat* package (version 1.6.1) with the CellChatDB.mouse database.^[^ ^72^ ^]^ The interaction within multiple components of the TME was evaluated with default parameters. Visualization of cell communication was facilitated through *netVisual* functions of the *CellChat* package. Interaction intensity was presented using heatmaps generated by the *netVisual_heatmap* function. The *netAnalysis_signalingChanges_scatter* function was utilized to visualize differential outgoing and incoming signaling pathways. Ligand‐receptor pairs contributing to CCL and CXCL signaling interactions were compared and visualized using *netVisual_bubble* function.

### Flow Cytometry for the TME Exploration

Tumor tissues were minced and incubated with a dissociation buffer (RPMI‐1640 medium containing 200 U mL^−1^ DNase‐I and 1 mg mL^−1^ Collagenase IV) at 37 °C for 30 min. Following filtration through a 40 µm cell strainer and treatment with red blood cell lysis buffer, dead cells were stained with Fixable Viability Dye eFluor 780, and anti‐CD16/CD32 antibody was added to minimize nonspecific staining in subsequent steps. Flow cytometry staining antibodies for in vivo experiments were listed in Table S14 (Supporting Information). Other auxiliary reagents, including Leukocyte Activation Cocktail with BD GolgiPlug, FOXP3 Fix/Perm Buffer Set, and Cell Staining Buffer were used in the assay. The cell number per 100 mg of fresh tissue was measured using a Vi‐Cell Auto instrument. All flow cytometry experiments were conducted on the BD Symphony A3 platform and analyzed using FlowJo software (10.8.1, BD).

### IF Staining

Fresh tumors were fixed with 4% paraformaldehyde for 48 h, followed by embedding in paraffin wax and sectioning. IF staining was conducted utilizing the tyramide signal amplification technique. Antibodies recognizing CD3 (ab237721, Abcam), CD8 (ab217344, Abcam), CCR5 (YT0936, Immunoway), Arg2 (ab264066, Abcam), PD‐L1 (64988, CST), Perforin (31647, CST), and Collagen I (ab270993, Abcam) were utilized in IF assays according to the manufacturer's recommendations. Captured images were reviewed using the Caseviewer platform, and regions of interest (ROIs) were delineated by two pathologists. Quantitative or co‐localization analysis of images was performed using ImageJ software (1.53). Expression abundance was calculated based on integral optical density (IOD) or proportions of positive pixels.

### Flow Sorting and T Cell Co‐Incubation

To confirm the inhibitory function of CCR5^+^ T cells in immune responses, we utilized flow cytometry to sort these cell populations from EMT‐6 tumor tissues after YM101 treatment. The cell sorting assay was performed based on the MoFlo XDP FACS platform (Beckman Coulter). Concurrently, naïve T cells were isolated from the spleens of BALB/c mice using magnetic bead separation (Mouse CD3 T Cell Isolation Kit, 480024, BioLegend), labeled with CFSE (5 µm, 65‐0850‐84, ThermoFisher), and activated with anti‐CD3/CD28 (precoated anti‐CD3: 5 µg mL^−1^, 100302, BioLegend; anti‐CD28: 2 µg mL^−1^, 102116, BioLegend). These naïve T cells (1 × 10^6^ mL^−1^) were then co‐cultured with identical numbers of either CCR5^+^ T cells or CCR5^−^ T cells, in conditions with or without the addition of anti‐PD‐L1 antibody (10^5^ pM, YZY) or Arginase inhibitor (0.1 mg mL^−1^, HY‐155108, MCE). Three days later, the proliferation of naïve T cells was evaluated using the CFSE dilution assay. As these cells proliferate, the fluorescence intensity of CFSE decreases by half with each cell division. By measuring the proportion of progeny cells to the total T cell population, the relative proliferation rate was determined. The proliferation index for each experimental group was calculated by comparing the ratio of progeny cells in that group to the reference ratio observed in the CTL group. This comparison provided a parameter that represents the relative proliferation capacity of the cells in each group. Moreover, activation markers such as CD44 and CD69 were analyzed to assess T cell activation.

### In Vivo Immune Cell Depletion

Given the regulatory role of CCR5i on various immune cells within the TME, such as Tregs, MDSCs, and TAMs, the aim was to assess the dependence of the synergistic antitumor effect of Maraviroc and YM101 on these immunosuppressive cells. Hereto, these cell populations were selectively dependent on EMT‐6‐bearing mice. For macrophage depletion, clodronate liposomes (CLD‐Lp, 40337ES08) and PBS control liposomes (PBS‐Lp, 40338ES08) from Yeasen Biotechnology were used. Mice received 200 µL CLD‐Lp (5 mg mL^−1^, intraperitoneal injection) or PBS‐Lp prior to EMT‐6 cell inoculation, with additional doses every 4 days.^[^ ^73^ ^]^ To eliminate MDSCs in vivo, EMT‐6 bearing mice were treated with intraperitoneal injections of anti‐DR5 (50 µg per mouse, BE0161, BioXCell) or control IgG (Armenian hamster IgG, BE0091, BioXCell), with additional injections every 3 days.^[^ ^74^ ^]^ Treg depletion was achieved through a single intravenous injection of anti‐CD25 (400 µg per mouse, BE0012, BioXCell) or control IgG (rat IgG1, BE0088, BioXCell).^[^ ^75^ ^]^ For neutrophil depletion, an initial intraperitoneal injection of anti‐Ly6G (400 µg per mouse, BP0075‐1, BioXCell) or control IgG (Rat IgG2a, BP0089, BioXCell) was given, followed by 100 µg doses three times weekly.^[^ ^76^ ^]^ The efficacy of depletion was confirmed by the loss of the targeted immune cells in EMT‐6 tumor tissues, as determined by flow cytometry assays.

### Bulk RNA‐Seq

In bulk RNA‐seq assays, treatment was initiated when tumor volumes reached 300 mm^3^. EMT‐6 tumor tissues were collected for total RNA extraction using TRIzol reagent (15596026, Invitrogen). Following DNA digestion with DNase‐I, RNA quality was assessed based on the A260/A280 ratio and 1.5% agarose gel electrophoresis. RNA quantification was performed using the QubitTM RNA Broad Range Assay kit (Q10210, Life Technologies). Subsequently, stranded RNA sequencing library preparation was conducted using 2 µg total RNA, in combination with the KC‐Digital Stranded mRNA Library Prep Kit (DR08502, Wuhan Seqhealth) and Ribo‐off rRNA Depletion Kit (MRZG12324, Illumina), following the manufacturer's instructions. Library products within the range of 200–500 bps were enriched and sequenced using NovaSeq 6000 (Illumina). The Mus_musculus.GRCm38 reference genome was utilized for mapping deduplicated reads. Reads mapped to exons were counted and scaled in terms of RPKM (Reads Per Kilobase Million). A standard RNA‐seq analysis pipeline was employed for differential analyses. DEG was defined as a gene with more than a two‐fold difference in expression and an adjusted *p‐*value below 0.05. DEG analysis was performed using the *DESeq2* package (version 1.40.2), and visualization of DEG analysis was accomplished based on the *pheatmap* package (version 1.0.12). Functional enrichment was carried out using the *ClusterProfiler* package (version 4.8.2), with pathways having an adjusted *p*‐value below 0.05 considered statistically significant. Immune signatures were scored based on public gene lists and compared as previously described.^[^ ^42^ ^]^

### Statistical Analyses

Statistical analyses were predominantly carried out using GraphPad Prism 8 and R software. Student's *t*‐test, with or without Welch's correction, was used to compare two groups when the data followed a normal distribution. In cases where data did not meet normality assumptions, Mann–Whitney tests were employed. Survival analysis of tumor‐bearing mice was performed using the Log‐rank test. Data were presented as mean ± standard deviation (SD). All tests were two‐sided, and differences with a *p*‐value below 0.05 were considered significant.

## Conflict of Interest

J.Z., Y.Y., and P.Z. were employees of Wuhan YZY Biopharma Co., Ltd.

## Author Contributions

M.Y., T.L., and M.N. contributed equally to this work. M.Y., T.L., and M.N. conducted the experiments and drafted the manuscript. Y.W., B.Z., Z.S., S.H., C.Z., X.Z., J.Z., Y.Y., and P.Z. participated in the analysis and interpretation of data. Q.C., Z.D., and K.W. designed the work and supervised the study. All authors gave final approval of the version to be published and agreed to be accountable for all aspects of the work.

## Ethics Approval and Consent to Participate

The animal operations in this study were evaluated and approved by the Institutional Animal Care and Use Committee of The First Affiliated Hospital, College of Medicine, Zhejiang University (No. 2023–378).

## Supporting information



Supporting Information

## Data Availability

The data that support the findings of this study are available from the corresponding author upon reasonable request.

## References

[1] Y. Wang , H. Zhang , C. Liu , Z. Wang , W. Wu , N. Zhang , L. Zhang , J. Hu , P. Luo , J. Zhang , Z. Liu , Y. Peng , Z. Liu , L. Tang , Q. Cheng , J. Hematol. Oncol. 2022, 15, 111. 35978433 10.1186/s13045-022-01325-0 PMC9386972

[2] D. Wu , H. Huang , M. Zhang , Z. Li , S. Wang , Y. Yu , Y. Fang , N. Jiang , H. Miao , P. Ma , Y. Tang , N. Li , J. Hematol. Oncol. 2022, 15, 16. 35135567 10.1186/s13045-022-01227-1 PMC8822713

[3] K. C. Ohaegbulam , A. Assal , E. Lazar‐Molnar , Y. Yao , X. Zang , Trends Mol. Med. 2015, 21, 24. 25440090 10.1016/j.molmed.2014.10.009 PMC4282825

[4] W. Zou , J. D. Wolchok , L. Chen , Sci. Transl. Med. 2016, 8, 328rv4. 10.1126/scitranslmed.aad7118 PMC4859220 26936508

[5] M. F. Sanmamed , L. Chen , Cell 2018, 175, 313. 30290139 10.1016/j.cell.2018.09.035 PMC6538253

[6] H. Dong , S. E. Strome , D. R. Salomao , H. Tamura , F. Hirano , D. B. Flies , P. C. Roche , J. Lu , G. Zhu , K. Tamada , V. A. Lennon , E. Celis , L. Chen , Nat. Med. 2002, 8, 793. 12091876 10.1038/nm730

[7] L. Chen , X. Han , J. Clin. Invest. 2015, 125, 3384. 26325035 10.1172/JCI80011 PMC4588282

[8] M. Yi , M. Niu , L. Xu , S. Luo , K. Wu , J. Hematol. Oncol. 2021, 14, 10. 33413496 10.1186/s13045-020-01027-5 PMC7792099

[9] W. Ma , R. Xue , Z. Zhu , H. Farrukh , W. Song , T. Li , L. Zheng , C. X. Pan , Exp. Hematol. Oncol. 2023, 12, 10. 36647169 10.1186/s40164-023-00372-8 PMC9843946

[10] P. M. Forde , J. E. Chaft , K. N. Smith , V. Anagnostou , T. R. Cottrell , M. D. Hellmann , M. Zahurak , S. C. Yang , D. R. Jones , S. Broderick , R. J. Battafarano , M. J. Velez , N. Rekhtman , Z. Olah , J. Naidoo , K. A. Marrone , F. Verde , H. Guo , J. Zhang , J. X. Caushi , H. Y. Chan , J. W. Sidhom , R. B. Scharpf , J. White , E. Gabrielson , H. Wang , G. L. Rosner , V. Rusch , J. D. Wolchok , T. Merghoub , et al., N. Engl. J. Med. 2018, 378, 1976. 29658848 10.1056/NEJMoa1716078 PMC6223617

[11] C. Robert , J. Schachter , G. V. Long , A. Arance , J. J. Grob , L. Mortier , A. Daud , M. S. Carlino , C. McNeil , M. Lotem , J. Larkin , P. Lorigan , B. Neyns , C. U. Blank , O. Hamid , C. Mateus , R. Shapira‐Frommer , M. Kosh , H. Zhou , N. Ibrahim , S. Ebbinghaus , A. Ribas , N. Engl. J. Med. 2015, 372, 2521. 25891173 10.1056/NEJMoa1503093

[12] J. Weng , S. Li , Z. Zhu , Q. Liu , R. Zhang , Y. Yang , X. Li , J. Hematol. Oncol. 2022, 15, 95. 35842707 10.1186/s13045-022-01294-4 PMC9288068

[13] S. Zhu , Y. Wu , B. Song , M. Yi , Y. Yan , Q. Mei , K. Wu , J. Hematol. Oncol. 2023, 16, 100. 37641116 10.1186/s13045-023-01497-3 PMC10464091

[14] M. S. Carlino , J. Larkin , G. V. Long , Lancet 2021, 398, 1002. 34509219 10.1016/S0140-6736(21)01206-X

[15] P. Sharma , S. Goswami , D. Raychaudhuri , B. A. Siddiqui , P. Singh , A. Nagarajan , J. Liu , S. K. Subudhi , C. Poon , K. L. Gant , S. M. Herbrich , S. Anandhan , S. Islam , M. Amit , G. Anandappa , J. P. Allison , Cell 2023, 186, 1652. 37059068 10.1016/j.cell.2023.03.006

[16] X. Yang , L. Ma , X. Zhang , L. Huang , J. Wei , Exp Hematol. Oncol. 2022, 11, 11. 35236415 10.1186/s40164-022-00263-4 PMC8889667

[17] D. J. Olson , Z. Eroglu , B. Brockstein , A. S. Poklepovic , M. Bajaj , S. Babu , S. Hallmeyer , M. Velasco , J. Lutzky , E. Higgs , R. Bao , T. C. Carll , B. Labadie , T. Krausz , Y. Zha , T. Karrison , V. K. Sondak , T. F. Gajewski , N. I. Khushalani , J. J. Luke , J. Clin. Oncol. 2021, 39, 2647. 33945288 10.1200/JCO.21.00079 PMC8376314

[18] V. Verma , G. Sharma , A. Singh , Exp. Hematol. Oncol. 2019, 8, 5. 30740266 10.1186/s40164-019-0129-x PMC6360752

[19] M. D. Vesely , T. Zhang , L. Chen , Annu. Rev. Immunol. 2022, 40, 45. 35471840 10.1146/annurev-immunol-070621-030155

[20] D. Davar , A. K. Dzutsev , J. A. McCulloch , R. R. Rodrigues , J. M. Chauvin , R. M. Morrison , R. N. Deblasio , C. Menna , Q. Ding , O. Pagliano , B. Zidi , S. Zhang , J. H. Badger , M. Vetizou , A. M. Cole , M. R. Fernandes , S. Prescott , R. G. F. Costa , A. K. Balaji , A. Morgun , I. Vujkovic‐Cvijin , H. Wang , A. A. Borhani , M. B. Schwartz , H. M. Dubner , S. J. Ernst , A. Rose , Y. G. Najjar , Y. Belkaid , J. M. Kirkwood , et al., Science 2021, 371, 595. 33542131 10.1126/science.abf3363 PMC8097968

[21] T. Nakamura , T. Sato , R. Endo , S. Sasaki , N. Takahashi , Y. Sato , M. Hyodo , Y. Hayakawa , H. Harashima , J. Immunother. Cancer 2021, 9, e002852. 34215690 10.1136/jitc-2021-002852 PMC8256839

[22] M. Yi , X. Zheng , M. Niu , S. Zhu , H. Ge , K. Wu , Mol. Cancer 2022, 21, 28. 35062949 10.1186/s12943-021-01489-2 PMC8780712

[23] Z. Liu , X. Yu , L. Xu , Y. Li , C. Zeng , Exp. Hematol. Oncol. 2022, 11, 44. 35907881 10.1186/s40164-022-00297-8 PMC9338491

[24] M. Yi , T. Li , M. Niu , Q. Mei , B. Zhao , Q. Chu , Z. Dai , K. Wu , Mol. Cancer 2023, 22, 187. 38008741 10.1186/s12943-023-01885-w PMC10680233

[25] S. Mariathasan , S. J. Turley , D. Nickles , A. Castiglioni , K. Yuen , Y. Wang , E. E. Kadel III , H. Koeppen , J. L. Astarita , R. Cubas , S. Jhunjhunwala , R. Banchereau , Y. Yang , Y. Guan , C. Chalouni , J. Ziai , Y. Şenbabaoğlu , S. Santoro , D. Sheinson , J. Hung , J. M. Giltnane , A. A. Pierce , K. Mesh , S. Lianoglou , J. Riegler , R. A. D. Carano , P. Eriksson , M. Höglund , L. Somarriba , D. L. Halligan , et al., Nature 2018, 554, 544. 29443960 10.1038/nature25501 PMC6028240

[26] D. V. F. Tauriello , S. Palomo‐Ponce , D. Stork , A. Berenguer‐Llergo , J. Badia‐Ramentol , M. Iglesias , M. Sevillano , S. Ibiza , A. Cañellas , X. Hernando‐Momblona , D. Byrom , J. A. Matarin , A. Calon , E. I. Rivas , A. R. Nebreda , A. Riera , C. S. Attolini , E. Batlle , Nature 2018, 554, 538. 29443964 10.1038/nature25492

[27] X. Shi , J. Yang , S. Deng , H. Xu , D. Wu , Q. Zeng , S. Wang , T. Hu , F. Wu , H. Zhou , J. Hematol. Oncol. 2022, 15, 135. 36115986 10.1186/s13045-022-01349-6 PMC9482317

[28] M. Niu , M. Yi , Y. Wu , L. Lyu , Q. He , R. Yang , L. Zeng , J. Shi , J. Zhang , P. Zhou , T. Zhang , Q. Mei , Q. Chu , K. Wu , J. Hematol. Oncol. 2023, 16, 94. 37573354 10.1186/s13045-023-01487-5 PMC10423429

[29] E. Batlle , J. Massagué , Immunity 2019, 50, 924. 30995507 10.1016/j.immuni.2019.03.024 PMC7507121

[30] B. G. Kim , E. Malek , S. H. Choi , J. J. Ignatz‐Hoover , J. J. Driscoll , J. Hematol. Oncol. 2021, 14, 55. 33823905 10.1186/s13045-021-01053-x PMC8022551

[31] L. Guo , D. Kong , J. Liu , L. Zhan , L. Luo , W. Zheng , Q. Zheng , C. Chen , S. Sun , Exp. Hematol. Oncol. 2023, 12, 3. 36624542 10.1186/s40164-022-00363-1 PMC9830930

[32] M. Yi , T. Li , M. Niu , Y. Wu , Z. Zhao , K. Wu , Front. Immunol. 2022, 13, 1061394. 36601124 10.3389/fimmu.2022.1061394 PMC9807229

[33] Y. Lan , T. L. Yeung , H. Huang , A. A. Wegener , S. Saha , M. Toister‐Achituv , M. H. Jenkins , L. Y. Chiu , A. Lazorchak , O. Tarcic , H. Wang , J. Qi , G. Locke , D. Kalimi , G. Qin , B. Marelli , H. Yu , A. W. Gross , M. G. Derner , M. Soloviev , M. Botte , A. Sircar , H. Ma , V. D. Sood , D. Zhang , F. Jiang , K. M. Lo , J. Immunother. Cancer 2022, 10, e004122. 35858707 10.1136/jitc-2021-004122 PMC9305820

[34] D. Peng , M. Fu , M. Wang , Y. Wei , X. Wei , Mol. Cancer 2022, 21, 104. 35461253 10.1186/s12943-022-01569-x PMC9033932

[35] H. Lind , S. R. Gameiro , C. Jochems , R. N. Donahue , J. Strauss , J. M. Gulley , C. Palena , J. Schlom , J. Immunother. Cancer 2020, 8, e000433. 32079617 10.1136/jitc-2019-000433 PMC7057416

[36] Y. Lan , D. Zhang , C. Xu , K. W. Hance , B. Marelli , J. Qi , H. Yu , G. Qin , A. Sircar , V. M. Hernández , M. H. Jenkins , R. E. Fontana , A. Deshpande , G. Locke , H. Sabzevari , L. Radvanyi , K. M. Lo , Sci. Transl. Med. 2018, 10, eaan5488. 29343622 10.1126/scitranslmed.aan5488

[37] L. Paz‐Ares , T. M. Kim , D. Vicente , E. Felip , D. H. Lee , K. H. Lee , C. C. Lin , M. J. Flor , M. Di Nicola , R. M. Alvarez , I. Dussault , C. Helwig , L. S. Ojalvo , J. L. Gulley , B. C. Cho , J. Thorac. Oncol. 2020, 15, 1210. 32173464 10.1016/j.jtho.2020.03.003 PMC8210474

[38] M. Yi , Y. Wu , M. Niu , S. Zhu , J. Zhang , Y. Yan , P. Zhou , Z. Dai , K. Wu , J. Immunother. Cancer 2022, 10, e005543. 36460337 10.1136/jitc-2022-005543 PMC9723957

[39] J. Strauss , C. R. Heery , J. Schlom , R. A. Madan , L. Cao , Z. Kang , E. Lamping , J. L. Marté , R. N. Donahue , I. Grenga , L. Cordes , O. Christensen , L. Mahnke , C. Helwig , J. L. Gulley , Clin. Cancer Res. 2018, 24, 1287. 29298798 10.1158/1078-0432.CCR-17-2653 PMC7985967

[40] B. Cheng , K. Ding , P. Chen , J. Ji , T. Luo , X. Guo , W. Qiu , C. Ma , X. Meng , J. Wang , J. Yu , Y. Liu , Cancer Commun. 2022, 42, 17. 10.1002/cac2.12244 PMC8753312 34981670

[41] M. Yi , M. Niu , J. Zhang , S. Li , S. Zhu , Y. Yan , N. Li , P. Zhou , Q. Chu , K. Wu , J. Hematol. Oncol. 2021, 14, 146. 34526097 10.1186/s13045-021-01155-6 PMC8442312

[42] M. Yi , J. Zhang , A. Li , M. Niu , Y. Yan , Y. Jiao , S. Luo , P. Zhou , K. Wu , J. Hematol. Oncol. 2021, 14, 27. 33593403 10.1186/s13045-021-01045-x PMC7885589

[43] S. Zhu , M. Yi , Y. Wu , B. Dong , K. Wu , Exp. Hematol. Oncol. 2021, 10, 60. 34965886 10.1186/s40164-021-00252-z PMC8715617

[44] L. Zhang , Z. Li , K. M. Skrzypczynska , Q. Fang , W. Zhang , S. A. O'Brien , Y. He , L. Wang , Q. Zhang , A. Kim , R. Gao , J. Orf , T. Wang , D. Sawant , J. Kang , D. Bhatt , D. Lu , C. M. Li , A. S. Rapaport , K. Perez , Y. Ye , S. Wang , X. Hu , X. Ren , W. Ouyang , Z. Shen , J. G. Egen , Z. Zhang , X. Yu , Cell 2020, 181, 442. 32302573 10.1016/j.cell.2020.03.048

[45] J. Wang , M. T. Saung , K. Li , J. Fu , K. Fujiwara , N. Niu , S. Muth , J. Wang , Y. Xu , N. Rozich , H. Zlomke , S. Chen , B. Espinoza , M. Henderson , V. Funes , B. Herbst , D. Ding , C. Twyman‐Saint Victor , Q. Zhao , A. Narang , J. He , L. Zheng , J. Exp. Med. 2022, 219, e20211631. 35404390 10.1084/jem.20211631 PMC9006312

[46] D. Hirschhorn , S. Budhu , L. Kraehenbuehl , M. Gigoux , D. Schröder , A. Chow , J. M. Ricca , B. Gasmi , O. De Henau , L. M. B. Mangarin , Y. Li , L. Hamadene , A. L. Flamar , H. Choi , C. A. Cortez , C. Liu , A. Holland , S. Schad , I. Schulze , A. Betof Warner , T. J. Hollmann , A. Arora , K. S. Panageas , G. A. Rizzuto , R. Duhen , A. D. Weinberg , C. N. Spencer , D. Ng , X. Y. He , J. Albrengues , et al., Cell 2023, 186, 1432. 37001503 10.1016/j.cell.2023.03.007 PMC10994488

[47] K. Li , J. A. Tandurella , J. Gai , Q. Zhu , S. J. Lim , D. L. Thomas 2nd , T. Xia , G. Mo , J. T. Mitchell , J. Montagne , M. Lyman , L. V. Danilova , J. W. Zimmerman , B. Kinny‐Köster , T. Zhang , L. Chen , A. B. Blair , T. Heumann , R. Parkinson , J. N. Durham , A. K. Narang , R. A. Anders , C. L. Wolfgang , D. A. Laheru , J. He , A. Osipov , E. D. Thompson , H. Wang , E. J. Fertig , E. M. Jaffee , et al., Cancer Cell 2022, 40, 1374. 36306792 10.1016/j.ccell.2022.10.001 PMC9669212

[48] X. Cheng , H. Wang , Z. Wang , B. Zhu , H. Long , J. Hematol. Oncol. 2023, 16, 71. 37415162 10.1186/s13045-023-01473-x PMC10324139

[49] M. Yi , T. Li , M. Niu , H. Zhang , Y. Wu , K. Wu , Z. Dai , Signal Transduct. Target. Ther. 2024, 9, 176. 39034318 10.1038/s41392-024-01868-3 PMC11275440

[50] S. Qin , L. Xu , M. Yi , S. Yu , K. Wu , S. Luo , Mol. Cancer 2019, 18, 155. 31690319 10.1186/s12943-019-1091-2 PMC6833286

[51] T. Zhou , W. Damsky , O. E. Weizman , M. K. McGeary , K. P. Hartmann , C. E. Rosen , S. Fischer , R. Jackson , R. A. Flavell , J. Wang , M. F. Sanmamed , M. W. Bosenberg , A. M. Ring , Nature 2020, 583, 609. 32581358 10.1038/s41586-020-2422-6 PMC7381364

[52] S. E. Weis‐Banke , T. L. Lisle , M. Perez‐Penco , A. Schina , M. L. Hübbe , M. Siersbæk , M. O. Holmström , M. A. Jørgensen , I. M Svane , Ö. Met , N. Ødum , D. H. Madsen , M. Donia , L. Grøntved , M. H. Andersen , J. Immunother. Cancer 2022, 10, e005326. 36316062 10.1136/jitc-2022-005326 PMC9628693

[53] X. Wang , K. E. Russell‐Lodrigue , M. S. Ratterree , R. S. Veazey , H. Xu , FASEB J. 2019, 33, 8905. 31034775 10.1096/fj.201802703R PMC6662974

[54] T. T. Murooka , M. M. Wong , R. Rahbar , B. Majchrzak‐Kita , A. E. Proudfoot , E. N. Fish , J. Biol. Chem. 2006, 281, 25184. 16807236 10.1074/jbc.M603912200

[55] S. T. Ward , K. K. Li , E. Hepburn , C. J. Weston , S. M. Curbishley , G. M. Reynolds , R. K. Hejmadi , R. Bicknell , B. Eksteen , T. Ismail , A. Rot , D. H. Adams , Br. J. Cancer 2015, 112, 319. 25405854 10.1038/bjc.2014.572 PMC4301825

[56] K. J. Gellatly , J. P. Strassner , K. Essien , M. A. Refat , R. L. Murphy , A. Coffin‐Schmitt , A. G. Pandya , A. Tovar‐Garza , M. L. Frisoli , X. Fan , X. Ding , E. E. Kim , Z. Abbas , P. McDonel , M. Garber , J. E. Harris , Sci. Transl. Med. 2021, 13, eabd8995. 34516831 10.1126/scitranslmed.abd8995 PMC8686160

[57] S. Li , Q. Xie , Y. Zeng , C. Zou , X. Liu , S. Wu , H. Deng , Y. Xu , X. C. Li , Z. Dai , Cell Mol. Immunol. 2014, 11, 326. 24793406 10.1038/cmi.2014.25 PMC4085522

[58] M. M. Lederman , A. Penn‐Nicholson , M. Cho , D. Mosier , JAMA 2006, 296, 815. 16905787 10.1001/jama.296.7.815

[59] X. Jiao , O. Nawab , T. Patel , A. V. Kossenkov , N. Halama , D. Jaeger , R. G. Pestell , Cancer Res. 2019, 79, 4801. 31292161 10.1158/0008-5472.CAN-19-1167 PMC6810651

[60] L. F. Gao , Y. Zhong , T. Long , X. Wang , J. X. Zhu , X. Y. Wang , Z. Y. Hu , Z. G. Li , J. Exp. Clin. Cancer Res. 2022, 41, 81. 35241150 10.1186/s13046-022-02300-w PMC8892738

[61] X. N. Zhang , K. D. Yang , C. Chen , Z. C. He , Q. H. Wang , H. Feng , S. Q. Lv , Y. Wang , M. Mao , Q. Liu , Y. Y. Tan , W. Y. Wang , T. R. Li , L. R. Che , Z. Y. Qin , L. X. Wu , M. Luo , C. H. Luo , Y. Q. Liu , W. Yin , C. Wang , H. T. Guo , Q. R. Li , B. Wang , W. Chen , S. Wang , Y. Shi , X. W. Bian , Y. F. Ping , Cell Res. 2021, 31, 1072. 34239070 10.1038/s41422-021-00528-3 PMC8486800

[62] N. Halama , I. Zoernig , A. Berthel , C. Kahlert , F. Klupp , M. Suarez‐Carmona , T. Suetterlin , K. Brand , J. Krauss , F. Lasitschka , T. Lerchl , C. Luckner‐Minden , A. Ulrich , M. Koch , J. Weitz , M. Schneider , M. W. Buechler , L. Zitvogel , T. Herrmann , A. Benner , C. Kunz , S. Luecke , C. Springfeld , N. Grabe , C. S. Falk , D. Jaeger , Cancer Cell 2016, 29, 587. 27070705 10.1016/j.ccell.2016.03.005

[63] X. Jiao , M. A. Velasco‐Velázquez , M. Wang , Z. Li , H. Rui , A. R. Peck , J. E. Korkola , X. Chen , S. Xu , J. B. DuHadaway , S. Guerrero‐Rodriguez , S. Addya , D. Sicoli , Z. Mu , G. Zhang , A. Stucky , X. Zhang , M. Cristofanilli , A. Fatatis , J. W. Gray , J. F. Zhong , G. C. Prendergast , R. G. Pestell , Cancer Res. 2018, 78, 1657. 29358169 10.1158/0008-5472.CAN-17-0915 PMC6331183

[64] M. Velasco‐Velázquez , X. Jiao , M. De La Fuente , T. G. Pestell , A. Ertel , M. P. Lisanti , R. G. Pestell , Cancer Res. 2012, 72, 3839. 22637726 10.1158/0008-5472.CAN-11-3917

[65] D. Aldinucci , C. Borghese , N. Casagrande , Cancers (Basel) 2020, 12, 1765. 32630699 10.3390/cancers12071765 PMC7407580

[66] L. Y. Chang , Y. C. Lin , J. Mahalingam , C. T. Huang , T. W. Chen , C. W. Kang , H. M. Peng , Y. Y. Chu , J. M. Chiang , A. Dutta , Y. J. Day , T. C. Chen , C. T. Yeh , C. Y. Lin , Cancer Res. 2012, 72, 1092. 22282655 10.1158/0008-5472.CAN-11-2493

[67] K. Litchfield , J. L. Reading , C. Puttick , K. Thakkar , C. Abbosh , R. Bentham , T. B. K. Watkins , R. Rosenthal , D. Biswas , A. Rowan , E. Lim , M. Al Bakir , V. Turati , J. A. Guerra‐Assunção , L. Conde , A. J. S. Furness , S. K. Saini , S. R. Hadrup , J. Herrero , S. H. Lee , P. Van Loo , T. Enver , J. Larkin , M. D. Hellmann , S. Turajlic , S. A. Quezada , N. McGranahan , C. Swanton , Cell 2021, 184, 596. 33508232 10.1016/j.cell.2021.01.002 PMC7933824

[68] Z. Zhang , Y. Li , S. Jiang , F. D. Shi , K. Shi , W. N. Jin , CNS Neurosci. Ther. 2023, 29, 317. 36440924 10.1111/cns.14006 PMC9804050

[69] C. M. Carlson , J. L. Frandsen , N. Kirchhof , R. S. McIvor , D. A. Largaespada , Proc. Natl. Acad. Sci. USA 2005, 102, 17059. 16286660 10.1073/pnas.0502974102 PMC1287966

[70] M. Yi , M. Niu , Y. Wu , H. Ge , D. Jiao , S. Zhu , J. Zhang , Y. Yan , P. Zhou , Q. Chu , K. Wu , J. Hematol. Oncol. 2022, 15, 142. 36209176 10.1186/s13045-022-01363-8 PMC9548169

[71] L. Zheng , S. Qin , W. Si , A. Wang , B. Xing , R. Gao , X. Ren , L. Wang , X. Wu , J. Zhang , N. Wu , N. Zhang , H. Zheng , H. Ouyang , K. Chen , Z. Bu , X. Hu , J. Ji , Z. Zhang , Science 2021, 374, abe6474. 34914499 10.1126/science.abe6474

[72] S. Jin , C. F. Guerrero‐Juarez , L. Zhang , I. Chang , R. Ramos , C. H. Kuan , P. Myung , M. V. Plikus , Q. Nie , Nat. Commun. 2021, 12, 1088. 33597522 10.1038/s41467-021-21246-9 PMC7889871

[73] D. Sheng , W. Ma , R. Zhang , L. Zhou , Q. Deng , J. Tu , W. Chen , F. Zhang , N. Gao , M. Dong , D. Wang , F. Li , Y. Liu , X. He , S. Duan , L. Zhang , T. Liu , S. Liu , J. Immunother. Cancer 2022, 10, e003793. 35613826 10.1136/jitc-2021-003793 PMC9134178

[74] Y. Tang , C. Zhou , Q. Li , X. Cheng , T. Huang , F. Li , L. He , B. Zhang , S. Tu , Oncoimmunology 2022, 11, 2131084. 36268178 10.1080/2162402X.2022.2131084 PMC9578486

[75] M. L. Miller , M. D. Daniels , T. Wang , J. Chen , J. Young , J. Xu , Y. Wang , D. Yin , V. Vu , A. N. Husain , M. L. Alegre , A. S. Chong , Nat. Commun. 2015, 6, 7566. 26151823 10.1038/ncomms8566 PMC4498267

[76] S. B. Coffelt , K. Kersten , C. W. Doornebal , J. Weiden , K. Vrijland , C. S. Hau , N. J. M. Verstegen , M. Ciampricotti , L. Hawinkels , J. Jonkers , K. E. de Visser , Nature 2015, 522, 345. 25822788 10.1038/nature14282 PMC4475637

