# Supplementary material for: Blockade of CCR5+ T Cell Accumulation in the Tumor Microenvironment Optimizes Anti‐TGF‐β/PD‐L1 Bispecific Antibody
Source: Adv Sci (Weinh). 2024 Sep 20;11(43):2408598. doi: 10.1002/advs.202408598 (PMC11578335; doi:10.1002/advs.202408598)
Supplement: Supplementary file 1 — Supporting Information [file ADVS-11-2408598-s001.docx]

**Supporting Information**

**Blockade of CCR5^+^ T Cell Accumulation in the Tumor Microenvironment Optimizes Anti-TGF-β/PD-L1 Bispecific Antibody**

Ming Yi^1,2#^, Tianye Li^3#^, Mengke Niu^4,5#^, Yuze Wu^5^, Bin Zhao^1^, Zhuoyang Shen^1^, Shengtao Hu^1^, Chaomei Zhang^1^, Xiaojun Zhang^2^, Jing Zhang^6^, Yongxiang Yan^6^, Pengfei Zhou^6^, Qian Chu^5*^, Zhijun Dai^1*^, Kongming Wu^2,5*^

1. Department of Breast Surgery, The First Affiliated Hospital, College of Medicine, Zhejiang University, Hangzhou, 310000, People's Republic of China.
2. Cancer Center, Shanxi Bethune Hospital, Shanxi Academy of Medical Science, Tongji Shanxi Hospital, Third Hospital of Shanxi Medical University, Taiyuan, 030032, People's Republic of China.
3. Department of Gynecology, The Second Affiliated Hospital of Zhejiang University School of Medicine, Hangzhou, 310009, People's Republic of China.
4. Department of Medical Oncology, The First Affiliated Hospital, College of Medicine, Zhejiang University, Hangzhou, 310000, People's Republic of China.
5. Department of Oncology, Tongji Hospital of Tongji Medical College, Huazhong University of Science and Technology, Wuhan, 430030, People's Republic of China.
6. Wuhan YZY Biopharma Co., Ltd, Biolake, C2-1, No.666 Gaoxin Road, Wuhan, 430075, People's Republic of China.

**# Equal contribution**

*** Corresponding authors**

Kongming Wu, Department of Oncology, Tongji Hospital of Tongji Medical College, Huazhong University of Science and Technology, 1095 Jiefang Avenue, Wuhan, 430030, People's Republic of China. E-mail: kmwu@tjh.tjmu.edu.cn.

Zhijun Dai, Department of Breast Surgery, The First Affiliated Hospital, College of Medicine, Zhejiang University, Hangzhou, 310000, People's Republic of China. E-mail: dzj0911@126.com.

Qian Chu, Department of Oncology, Tongji Hospital of Tongji Medical College, Huazhong University of Science and Technology, Wuhan, 430030, People's Republic of China. E-mail: qianchu@tjh.tjmu.edu.cn.

**Running title:** CCR5 blockade plus anti-TGF-β/PD-L1 bispecific antibody


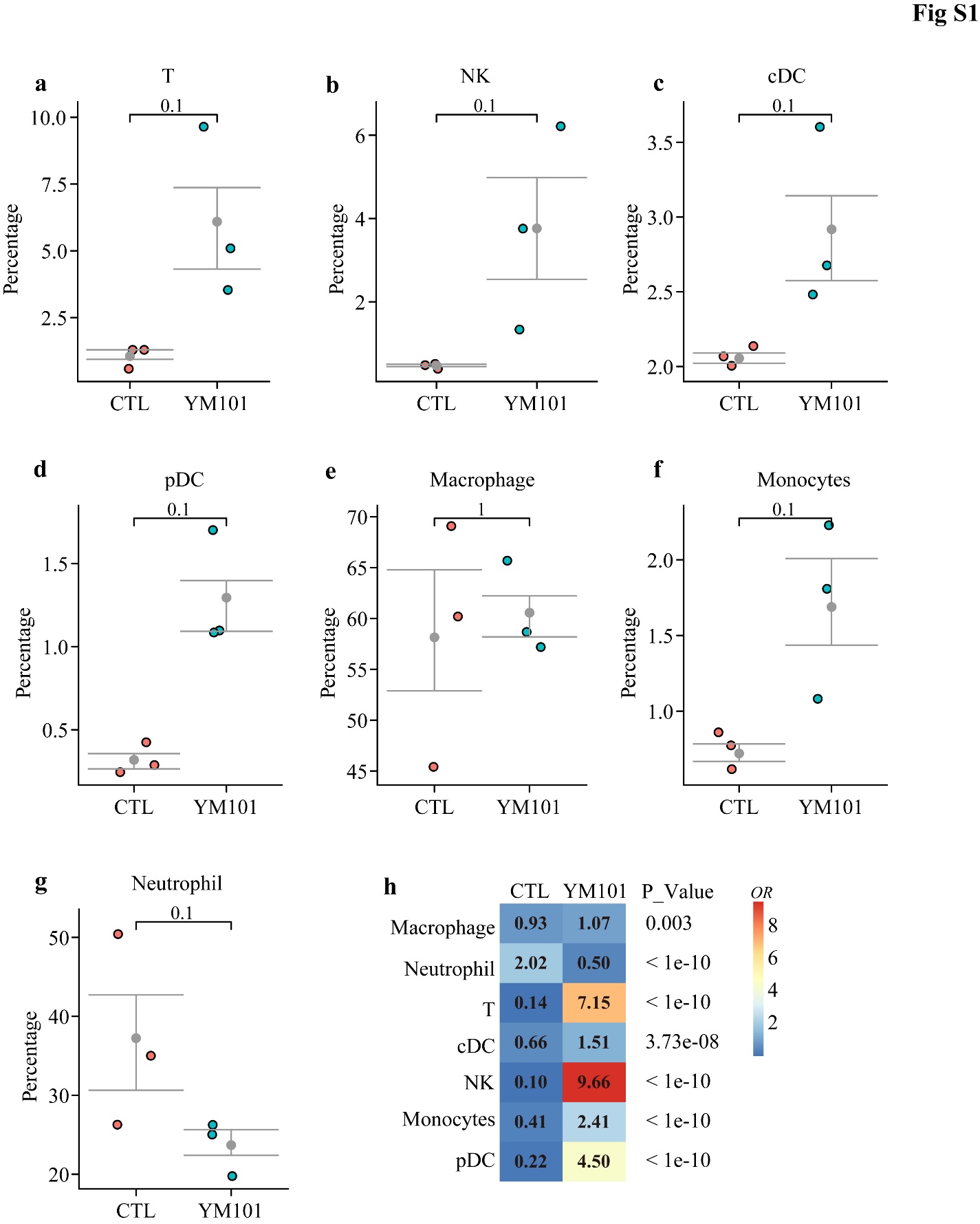


**Figure S1.** Preference differences in immune cell distribution between CTL and YM101 groups. (a-f) Scatter plots illustrating the percentages of various immune cells in the CTL and YM101 treatment groups. Each point represents an individual measurement from distinct samples. The subpopulations are as follows: (a) T cells, (b) NK cells, (c) cDC cells, (d) pDC cells, (e) Macrophages, (f) Monocytes, and (g) Neutrophils. The percentage of each cell type is calculated relative to the total immune cell population. (h) Heat map reflecting the preference difference in cell distribution between CTL and YM101 groups, measured by odds ratios (OR). The heat map's color gradient spans from blue to red, indicating lower to higher preference, respectively. The *P*-values denote the significance of the difference in distribution.


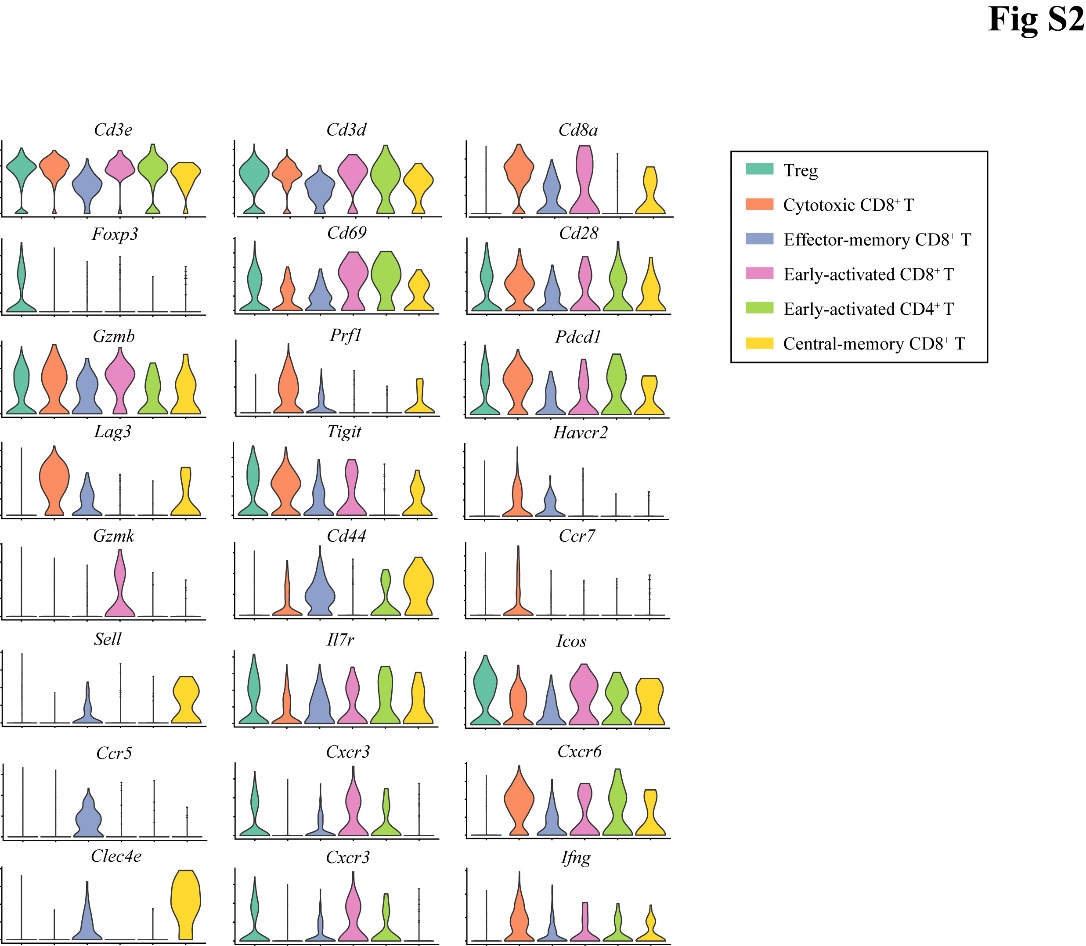


**Figure S2:** Violin plots showing the expression levels of common T cell markers.


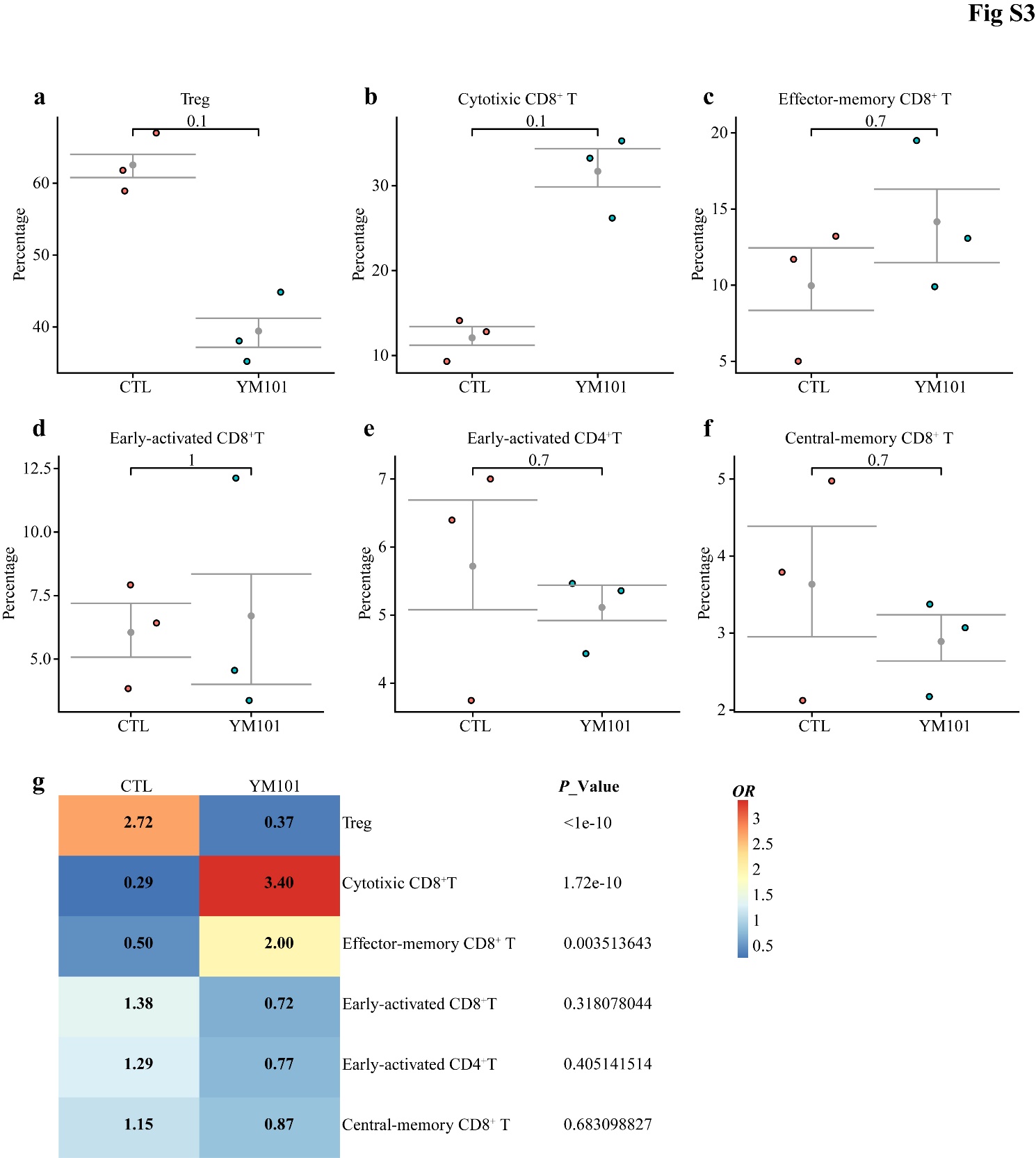


**Figure S3.** Preference differences in T cell subpopulation distribution between CTL and YM101 groups. (a-f) Scatter plots illustrating the percentages of various T cell subpopulations in the CTL and YM101 treatment groups. Each point represents an individual measurement from distinct samples. The percentage of each T cell subset is calculated relative to the total T cell population. The subpopulations are as follows: (a) Regulatory T cells (Treg), (b) Cytotoxic CD8^+^ T cells, (c) Effector-memory CD8^+^ T cells, (d) Early-activated CD8^+^ T cells, (e) Early-activated CD4^+^ T cells, and (f) Central-memory CD8^+^ T cells. (g) Heat map reflecting the preference difference in cell distribution between CTL and YM101 groups, measured by odds ratios (OR). The heat map's color gradient spans from blue to red, indicating lower to higher preference, respectively. The *P*-values denote the significance of the difference in distribution.


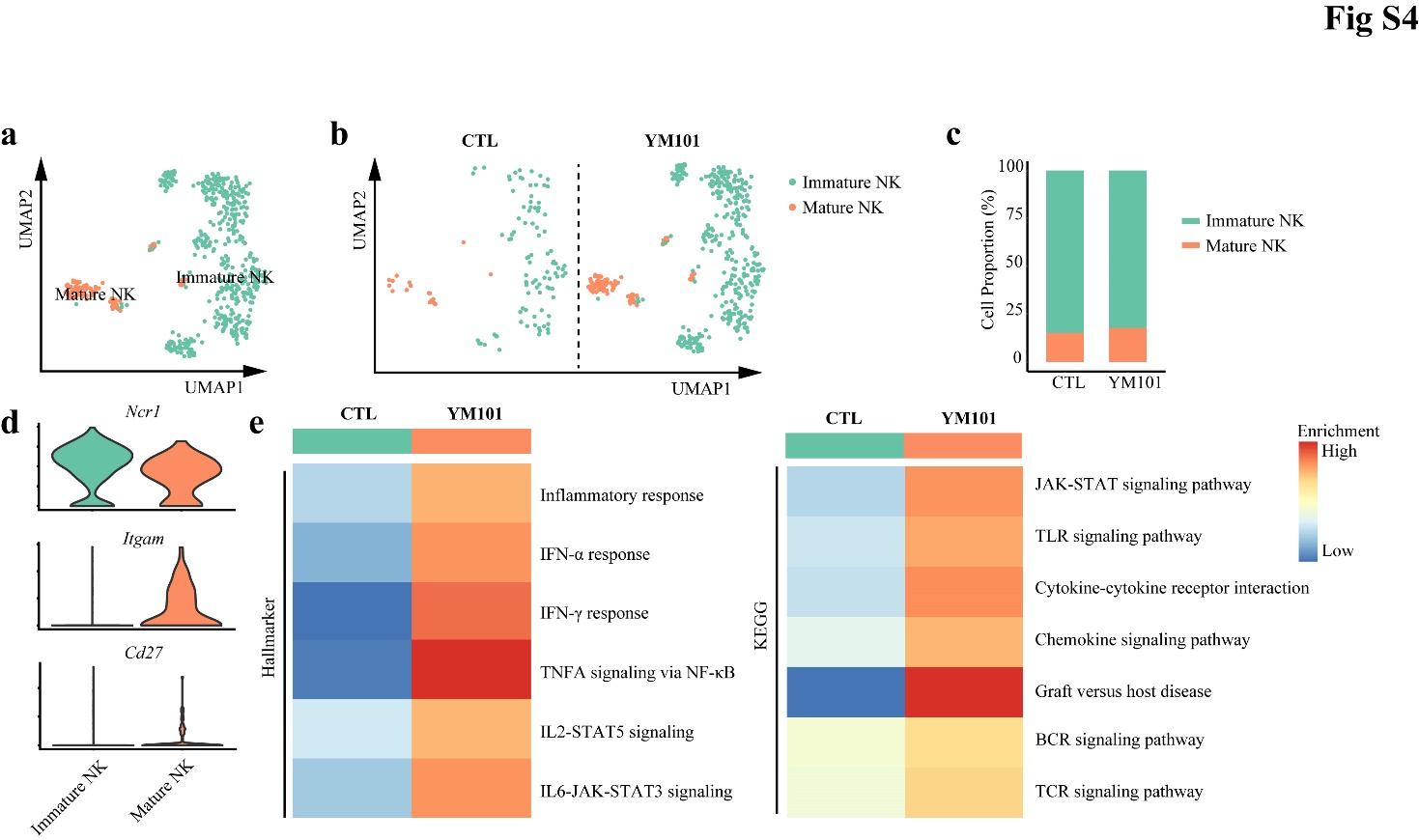


**Figure S4.** Secondary analysis of tumor-infiltrating NK cells. (a) Uniform Manifold Approximation and Projection (UMAP) plot for NK cells. (b) UMPA plot for NK cell subclusters in different groups. (c) Histogram representing the proportion of NK cell subclusters in different groups. (d) Violin plots showing the expression levels of specific markers. (e) Heatmap depicting the results of GSEA using Hallmarker and KEGG sets.


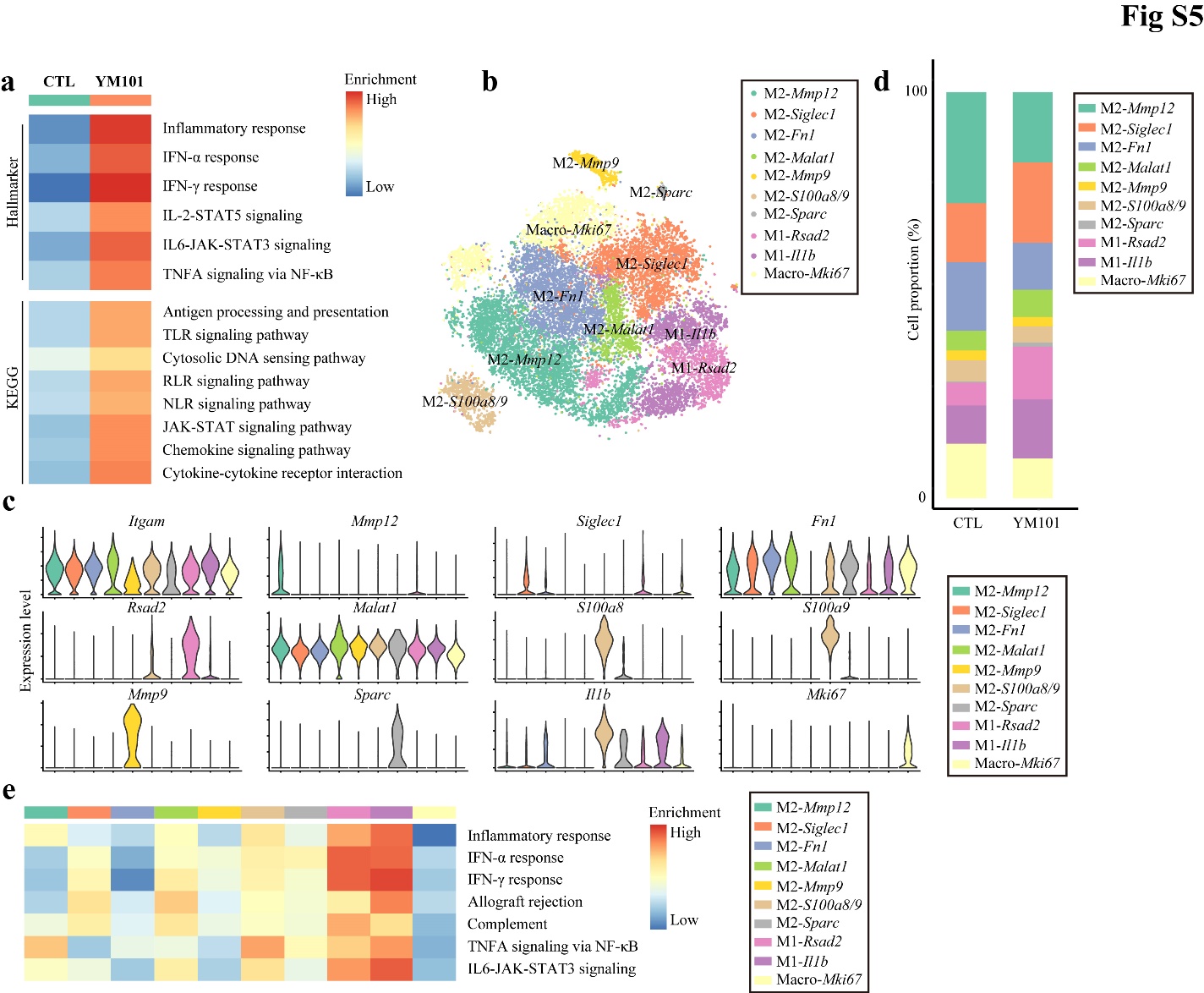


**Figure S5:** Secondary analysis of tumor-infiltrating macrophages. (a) Heatmap showing GSEA results of total macrophages. (b) T-distributed stochastic neighbor embedding (t-SNE) plot showing the results of reclustering analysis of macrophages. (c) Violin plots showing the expression levels of common macrophage markers. (d) Histogram representing the proportions of macrophage subsets in the CTL and YM101 group. (e) Heatmap depicting the intrinsic features of macrophage subsets by GSEA.


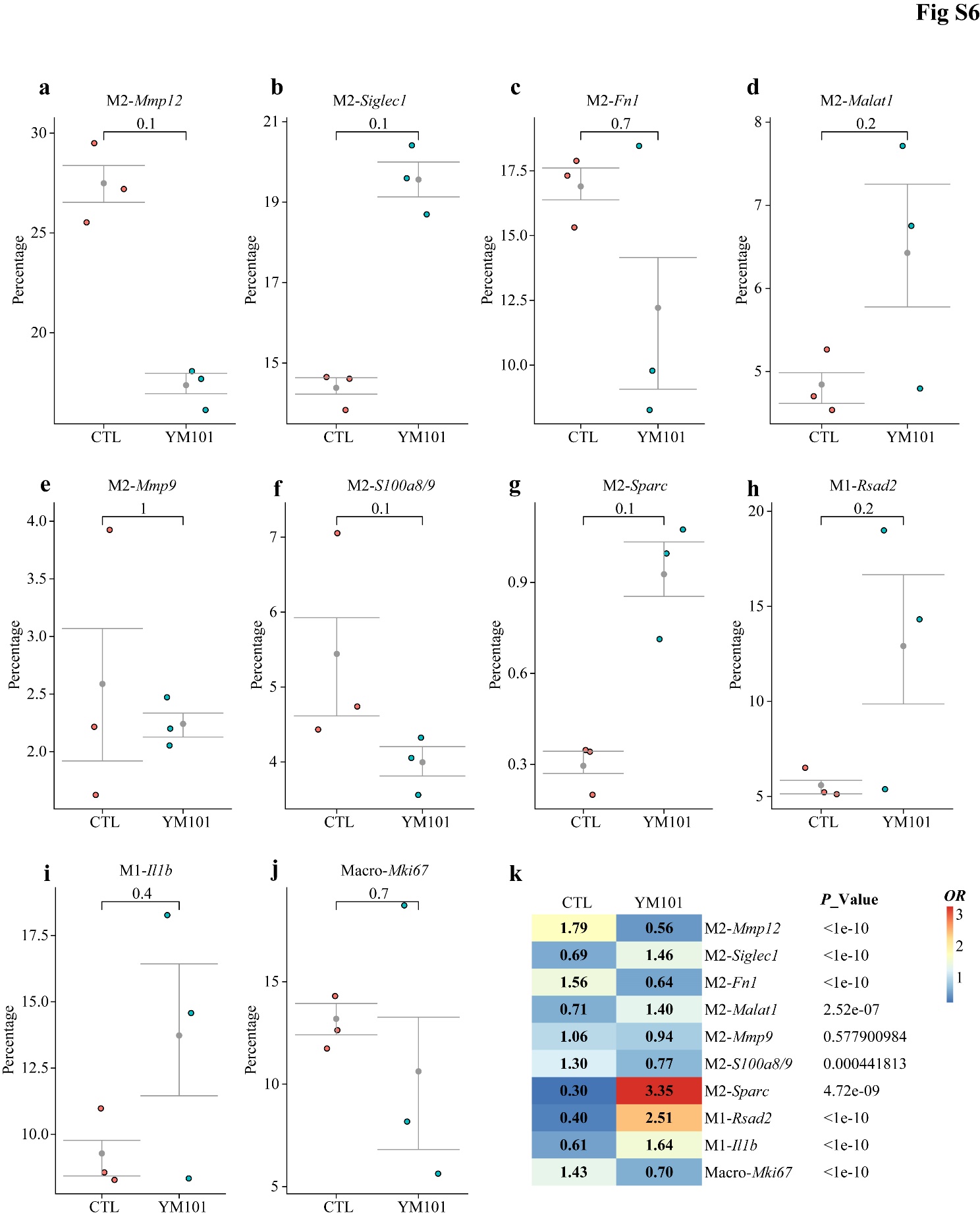


**Figure S6.** Preference differences in macrophage subpopulation distribution between CTL and YM101 groups. (a-j) Scatter plots illustrating the percentages of various macrophage subpopulations in the CTL and YM101 treatment groups. The subpopulations are as follows: (a) M2-*Mmp12*, (b) M2-*Siglec1*, (c) M2-*Fn1*, (d) M2-*Malat1*, (e) M2-*Mmp9*, (f) M2-*S100a8/9*, (g) M2-*Sparc*, (h) M1-*Rsad2*, (i) M1-*Il1b*, (j) Macrophage-*Mki67*. Each point represents an individual measurement from distinct samples. The percentage of each macrophage subset is calculated relative to the total macrophage population. (k) Heat map reflecting the preference difference in cell distribution between CTL and YM101 groups, measured by odds ratios (OR). The heat map's color gradient spans from blue to red, indicating lower to higher preference, respectively. The *P*-values denote the significance of the difference in distribution.


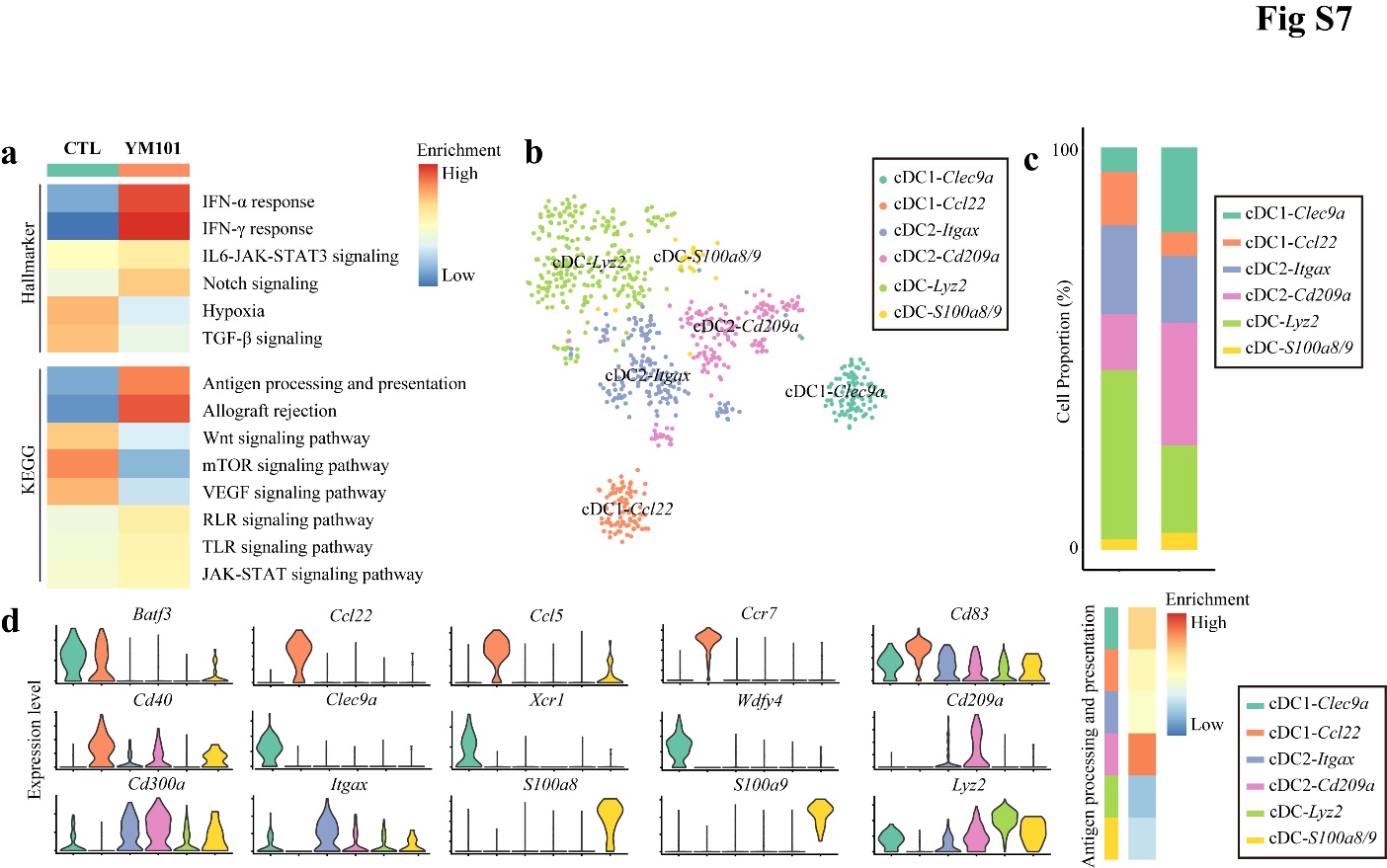


**Figure S7: Secondary analysis of tumor-infiltrating cDCs. (a)** Heatmap showing GSEA results of total cDCs. **(b)** t-SNE plot showing the results of reclustering analysis of cDCs. **(c)** Histogram representing the proportions of cDC subsets in the CTL and YM101 groups. **(d)** Depicting the intrinsic features of cDC subsets by specific markers and GSEA. Violin plots showing the expression levels of cDC-associated markers. Heatmap indicating the antigen presentation capability of cDC subsets.


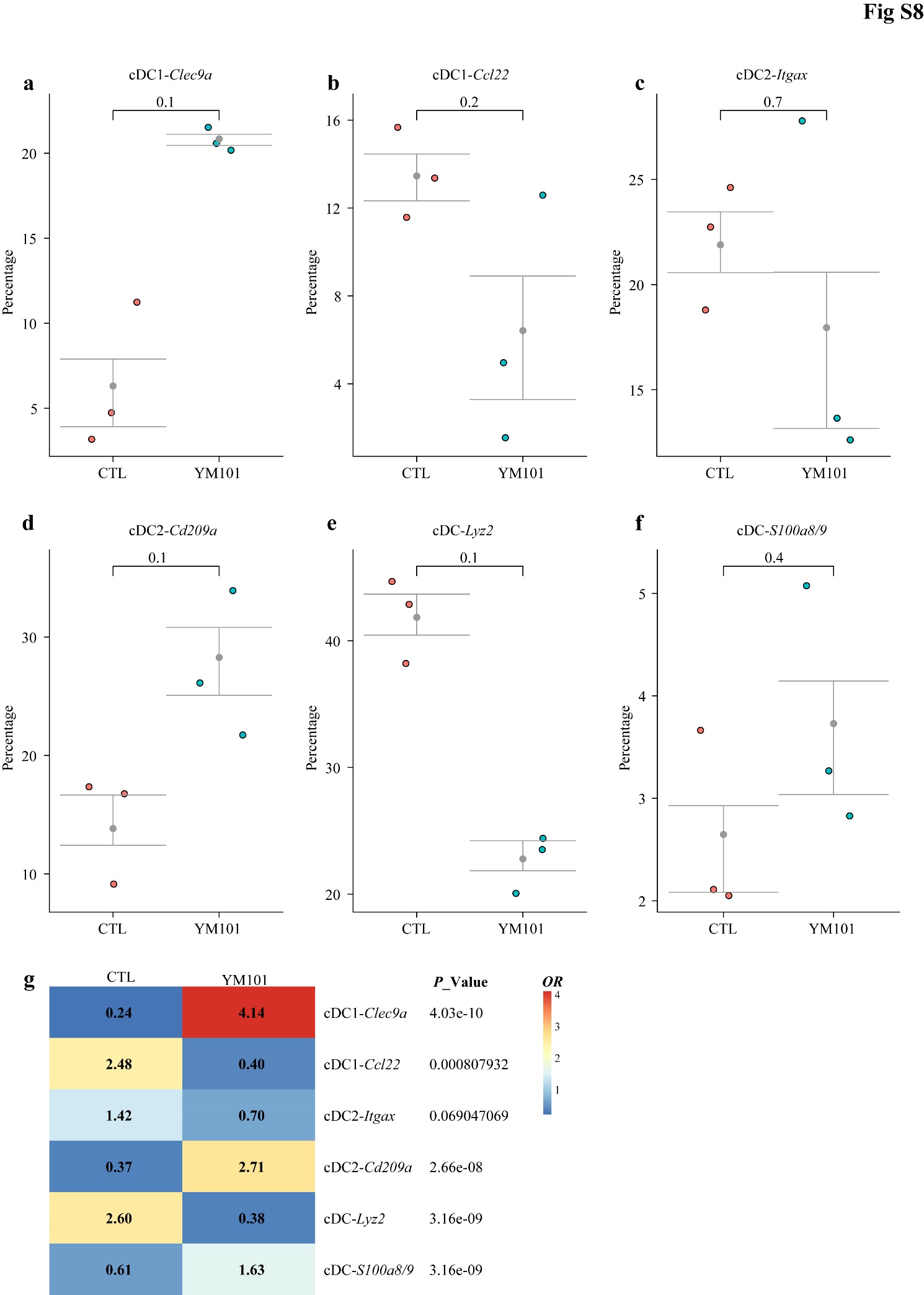


**Figure S8.** Preference differences in cDC subpopulation distribution between CTL and YM101 groups. (a-f) Scatter plots illustrating the percentages of various cDC subpopulations in the CTL and YM101 treatment groups. The subpopulations are as follows: (a) cDC1-*Clec9a*, (b) cDC1-*Ccl22*, (c) cDC2-*Itgax*, (d) cDC2-*Cd209a*, (e) cDC-*Lyz2*, (f) cDC-*S100a8/9*. Each point represents an individual measurement from distinct samples. The percentage of each cDC subset is calculated relative to the total cDC population. (g) Heat map reflecting the preference difference in cell distribution between CTL and YM101 groups, measured by odds ratios (OR). The heat map's color gradient spans from blue to red, indicating lower to higher preference, respectively. The *P*-values denote the significance of the difference in distribution.


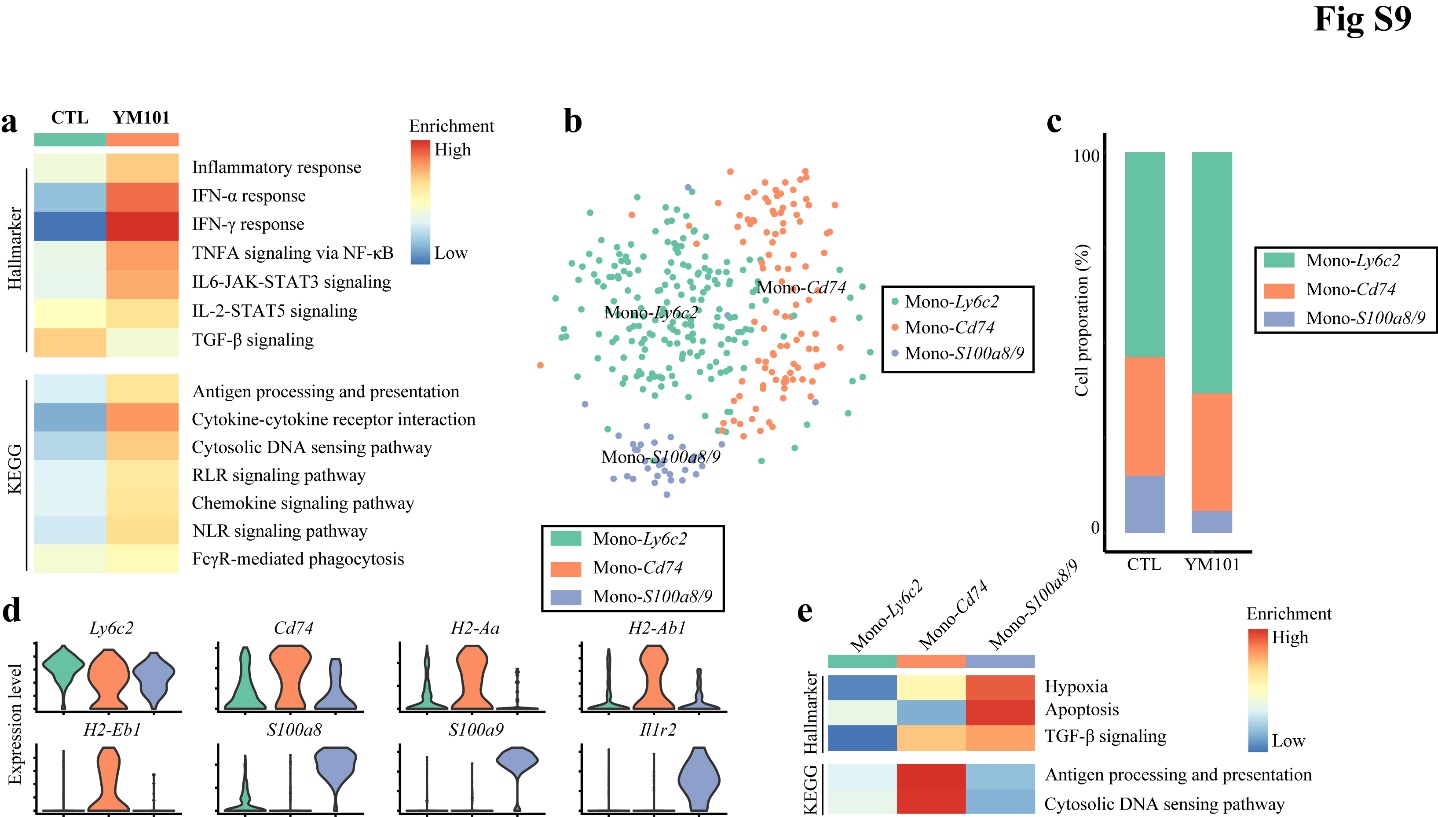


**Figure S9:** Secondary analysis of tumor-infiltrating monocytes. (a) Heatmap showing GSEA results of total monocytes. (b) T-distributed stochastic neighbor embedding (t-SNE) plot showing the results of reclustering analysis of monocytes. (c) Histogram representing the proportions of monocyte subsets in the CTL and YM101 groups. (d) Violin plots showing the expression levels of monocyte-associated markers. (e) Heatmap depicting the intrinsic features of monocyte subsets by GSEA.


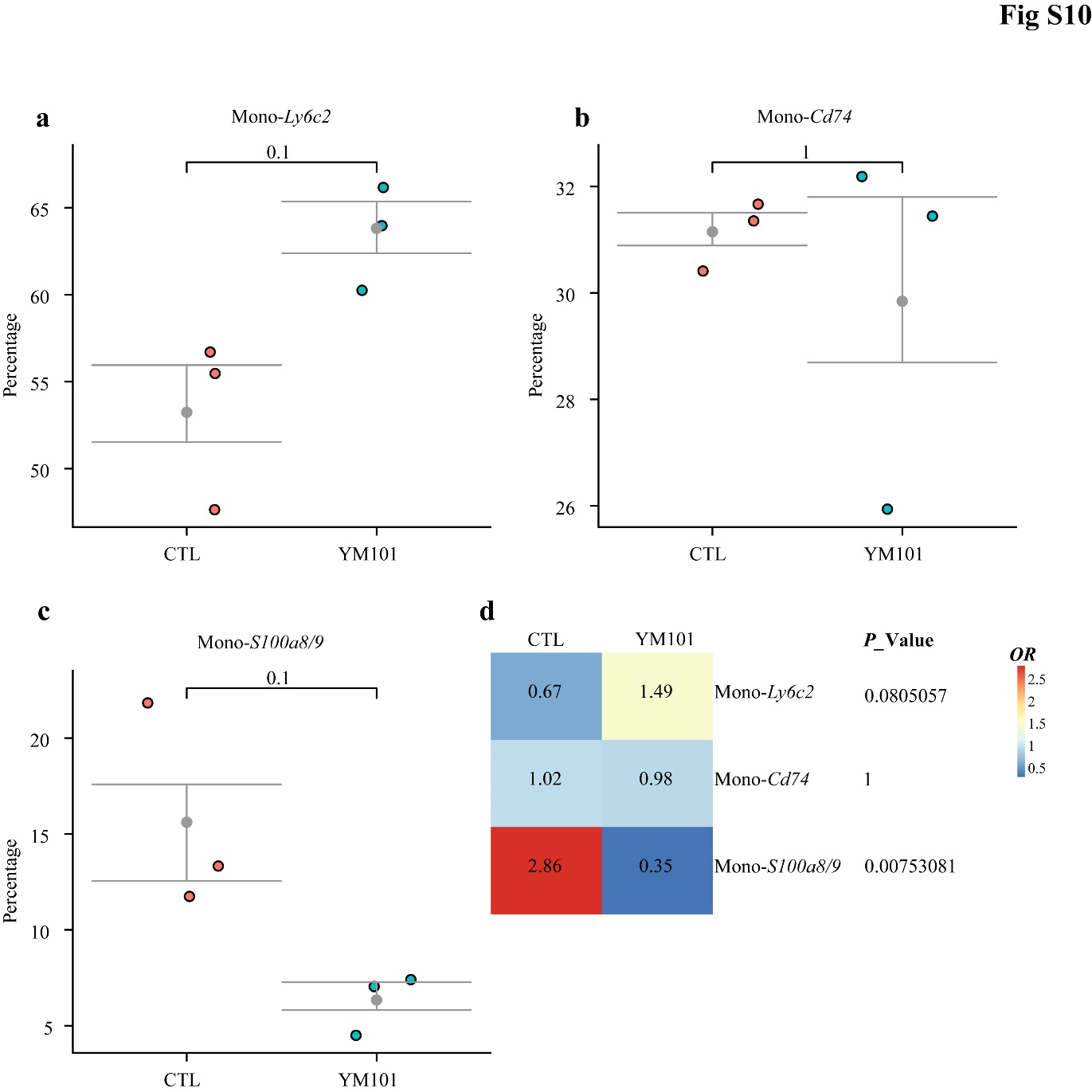


**Figure S10.** Preference differences in monocyte subpopulation distribution between CTL and YM101 groups. (a-c) Scatter plots illustrating the percentages of various monocyte subpopulations in the CTL and YM101 treatment groups. The subpopulations are as follows: (a) Monocyte-*Ly6c2*, (b) Monocyte-*Cd74*, (c) Monocyte-*S100a8/9*. Each point represents an individual measurement from distinct samples. The percentage of each monocyte subset is calculated relative to the total monocyte population. (d) A heat map reflecting the preference difference in cell distribution between CTL and YM101 groups, measured by odds ratios (OR). The heat map's color gradient spans from blue to red, indicating lower to higher preference, respectively. The *P*-values denote the significance of the difference in distribution.


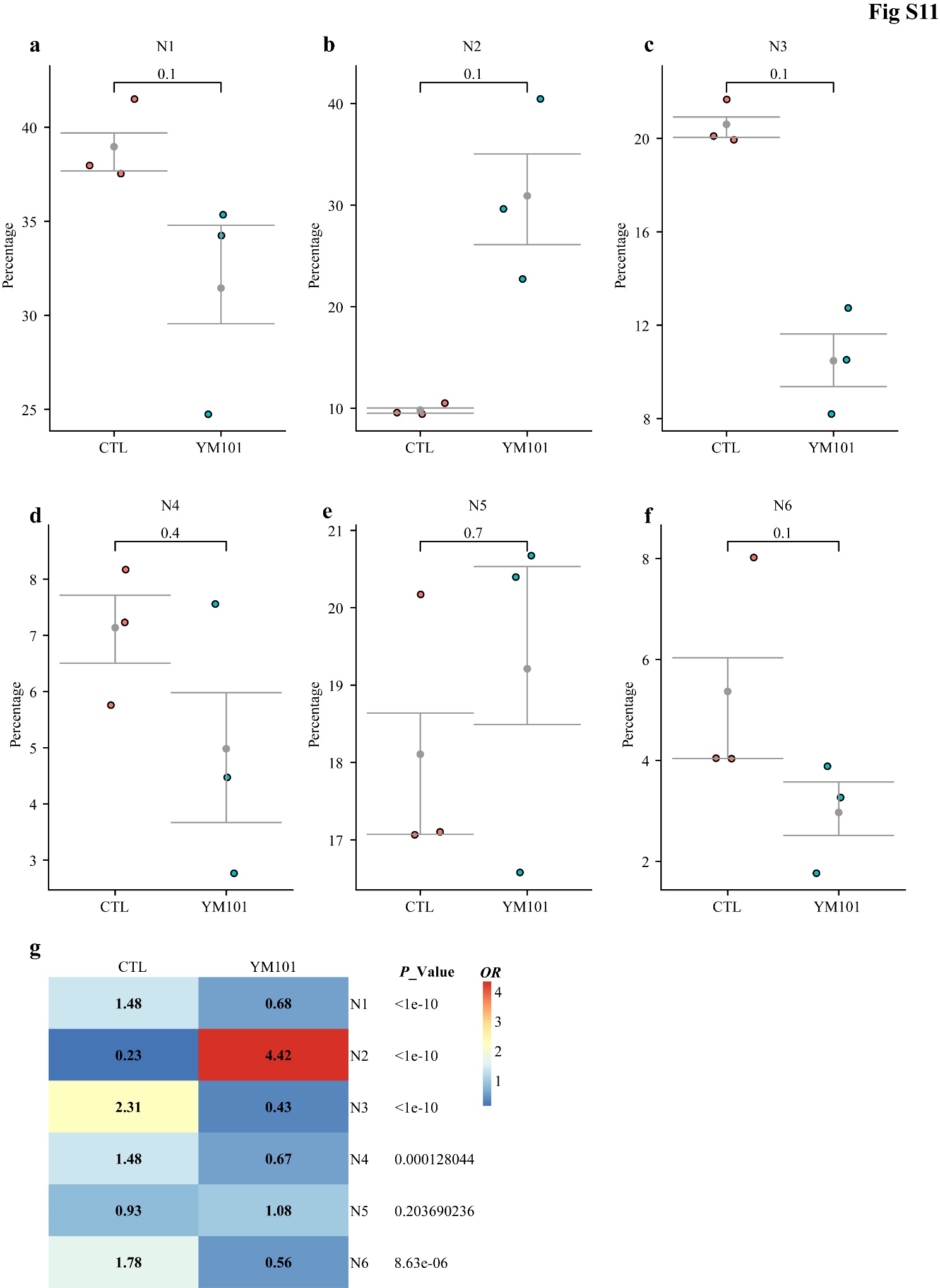


**Figure S11:** Preference differences in neutrophil subpopulation distribution between CTL and YM101 groups. (a-f) Scatter plots illustrating the percentages of various neutrophil subpopulations in the CTL and YM101 treatment groups. The subpopulations are as follows: (a) N1, (b) N2, (c) N3, (d) N4, (e) N5, (f) N6. Each point represents an individual measurement from distinct samples. The percentage of each neutrophil subset is calculated relative to the total neutrophil population. (g) Heat map reflecting the preference difference in cell distribution between CTL and YM101 groups, measured by odds ratios (OR). The heat map's color gradient spans from blue to red, indicating lower to higher preference, respectively. The *P*-values denote the significance of the difference in distribution.


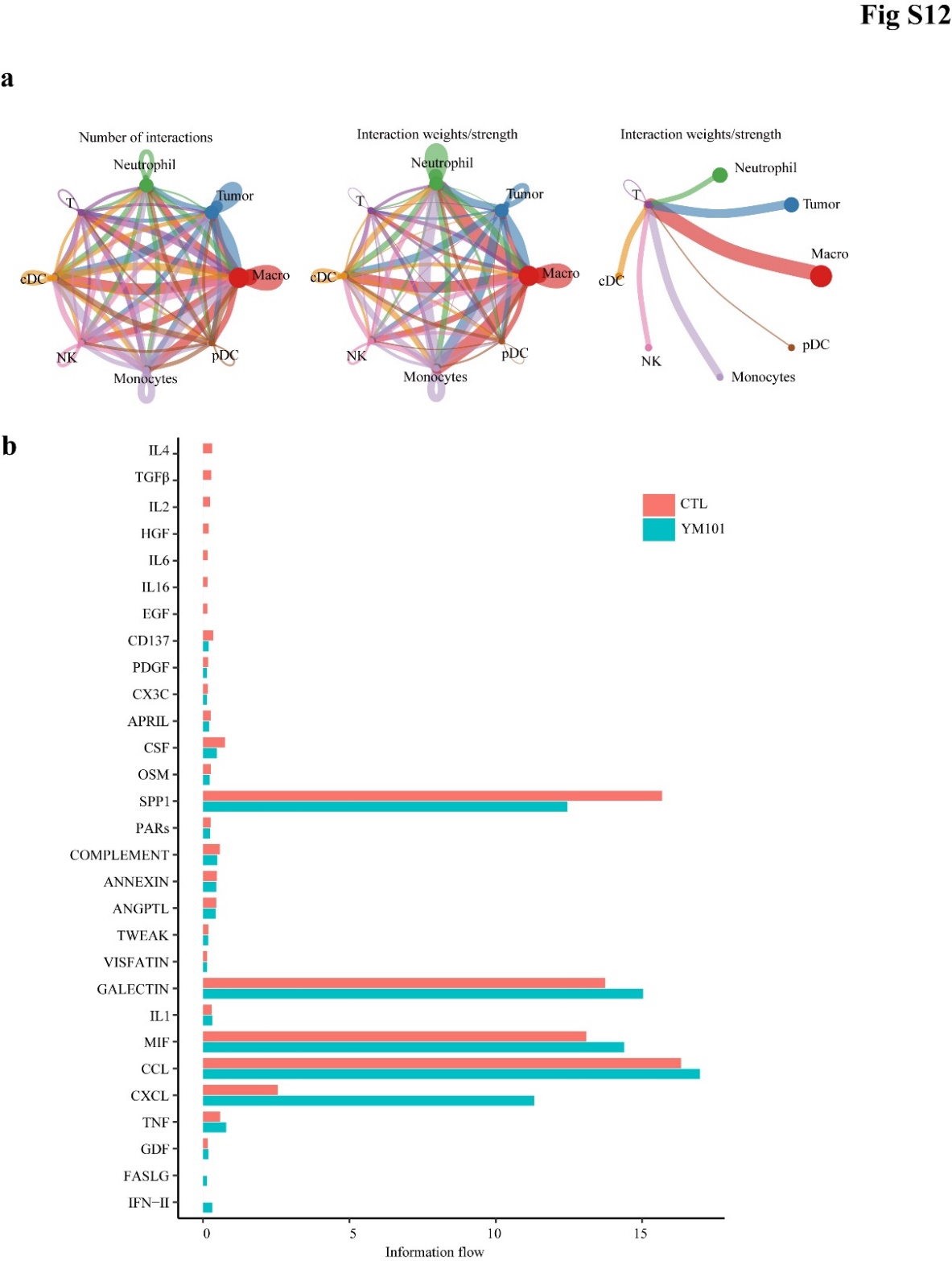


**Figure S12.** Cell communication prediction revealed by *CellChat*. (a) The visual summary of intercellular communications is presented, with directional cues provided by arrow and edge colors. The size of the circles corresponds to the cell count within each group. The thickness of edges signifies both the quantity and the intensity of interactions between these populations, while looped connections represent specific cell types. (b) Bar graphs depicting multiple signaling pathways. The signaling pathways appearing in red indicate the information flow of the CTL group, while those in blue are the information flow of the YM101 group.


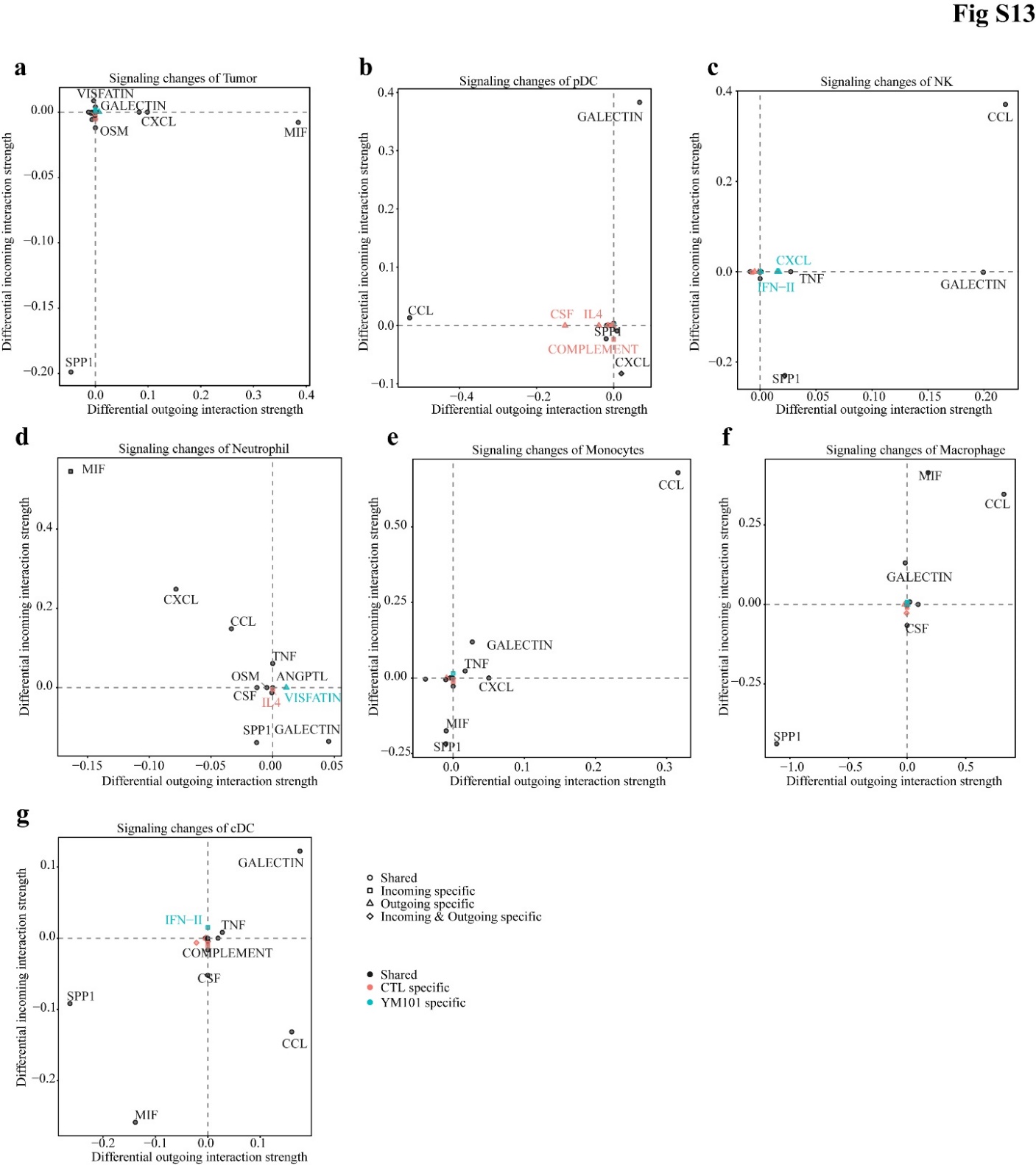


**Figure S13.** Scatter plots presenting differential outgoing and incoming signaling patterns of multiple components in the TME. (a) Differential signaling patterns of tumor cells in CTL and YM101 groups. (b) Differential signaling patterns of pDCs in CTL and YM101 groups. (c) Differential signaling patterns of NKs in CTL and YM101 groups. (d) Differential signaling patterns of neutrophils in CTL and YM101 groups. (e) Differential signaling patterns of monocytes in CTL and YM101 groups. (f) Differential signaling patterns of macrophages in CTL and YM101 groups. (g) Differential signaling patterns of cDCs in CTL and YM101 groups. The signaling pathways colored red were specific in the CTL group, and these colored blue pathways were specific in the YM101 group.


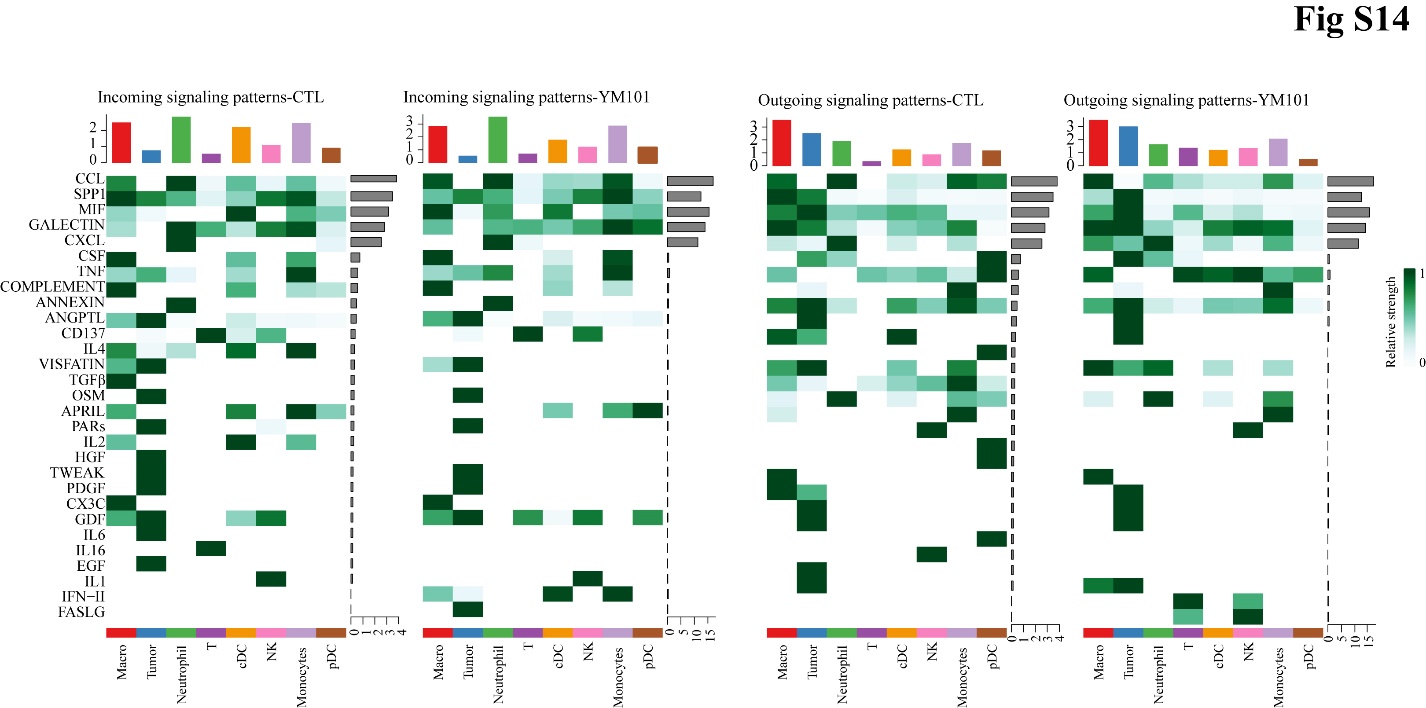
**Figure S14.** Heatmaps showing the strength of incoming and outgoing signaling flows of multiple components in the TME.


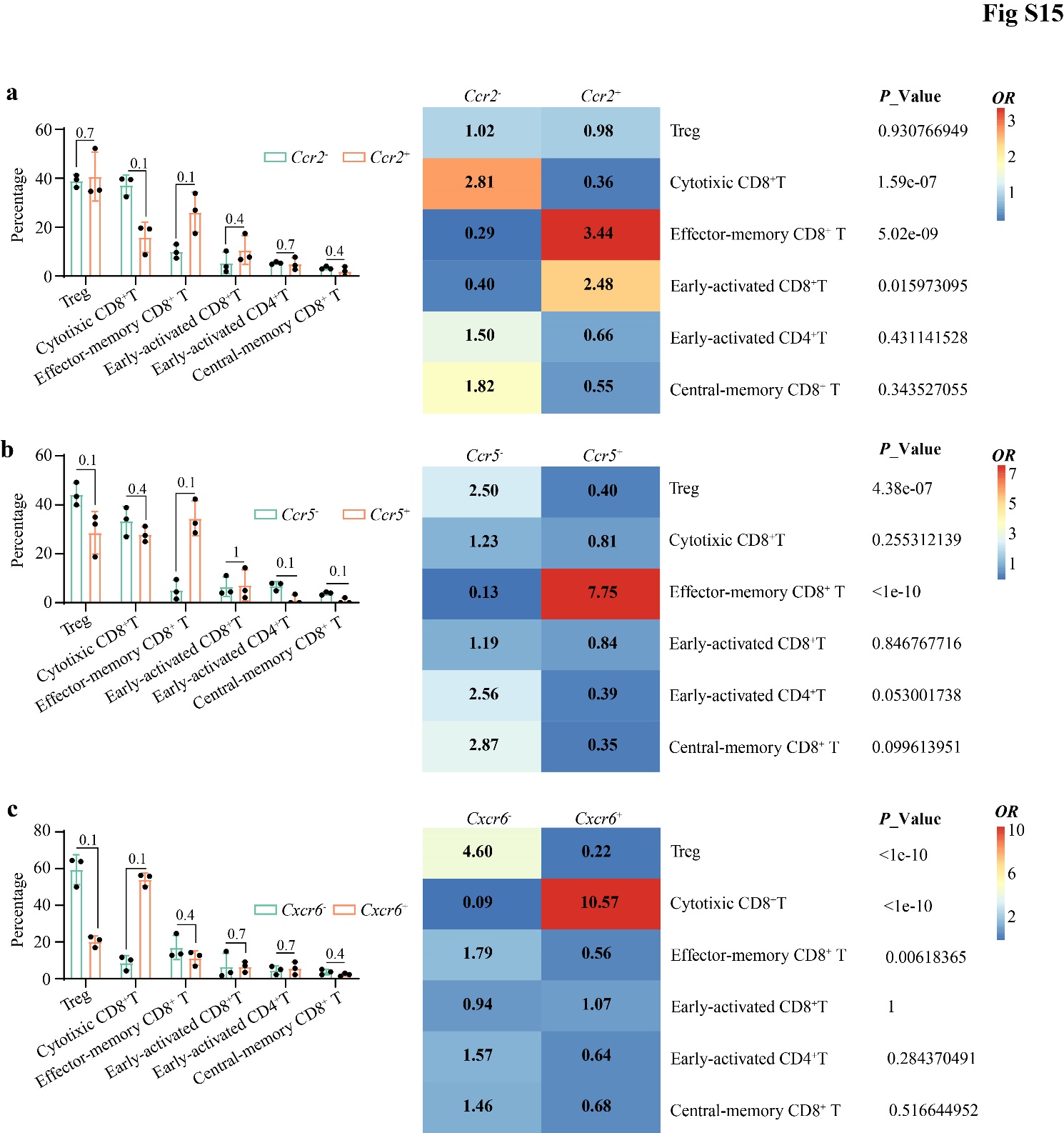
**Figure S15.** The preference difference in T cell distribution between *Ccr2*^+^ vs. *Ccr2*^-^ T cells, *Ccr5*^+^ vs. *Ccr5*^-^ T cells, and *Cxcr6*^+^ vs. *Cxcr6*^-^ T cells. (a) The left panel shows the percentage of each T-cell subset within *Ccr2*^+^ and *Ccr2*^-^ T cells. The right panel shows the preference difference in cell distribution between *Ccr2*^+^ vs. *Ccr2*^-^ T cells, measured by odds ratios (OR). (b) The left panel shows the percentage of each T-cell subset within *Ccr5*^+^ and *Ccr5*^-^ T cells. The right panel shows the preference difference in cell distribution between *Ccr5*^+^ vs. *Ccr5*^-^ T cells. (c) The left panel shows the percentage of each T-cell subset within *Cxcr6*^+^ and *Cxcr6*^-^ T cells. The right panel shows the preference difference in cell distribution between *Cxcr6*^+^ vs. *Cxcr6*^-^ T cells. The heat map's color gradient spans from blue to red, indicating lower to higher preference, respectively. The *P*-values denote the significance of the difference in distribution.


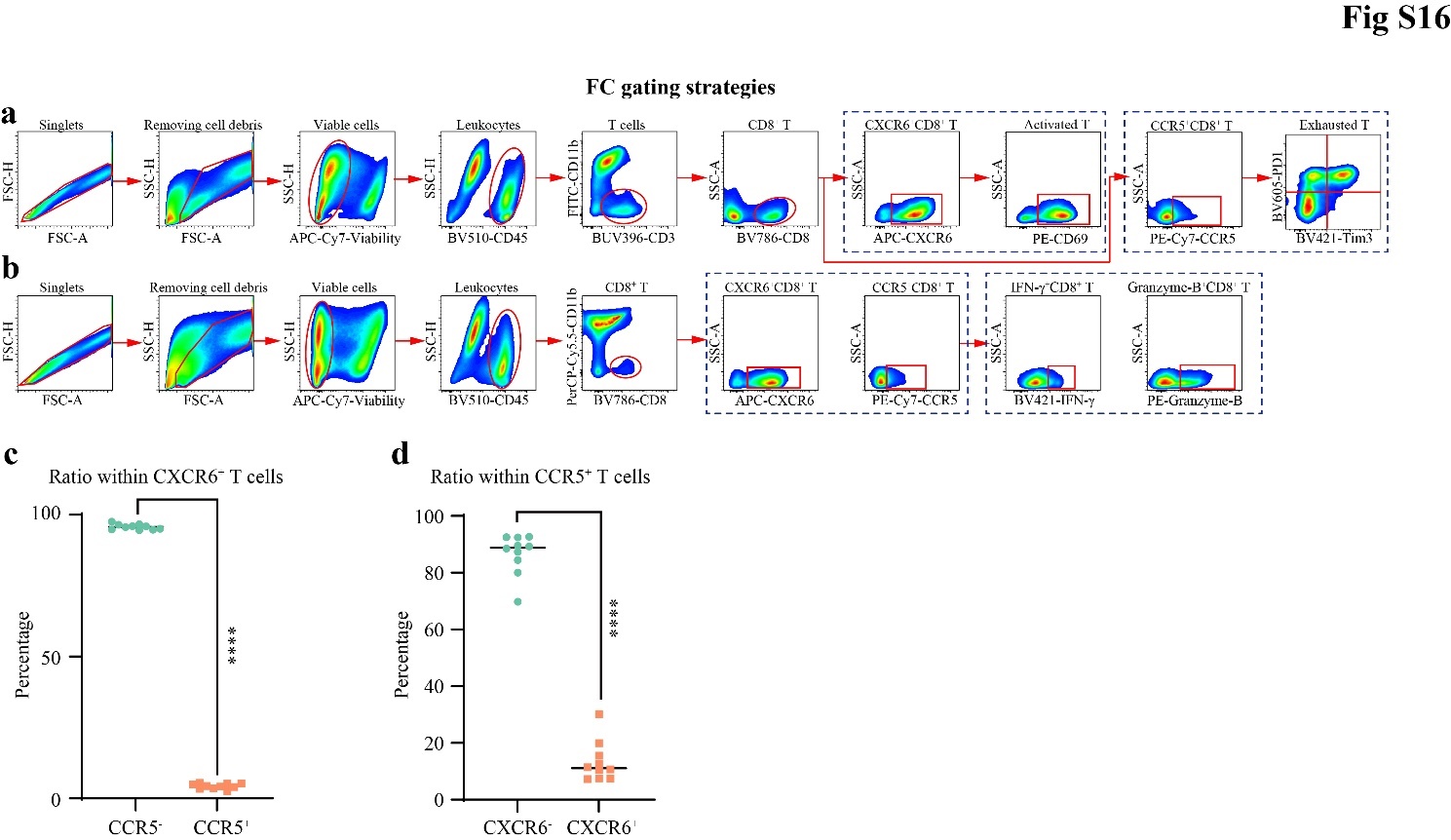


**Figure S16: Flow cytometry to explore the features of CXCR6^+^ and CCR5^+^ T cells.** (a-b) Gating strategies were employed to identify tumor-infiltrating CXCR6^+^ and CCR5^+^ T cells in CT26 tumors undergoing YM101 treatment. (c-d) The expression levels of CXCR6 within CCR5^+^ T cells and CCR5 within CXCR6^+^ T cells. Scatter plots are based on the flow cytometry data of YM101-treated CT26 tumors.


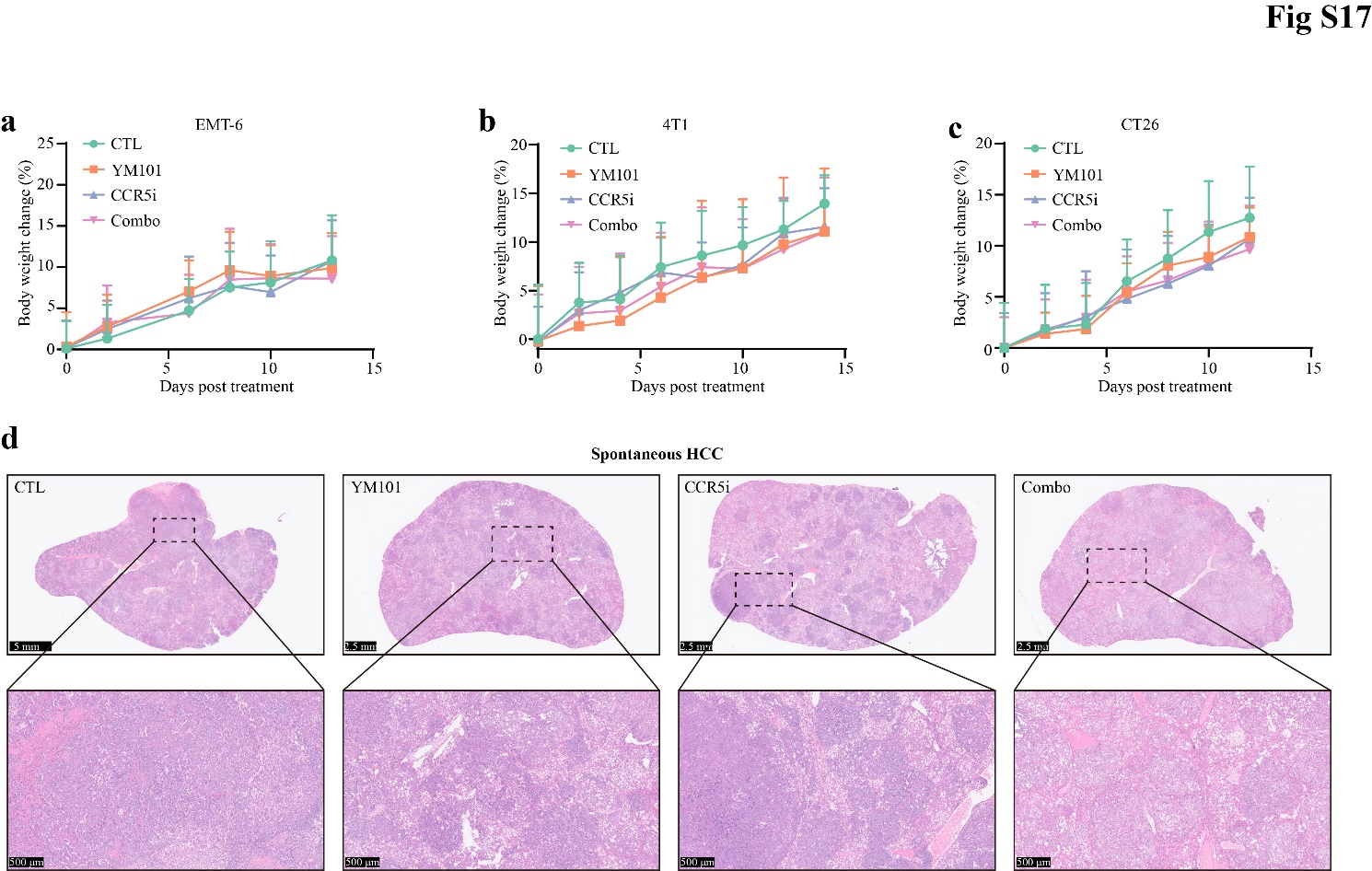
**Figure S17:** (a-c) The weight change curves of tumor-bearing mice. (d) The representative images nodular and diffuse tumors in spontaneous HCC model (H&E staining).

**Figure S18: Flow cytometry gating strategies.**
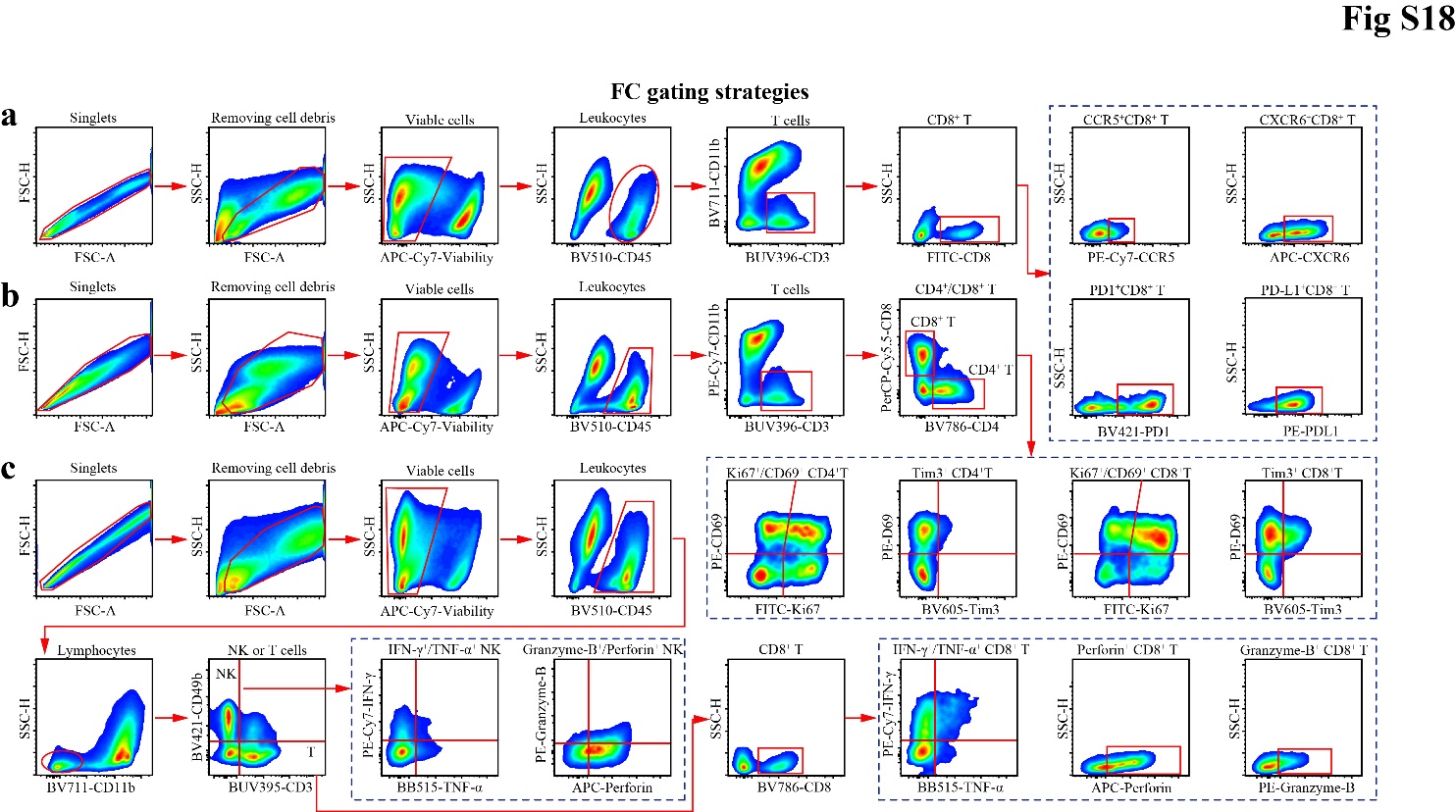
 Gating strategies were employed to identify tumor-infiltrating T cells and NK cells in EMT-6 tumors.


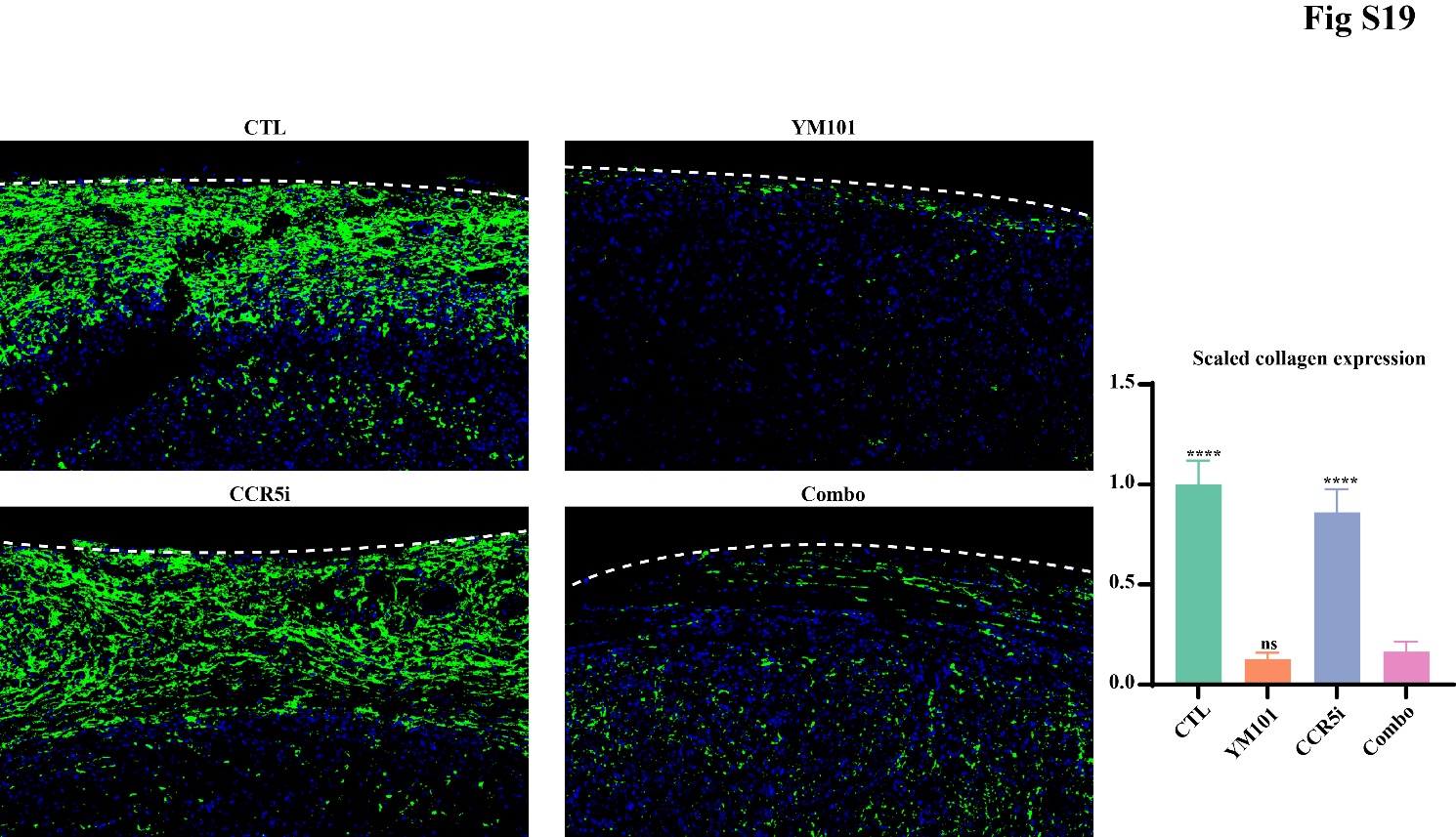


**Figure S19.** The influence of Maraviroc combined with YM101 treatment on peritumoral collagen generation in the EMT-6 model. Representative images of immunofluorescent staining showing peritumoral collagen deposition. **P* < 0.05 indicates a significant difference compared to the Maraviroc combined with YM101 group.


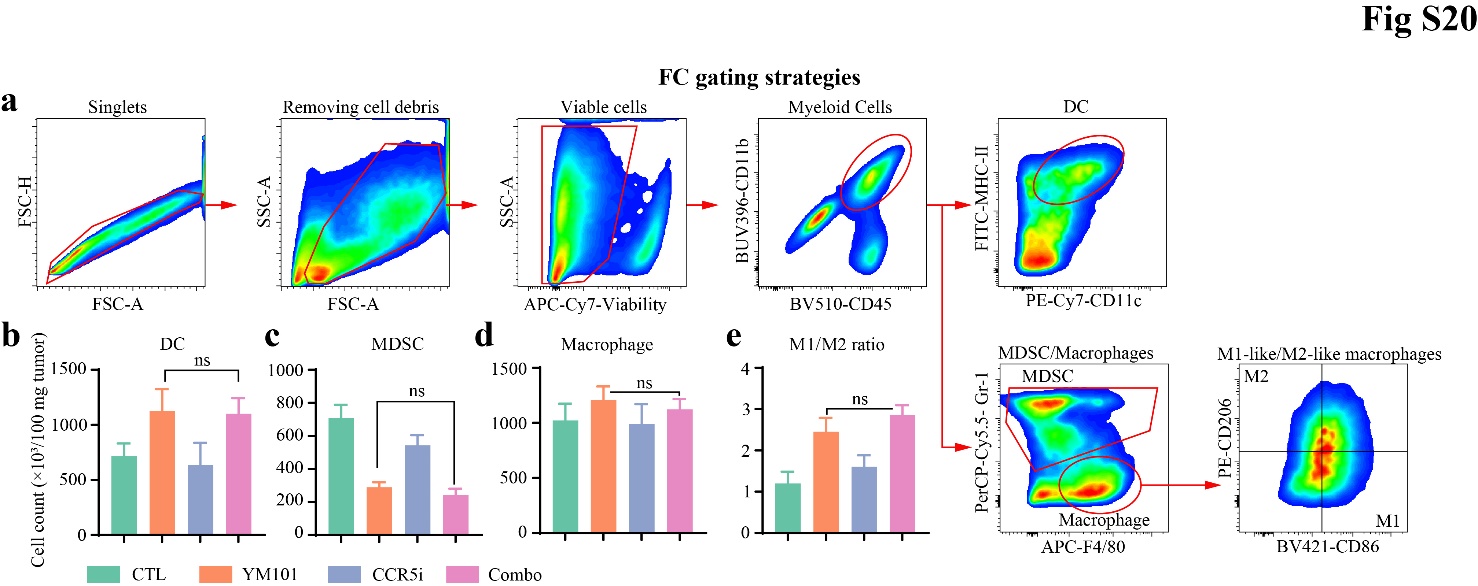


**Figure S20:** The influences of the combination therapy on tumor-infiltrating myeloid cells in EMT-6 tumors. (a) Flow cytometry gating strategies were employed to identify tumor-infiltrating DCs, myeloid-derived suppressor cells (MDSCs), and macrophages. (b-d) Quantitative analysis of the number of tumor-infiltrating DCs, MDSCs, and macrophages. (e) Quantitative analysis of the ratio of M1-like to M2-like macrophages.


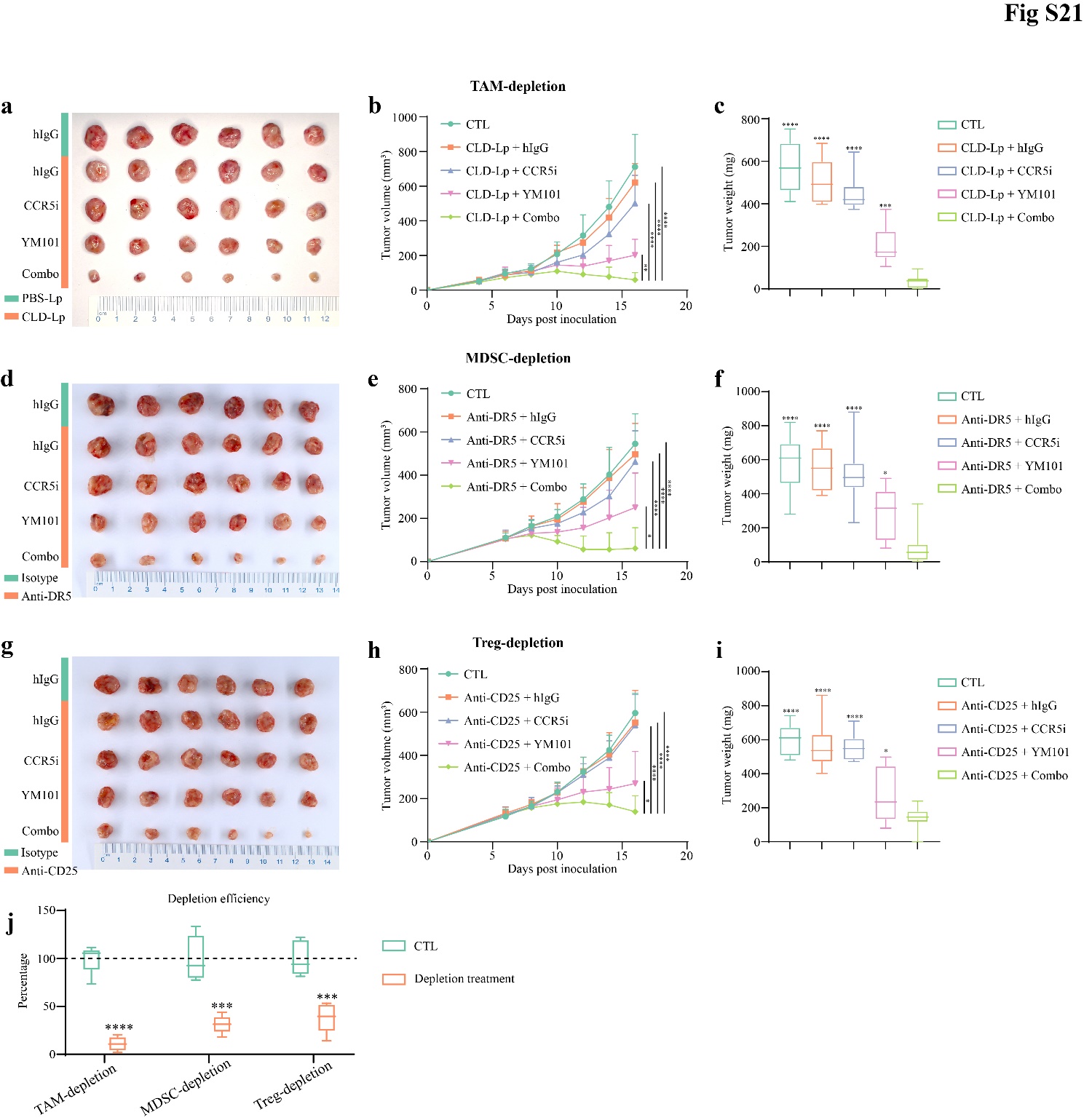


**Figure S21:** The effects of TAM, MDSC, and Treg depletion on the synergistic effect of CCR5 inhibitor and YM101. Tumor growth curves of Maraviroc combined with YM101 in (a-c) TAM-, (d-f) MDSC-, and (g-i) Treg-depleted EMT-6 models. (j) Flow cytometry analysis confirming the efficiency of cell depletion in the EMT-6 model. Depletion efficiency was assessed by comparing the relative cell numbers after depletion treatment to those in the CTL group.

**Table S1: *P* values of pathways or GO terms in enrichment analysis of tumor cells.**

| **HALLMARK** | **CTL** | **YM101** |
| --- | --- | --- |
| HALLMARK_INFLAMMATORY_RESPONSE | 0.001296 | 4.87e-05 |
| HALLMARK_INTERFERON_ALPHA_RESPONSE | 0.000747 | 0.000521 |
| HALLMARK_INTERFERON_GAMMA_RESPONSE | 6.16e-07 | 5.55e-07 |
| HALLMARK_TNFA_SIGNALING_VIA_NFKB | 0.002389 | 0.000262 |
| HALLMARK_IL2_STAT5_SIGNALING | 0.010472 | 0.003128 |
| HALLMARK_IL6_JAK_STAT3_SIGNALING | 0.007446 | 0.00101 |
| HALLMARK_APOPTOSIS | 0.080484 | 0.051835 |
| HALLMARK_P53_PATHWAY | 0.078526 | 0.055432 |
| HALLMARK_EPITHELIAL_MESENCHYMAL_TRANSITION | 0.229186 | 0.230043 |
| **KEGG PATHWAY** |  |  |
| KEGG_ANTIGEN_PROCESSING_AND_PRESENTATION | 0.000743 | 0.000422 |
| KEGG_VEGF_SIGNALING_PATHWAY | 0.5233 | 0.502996 |
| KEGG_MTOR_SIGNALING_PATHWAY | 0.344142 | 0.33642 |
| KEGG_ERBB_SIGNALING_PATHWAY | 0.292847 | 0.27314 |
| KEGG_TGF_BETA_SIGNALING_PATHWAY | 0.395476 | 0.354259 |
| KEGG_CYTOKINE_CYTOKINE_RECEPTOR_INTERACTION | 0.028308 | 0.004621 |
| KEGG_CYTOSOLIC_DNA_SENSING_PATHWAY | 0.027783 | 0.011895 |

**Table S2: *P* values of pathways or GO terms in enrichment analysis of T cells.**

| **HALLMARK** | **CTL** | **YM101** |
| --- | --- | --- |
| HALLMARK_INFLAMMATORY_RESPONSE | 0.289752 | 0.334348 |
| HALLMARK_INTERFERON_ALPHA_RESPONSE | 0.310705 | 0.31766 |
| HALLMARK_INTERFERON_GAMMA_RESPONSE | 0.009519 | 0.033925 |
| HALLMARK_MYC_TARGETS_V2 | 0.008658 | 0.013255 |
| HALLMARK_E2F_TARGETS | 0.057305 | 0.082492 |
| HALLMARK_IL6_JAK_STAT3_SIGNALING | 0.177558 | 0.218384 |
| HALLMARK_TNFA_SIGNALING_VIA_NFKB | 0.104904 | 0.102633 |
| HALLMARK_TGF_BETA_SIGNALING | 0.190019 | 0.181314 |
| **KEGG PATHWAY** |  |  |
| KEGG_APOPTOSIS | 0.056854 | 0.074377 |
| KEGG_DNA_REPLICATION | 0.107822 | 0.140462 |
| KEGG_NATURAL_KILLER_CELL_MEDIATED_CYTOTOXICITY | 0.062584 | 0.074286 |
| KEGG_CYTOKINE_CYTOKINE_RECEPTOR_INTERACTION | 0.20366 | 0.160823 |
| KEGG_CHEMOKINE_SIGNALING_PATHWAY | 0.314742 | 0.312827 |
| KEGG_NITROGEN_METABOLISM | 0.048845 | 0.038499 |
| KEGG_NICOTINATE_AND_NICOTINAMIDE_METABOLISM | 0.126885 | 0.091106 |
| KEGG_GLYCINE_SERINE_AND_THREONINE_METABOLISM | 0.131881 | 0.071724 |
| KEGG_HISTIDINE_METABOLISM | 0.23752 | 0.258449 |

**Table S3: *P* values of pathways or GO terms in enrichment analysis of Macrophages.**

| **HALLMARK** | **CTL** | **YM101** |
| --- | --- | --- |
| HALLMARK_INFLAMMATORY_RESPONSE | 5.07e-07 | 1.25e-05 |
| HALLMARK_INTERFERON_ALPHA_RESPONSE | 3.00e-05 | 0.000189 |
| HALLMARK_INTERFERON_GAMMA_RESPONSE | 1.59e-08 | 3.55e-06 |
| HALLMARK_IL2_STAT5_SIGNALING | 0.001702 | 0.004637 |
| HALLMARK_IL6_JAK_STAT3_SIGNALING | 1.38e-05 | 0.000209 |
| HALLMARK_TNFA_SIGNALING_VIA_NFKB | 0.0008 | 0.001552 |
| **KEGG PATHWAY** |  |  |
| KEGG_ANTIGEN_PROCESSING_AND_PRESENTATION | 0.003903 | 0.007891 |
| KEGG_TOLL_LIKE_RECEPTOR_SIGNALING_PATHWAY | 0.003748 | 0.008794 |
| KEGG_CYTOSOLIC_DNA_SENSING_PATHWAY | 0.129055 | 0.129536 |
| KEGG_RIG_I_LIKE_RECEPTOR_SIGNALING_PATHWAY | 0.006269 | 0.00991 |
| KEGG_NOD_LIKE_RECEPTOR_SIGNALING_PATHWAY | 0.007421 | 0.013968 |
| KEGG_JAK_STAT_SIGNALING_PATHWAY | 0.000513 | 0.001891 |
| KEGG_CHEMOKINE_SIGNALING_PATHWAY | 0.000996 | 0.001629 |
| KEGG_CYTOKINE_CYTOKINE_RECEPTOR_INTERACTION | 0.000349 | 0.000696 |

**Table S4: *P* values of pathways or GO terms in enrichment analysis of Macrophage subsets.**

| **HALLMARK** | M2-*Mmp12* | M2-*Siglec1* | M2-*Fn1* | M2-*Rsad2* | M2-*Malat1* | M2-*Mmp9* | M2-*S100a8/9* | M2-*Sparc* | M1-*Il1b* | Macro-prolif |
| --- | --- | --- | --- | --- | --- | --- | --- | --- | --- | --- |
| HALLMARK_INFLAMMATORY_RESPONSE | 0.255272 | 0.000106 | 2.48e-06 | 0.020527 | 0.181411 | 2.48e-06 | 0.821965 | 0.00289 | 0.000105 | 1.58e-16 |
| HALLMARK_INTERFERON_ALPHA_RESPONSE | 8.61e-08 | 0.09978 | 1.69e-10 | 4.98e-05 | 0.103549 | 0.018523 | 0.675011 | 0.481319 | 7.05e-05 | 1.05e-06 |
| HALLMARK_INTERFERON_GAMMA_RESPONSE | 1.80e-08 | 0.441193 | 5.90e-14 | 4.16e-05 | 0.912462 | 0.006575 | 0.567211 | 0.035268 | 2.22e-06 | 6.35e-08 |
| HALLMARK_ALLOGRAFT_REJECTION | 4.06e-07 | 0.578332 | 9.63e-05 | 0.833429 | 0.159647 | 0.000241 | 0.184495 | 0.026399 | 0.001259 | 2.41e-06 |
| HALLMARK_COMPLEMENT | 0.00797 | 0.774916 | 0.000407 | 0.027368 | 0.766704 | 0.003816 | 0.101793 | 0.018279 | 0.482355 | 1.59e-09 |
| HALLMARK_TNFA_SIGNALING_VIA_NFKB | 0.151408 | 3.81e-08 | 0.003174 | 0.20752 | 0.006407 | 2.63e-06 | 0.011795 | 0.272454 | 0.006138 | 4.94e-10 |
| HALLMARK_IL6_JAK_STAT3_SIGNALING | 0.102804 | 0.010585 | 5.64e-08 | 0.006116 | 0.747215 | 2.60e-07 | 0.610208 | 0.003286 | 2.39e-05 | 6.32e-09 |

**Table S5: *P* values of pathways or GO terms in enrichment analysis of cDCs.**

| **HALLMARK** | **CTL** | **YM101** |
| --- | --- | --- |
| HALLMARK_INTERFERON_ALPHA_RESPONSE | 9.27e-05 | 0.000391 |
| HALLMARK_INTERFERON_GAMMA_RESPONSE | 5.26e-07 | 4.68e-05 |
| HALLMARK_IL6_JAK_STAT3_SIGNALING | 0.646684 | 0.724518 |
| HALLMARK_NOTCH_SIGNALING | 0.211119 | 0.173738 |
| HALLMARK_HYPOXIA | 0.076326 | 0.040077 |
| HALLMARK_TGF_BETA_SIGNALING | 0.111644 | 0.124331 |
| **KEGG PATHWAY** |  |  |
| KEGG_ANTIGEN_PROCESSING_AND_PRESENTATION | 0.000434 | 0.001748 |
| KEGG_ALLOGRAFT_REJECTION | 7.48e-05 | 0.000105 |
| KEGG_WNT_SIGNALING_PATHWAY | 0.065429 | 0.097491 |
| KEGG_MTOR_SIGNALING_PATHWAY | 0.002464 | 0.001301 |
| KEGG_VEGF_SIGNALING_PATHWAY | 0.024555 | 0.04154 |
| KEGG_RIG_I_LIKE_RECEPTOR_SIGNALING_PATHWAY | 0.421857 | 0.465555 |
| KEGG_TOLL_LIKE_RECEPTOR_SIGNALING_PATHWAY | 0.554872 | 0.634042 |
| KEGG_JAK_STAT_SIGNALING_PATHWAY | 0.648386 | 0.710338 |

**Table S6: *P* values of pathways or GO terms in enrichment analysis of cDC subsets.**

| **KEGG** | cDC1_*Clec9a* | cDC1_*Ccl22* | cDC2_*Itgax* | cDC2_*Cd209a* | cDC-*Lyz2* | cDC-*S100a8/9* |
| --- | --- | --- | --- | --- | --- | --- |
| KEGG_ANTIGEN_PROCESSING_AND_PRESENTATION | 0.57 | 0.83 | 0.60 | 0.16 | 0.05 | 0.12 |

**Table S7: *P* values of pathways or GO terms in enrichment analysis of Monocytes.**

| **HALLMARK** | **CTL** | **YM101** |
| --- | --- | --- |
| HALLMARK_INFLAMMATORY_RESPONSE | 0.17552 | 0.166952 |
| HALLMARK_INTERFERON_ALPHA_RESPONSE | 0.000144 | 0.002139 |
| HALLMARK_INTERFERON_GAMMA_RESPONSE | 3.25e-08 | 2.52e-05 |
| HALLMARK_TNFA_SIGNALING_VIA_NFKB | 0.072177 | 0.026095 |
| HALLMARK_IL6_JAK_STAT3_SIGNALING | 0.060163 | 0.05783 |
| HALLMARK_IL2_STAT5_SIGNALING | 0.497867 | 0.455393 |
| HALLMARK_TGF_BETA_SIGNALING | 0.201689 | 0.195706 |
| **KEGG PATHWAY** |  |  |
| KEGG_ANTIGEN_PROCESSING_AND_PRESENTATION | 0.298859 | 0.315345 |
| KEGG_CYTOKINE_CYTOKINE_RECEPTOR_INTERACTION | 0.020219 | 0.030175 |
| KEGG_CYTOSOLIC_DNA_SENSING_PATHWAY | 0.108559 | 0.136036 |
| KEGG_RIG_I_LIKE_RECEPTOR_SIGNALING_PATHWAY | 0.305196 | 0.359442 |
| KEGG_CHEMOKINE_SIGNALING_PATHWAY | 0.347362 | 0.29388 |
| KEGG_NOD_LIKE_RECEPTOR_SIGNALING_PATHWAY | 0.239703 | 0.203769 |
| KEGG_FC_GAMMA_R_MEDIATED_PHAGOCYTOSIS | 0.780227 | 0.755289 |

**Table S8: *P* values of pathways or GO terms in enrichment analysis of Monocyte subsets.**

| **HALLMARK** | **Mono_*Ly6c2*** | **Mono_*Cd74*** | **Mono_*S100a8/9*** |
| --- | --- | --- | --- |
| HALLMARK_HYPOXIA | 0.00054 | 0.908237 | 0.016776 |
| HALLMARK_APOPTOSIS | 0.553861 | 0.290967 | 0.622644 |
| HALLMARK_TGF_BETA_SIGNALING | 0.023617 | 0.201304 | 0.109756 |
| **KEGG PATHWAY** |  |  |  |
| KEGG_ANTIGEN_PROCESSING_AND_PRESENTATION | 0.105318 | 0.043165 | 0.013 |
| KEGG_CYTOSOLIC_DNA_SENSING_PATHWAY | 0.586046 | 0.421684 | 0.299536 |

**Table S9: *P* values of pathways or GO terms in enrichment analysis of Neutrophils.**

| **HALLMARK** | **CTL** | **YM101** |
| --- | --- | --- |
| HALLMARK_MYC_TARGETS_V1 | 3.58e-14 | 1.93e-14 |
| HALLMARK_MYC_TARGETS_V2 | 9.73e-12 | 4.63e-12 |
| HALLMARK_INFLAMMATORY_RESPONSE | 9.283e-05 | 0.000111 |
| HALLMARK_INTERFERON_ALPHA_RESPONSE | 1.97e-09 | 2.17e-07 |
| HALLMARK_INTERFERON_GAMMA_RESPONSE | 2.767e-11 | 1.97e-08 |
| HALLMARK_TNFA_SIGNALING_VIA_NFKB | 0.045178 | 0.054552 |
| HALLMARK_IL6_JAK_STAT3_SIGNALING | 0.080179 | 0.076823 |
| HALLMARK_IL2_STAT5_SIGNALING | 3.30e-07 | 3.52e-07 |
| **KEGG PATHWAY** |  |  |
| KEGG_CYTOKINE_CYTOKINE_RECEPTOR_INTERACTION | 0.000667 | 0.00038 |
| KEGG_CHEMOKINE_SIGNALING_PATHWAY | 0.179509 | 0.173631 |
| KEGG_CYTOSOLIC_DNA_SENSING_PATHWAY | 7.37e-05 | 0.000163 |
| KEGG_TOLL_LIKE_RECEPTOR_SIGNALING_PATHWAY | 0.00565 | 0.005458 |
| KEGG_NOD_LIKE_RECEPTOR_SIGNALING_PATHWAY | 0.008001 | 0.007265 |
| KEGG_RIG_I_LIKE_RECEPTOR_SIGNALING_PATHWAY | 0.007524 | 0.008463 |
| KEGG_JAK_STAT_SIGNALING_PATHWAY | 0.019365 | 0.022206 |
| KEGG_NATURAL_KILLER_CELL_MEDIATED_CYTOTOXICITY | 0.175866 | 0.154492 |

**Table S10: *P* values of pathways or GO terms in enrichment analysis of Neutrophil subsets.**

| **HALLMARK** | **N1** | **N2** | **N3** | **N4** | **N5** | **N6** |
| --- | --- | --- | --- | --- | --- | --- |
| HALLMARK_INFLAMMATORY_RESPONSE | 0.000148 | 0.000888 | 6.24e-07 | 0.000409 | 0.512191 | 0.00032 |
| HALLMARK_INTERFERON_ALPHA_RESPONSE | 2.30e-10 | 1.81e-10 | 2.43e-14 | 0.000948 | 0.003266 | 9.30e-07 |
| HALLMARK_INTERFERON_GAMMA_RESPONSE | 8.58e-09 | 3.39e-11 | 6.97e-14 | 0.000943 | 0.023514 | 7.17e-06 |
| HALLMARK_IL2_STAT5_SIGNALING | 8.998e-07 | 0.217114 | 0.000312 | 0.153238 | 0.100271 | 0.001189 |
| HALLMARK_IL6_JAK_STAT3_SIGNALING | 0.502085 | 0.191584 | 1.09e-05 | 0.995072 | 0.092158 | 0.083168 |
| HALLMARK_TNFA_SIGNALING_VIA_NFKB | 0.02433 | 0.625682 | 0.061044 | 0.001427 | 0.535043 | 0.532143 |
| **KEGG PATHWAY** |  |  |  |  |  |  |
| KEGG_CYTOKINE_CYTOKINE_RECEPTOR_INTERACTION | 0.106277 | 0.052676 | 0.053686 | 0.053708 | 0.405239 | 0.042689 |
| KEGG_CYTOSOLIC_DNA_SENSING_PATHWAY | 0.005094 | 0.030229 | 0.062475 | 0.978186 | 0.731239 | 0.007711 |
| KEGG_RIG_I_LIKE_RECEPTOR_SIGNALING_PATHWAY | 0.06047 | 0.025926 | 0.026389 | 0.255628 | 0.645216 | 0.008871 |
| KEGG_TOLL_LIKE_RECEPTOR_SIGNALING_PATHWAY | 0.405917 | 0.350714 | 0.005882 | 0.256538 | 0.722296 | 0.001578 |
| KEGG_NOD_LIKE_RECEPTOR_SIGNALING_PATHWAY | 0.000245 | 0.08002 | 0.000374 | 0.296228 | 0.787869 | 0.421074 |

**Table S11: *P* values of pathways or GO terms in enrichment analysis of *Ccr2*^+^ and *Ccr5*^+^ T cells.**

| **KEGG PATHWAY (*Ccr2*^+^ T cells)** | **CTL** | **YM101** |
| --- | --- | --- |
| KEGG_T_CELL_RECEPTOR_SIGNALING_PATHWAY | 0.034335 | 0.047606 |
| KEGG_NATURAL_KILLER_CELL_MEDIATED_CYTOTOXICITY | 0.017574 | 0.036129 |
| KEGG_DNA_REPLICATION | 0.1683 | 0.141849 |
| KEGG_JAK_STAT_SIGNALING_PATHWAY | 0.441333 | 0.432354 |
| KEGG_MTOR_SIGNALING_PATHWAY | 0.197889 | 0.131234 |
| **KEGG PATHWAY (*Ccr5*^+^ T cells)** |  |  |
| KEGG_T_CELL_RECEPTOR_SIGNALING_PATHWAY | 0.000848 | 0.000731 |
| KEGG_NATURAL_KILLER_CELL_MEDIATED_CYTOTOXICITY | 0.53399 | 0.478717 |
| KEGG_MAPK_SIGNALING_PATHWAY | 0.146074 | 0.236347 |
| KEGG_ERBB_SIGNALING_PATHWAY | 0.389088 | 0.457257 |
| KEGG_JAK_STAT_SIGNALING_PATHWAY | 0.412293 | 0.470437 |
| KEGG_RIG_I_LIKE_RECEPTOR_SIGNALING_PATHWAY | 0.001836 | 0.00691 |

**Table S12: The results of GSEA of** ***Ccr2*^+^, *Ccr5*^+^, and *Cxcr6*^+^ T cells (YM101 *v.s.* CTL).**

| **Term (*Ccr2*^+^ T cells)** | **NES** | **P value** |
| --- | --- | --- |
| Natural killer cell mediated cytotoxicity - KEGG | -1.440888983 | 0.037383178 |
| T cell receptor signaling pathway - KEGG | -1.627229454 | 0.00990099 |
| alpha-beta T cell activation - GO | -1.658493075 | 0.005319149 |
| Granzyme-mediated programmed cell death signaling pathway - GO | -1.992206069 | 0.002202643 |
| Natural killer cell mediated cytotoxicity - GO | -2.572091114 | 0.002267574 |
| T cell receptor signaling pathway - GO | -1.624956573 | 0.007228916 |
| **Term (*Ccr5*^+^ T cells)** |  |  |
| JAK-STAT signaling pathway - KEGG | -1.619520531 | 0.004175365 |
| T cell receptor signaling pathway - KEGG | -2.254883737 | 0.002309469 |
| alpha-beta T cell activation involved in immune response - GO | -2.026764685 | 0.002398082 |
| Calcineurin-NFAT signaling cascade - GO | -1.613495373 | 0.028634361 |
| **Term (*Cxcr6*^+^ T cells)** |  |  |
| Natural killer cell mediated cytotoxicity - KEGG | 1.970093572 | 0.003831418 |
| T cell receptor signaling pathway - KEGG | 1.289930707 | 0.088709677 |
| Cell killing - GO | 2.034523442 | 0.005102041 |
| Cytolysis - GO | 2.106579205 | 0.00273224 |
| Natural killer cell mediated cytotoxicity - GO | 2.693570937 | 0.003636364 |
| T cell mediated cytotoxicity - GO | 1.515054634 | 0.042622951 |

**Table S13: *P* values of pathways or GO terms in enrichment analysis of NK cells.**

| **HALLMARK** | **CTL** | **YM101** |
| --- | --- | --- |
| HALLMARK_INFLAMMATORY_RESPONSE | 0.009392 | 0.024188 |
| HALLMARK_INTERFERON_ALPHA_RESPONSE | 0.000387 | 0.004907 |
| HALLMARK_INTERFERON_GAMMA_RESPONSE | 1.09e-06 | 0.000623 |
| HALLMARK_TNFA_SIGNALING_VIA_NFKB | 3.82e-06 | 5.42e-06 |
| HALLMARK_IL2_STAT5_SIGNALING | 0.042334 | 0.030161 |
| HALLMARK_IL6_JAK_STAT3_SIGNALING | 0.003229 | 0.004485 |
| **KEGG PATHWAY** |  |  |
| KEGG_JAK_STAT_SIGNALING_PATHWAY | 0.008972 | 0.036049 |
| KEGG_TOLL_LIKE_RECEPTOR_SIGNALING_PATHWAY | 0.029805 | 0.088494 |
| KEGG_CYTOKINE_CYTOKINE_RECEPTOR_INTERACTION | 0.018254 | 0.031194 |
| KEGG_CHEMOKINE_SIGNALING_PATHWAY | 0.109837 | 0.131041 |
| KEGG_GRAFT_VERSUS_HOST_DISEASE | 4.26e-06 | 0.000342 |
| KEGG_B_CELL_RECEPTOR_SIGNALING_PATHWAY | 0.326285 | 0.390901 |
| KEGG_T_CELL_RECEPTOR_SIGNALING_PATHWAY | 0.265412 | 0.312965 |

**Table S14: Staining antibodies and auxiliary reagents used for the flow cytometry assays of animal tumor models.**

| **Antibody or auxiliary reagent** | **Catalog No.** | **Manufacturer** | **Working Dose** |
| --- | --- | --- | --- |
| Cell staining buffer | 420201 | BioLegend | - |
| Collagenase IV | C5138 | Sigma | 1 mg/ml |
| DNase I | D4527 | Sigma | 200 U/ml |
| Fixable Viability Dye eFluor™ 780 | 65-0865-18 | Thermo | 1: 200 |
| Foxp3 Transcription Factor Staining Buffer Set | 00-5523-00 | Thermo | - |
| Intracellular Fix/Perm Buffer Set | 88-8824-00 | Thermo | - |
| Leukocyte Activation Cocktail, with BD GolgiPlug | 550583 | BD | 1: 500 |
| Mouse CCL3 eFluor™ 660 antibody | 50-7532-80 | Thermo | 1: 100 |
| Mouse CCR5 PE-Cy7 antibody | 107017 | BioLegend | 1: 100 |
| Mouse CCR5 APC antibody | 107011 | BioLegend | 1: 100 |
| Mouse CD11b BUV395 antibody | 363-0112-82 | BioLegend | 1: 100 |
| Mouse CD11b BV711 antibody | 101242 | BioLegend | 1: 100 |
| Mouse CD11b FITC antibody | 11-0112-82 | Thermo | 1: 200 |
| Mouse CD11b PE-Cy7 antibody | 25-0112-82 | Thermo | 1: 100 |
| Mouse CD11b PerCP-Cy5.5 antibody | 45-0112-82 | Thermo | 1: 100 |
| mouse CD11b FITC antibody | 101205 | BioLegend | 1: 100 |
| Mouse CD11c PE-Cy7 antibody | 117318 | BioLegend | 1: 100 |
| Mouse CD16/32 blocking antibody | 14-0161-86 | Thermo | 1: 100 |
| Mouse CD206 PE antibody | 141706 | BioLegend | 1: 100 |
| Mouse CD25 BV421 antibody | 102043 | BioLegend | 1: 100 |
| Mouse CD3 BUV395 antibody | 563565 | BD | 1: 100 |
| Mouse CD4 BV786 antibody | 417-0042-82 | Thermo | 1: 100 |
| Mouse CD45-BV510 antibody | 563891 | BD | 1: 100 |
| Mouse CD45 PerCP/Cyanine5.5 antibody | 103132 | BioLegend | 1:100 |
| Mouse CD49b BV421 antibody | 563063 | BD | 1: 100 |
| Mouse CD69 PE antibody | 104507 | BioLegend | 1: 100 |
| Mouse CD8 BV786 antibody | 417-0081-82 | Thermo | 1: 100 |
| Mouse CD8 FITC antibody | 100705 | BioLegend | 1: 200 |
| Mouse CD8 PerCP-Cy5.5 antibody | 45-0081-82 | Thermo | 1: 100 |
| Mouse CD86 BV421 antibody | 105032 | BioLegend | 1: 100 |
| Mouse CXCR6 APC antibody | 151105 | BioLegend | 1: 100 |
| Mouse F4/80 APC antibody | 17-4801-82 | Thermo | 1: 100 |
| Mouse Foxp3 APC antibody | 17-5773-82 | Thermo | 1: 100 |
| Mouse Gr-1 PerCP-Cy5.5 antibody | 108427 | BioLegend | 1: 100 |
| Mouse Granzyme-B PE antibody | 12-8898-82 | Thermo | 1: 100 |
| Mouse IFN-γ BV421 antibody | 505830 | BioLegend | 1: 100 |
| Mouse IFN-γ PE-Cy7 antibody | 25-7311-82 | Thermo | 1: 100 |
| Mouse MHC-II FITC antibody | 107606 | BioLegend | 1: 200 |
| Mouse PD1 BV421 antibody | 135217 | BioLegend | 1: 100 |
| Mouse PD1 BV605 antibody | 135220 | BioLegend | 1: 100 |
| Mouse PD-L1 PE antibody | 558091 | BD | 1: 100 |
| Mouse Perforin APC antibody | 154404 | BioLegend | 1: 100 |
| Mouse Tim3 BV421 antibody | 134019 | BioLegend | 1: 100 |
| Mouse Tim3 BV605 antibody | 119721 | BioLegend | 1: 100 |
| Mouse TNF-α FITC antibody | 11-7321-81 | Thermo | 1: 200 |
| Mouse/Human Ki67 FITC antibody | 11-5698-82 | Thermo | 1: 200 |
